# Quantitative and Chemically Intuitive Evaluation of the Nature of M−L Bonds in Paramagnetic Compounds: Application of EDA‐NOCV Theory to Spin Crossover Complexes

**DOI:** 10.1002/chem.202002146

**Published:** 2020-09-24

**Authors:** Luca Bondì, Anna L. Garden, Paul Jerabek, Federico Totti, Sally Brooker

**Affiliations:** ^1^ Department of Chemistry and MacDiarmid Institute of Advanced Materials and Nanotechnology University of Otago PO Box 56 Dunedin 9054 New Zealand; ^2^ Department of Chemistry “Ugo Schiff” and INSTM Research Unit University of Florence 50019 Sesto Fiorentino Italy; ^3^ Centre for Theoretical Chemistry and Physics The New Zealand Institute for Advanced Study and the Institute for Natural and Mathematical Sciences Massey University Auckland New Zealand; ^4^ Department of Nanotechnology Helmholtz Centre for Materials and Coastal Research Max-Planck-Straße 1 21502 Geesthacht Germany

**Keywords:** M−L bonding, paramagnetism, spin crossover, theoretical chemistry

## Abstract

To improve understanding of **M−L** bonds in 3d transition metal complexes, analysis by energy decomposition analysis and natural orbital for chemical valence model (EDA‐NOCV) is desirable as it provides a full, quantitative and chemically intuitive ab initio description of the **M−L** interactions. In this study, a generally applicable fragmentation and computational protocol was established and validated by using octahedral spin crossover (SCO) complexes, as the transition temperature (*T*_1/2_) is sensitive to subtle changes in **M−L** bonding. Specifically, EDA‐NOCV analysis of Fe−N bonds in five [Fe^II^(***L*** ^***azine***^)_2_(NCBH_3_)_2_], in both low‐spin (LS) and paramagnetic high‐spin (HS) states led to: 1) development of a general, widely applicable, corrected M+L_6_ fragmentation, tested against a family of five LS [Fe^II^(***L*** ^***azine***^)_3_](BF_4_)_2_ complexes; this confirmed that three ***L*** ^***azine***^ are stronger ligands (Δ*E*_orb,σ+*π*_=−370 kcal mol^−1^) than **2** ***L*** ^***azine***^ **+2 NCBH_3_** (=−335 kcal mol^−1^), as observed. 2) Analysis of Fe−**L** bonding on LS→HS, reveals more ionic (Δ*E*_elstat_) and less covalent (Δ*E*_orb_) character (Δ*E*_elstat_:Δ*E*_orb_ 55:45 LS*→*64:36 HS), mostly due to a big drop in σ (Δ*E*_orb,σ_ ↓50 %; −310→−145 kcal mol^−1^), and a drop in π contributions (Δ*E*_orb,π_ ↓90 %; −30→−3 kcal mol^−1^). 3) Strong correlation of observed *T*_1/2_ and Δ*E*_orb,σ+π_, for both LS and HS families (*R* ^2^=0.99 LS, *R* ^2^=0.95 HS), but no correlation of *T*_1/2_ and ΔΔ*E*_orb,σ+π_(LS*‐*HS) (*R* ^2^=0.11). Overall, this study has established and validated an EDA‐NOCV protocol for **M−L** bonding analysis of any diamagnetic or paramagnetic, homoleptic or heteroleptic, octahedral transition metal complex. This new and widely applicable EDA‐NOCV protocol holds great promise as a predictive tool.

## Graphical abstract

**A generally applicable** fragmentation and computational protocol for EDA‐NOCV **M−L** bonding analysis of any diamagnetic or paramagnetic, homoleptic or heteroleptic, octahedral complex is established and validated in the first EDA‐NOCV analysis of spin crossover‐active complexes. As expected the combination of the σ and π bonding (Δ*E*_orb,σ+π_) in the Fe−**L** bonds correlates very well with the observed switching temperature (*T*_1/2_).

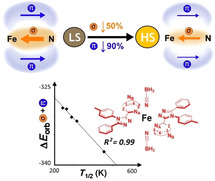

## Introduction

The function of metalloenzymes,^[1]^ catalysts^[2]^ and materials^[3]^ is often utterly dependent on the finely tuned properties of a first‐row transition metal ion(s), M, at the active site. Fine‐tuning the **M−L** interactions^[4]^—and hence the ligand field imposed on M—in a predictable manner^[5]^ is generally done by a series of small modifications to a particular ligand skeleton, such as varying a substituent or exchanging a CH for an N atom in a heterocycle, within a family of related complexes.[^5b^, ^5k^, ^6^] We have trialled a new in silico approach to improving our detailed understanding of **M−L** interactions in *any* octahedral complex,^[7]^ in particular aiming to address this in paramagnetic 3d complexes.

Bold formatting is used to highlight the fragmentation scheme used for the **M−L** complex. Italics are used for low spin (*LS*) and high spin (*HS*) state abbreviations. Bold and italic formatting is used for the organic ligand family, ***L*** ^***azine***^, used in the complexes investigated in this study.

Specifically, energy decomposition analysis (EDA) and natural orbital for chemical valence theory (NOCV)^[8]^ were used in combination^[9]^ in order to provide a full, quantitative and chemically intuitive ab initio description of the **M−L** interactions during bond formation: the various contributions to the total interaction energy (Δ*E*_int_) are assessed by the use of EDA, and then a breakdown of the orbital contribution (Δ*E*_orb_) to quantitatively assess the **M−L** bond character is achieved by the use of the NOCV scheme.

Whilst EDA‐NOCV methodology has been extensively used to study diamagnetic systems,^[10]^ it has rarely been applied to paramagnetic transition metal complexes,^[11]^ lanthanide/actinide complexes,^[12]^ or indeed to other open‐shell radical systems;^[13]^ the somewhat related ALMO‐EDA has been used to investigate pressure‐induced SCO.^[14]^ Nevertheless there were no systematic studies that could provide guidance with respect to a general fragmentation scheme (i.e., **M^n+^**+**L_6_** vs. **ML_5_** ^**n+**^+**L** for a general **ML_6_** complex) suitable for EDA‐based bonding analyses and direct comparison of *any* metal complex—so we rigorously, and successfully, address this issue herein.

Our first, and key, step was therefore to establish a suitable, generally applicable fragmentation and computational protocol for the EDA‐NOCV analysis of any diamagnetic or paramagnetic, homoleptic or heteroleptic, octahedral complex. To do this, a test system that enables validation of the outcomes must be chosen.

**Figure 1:**
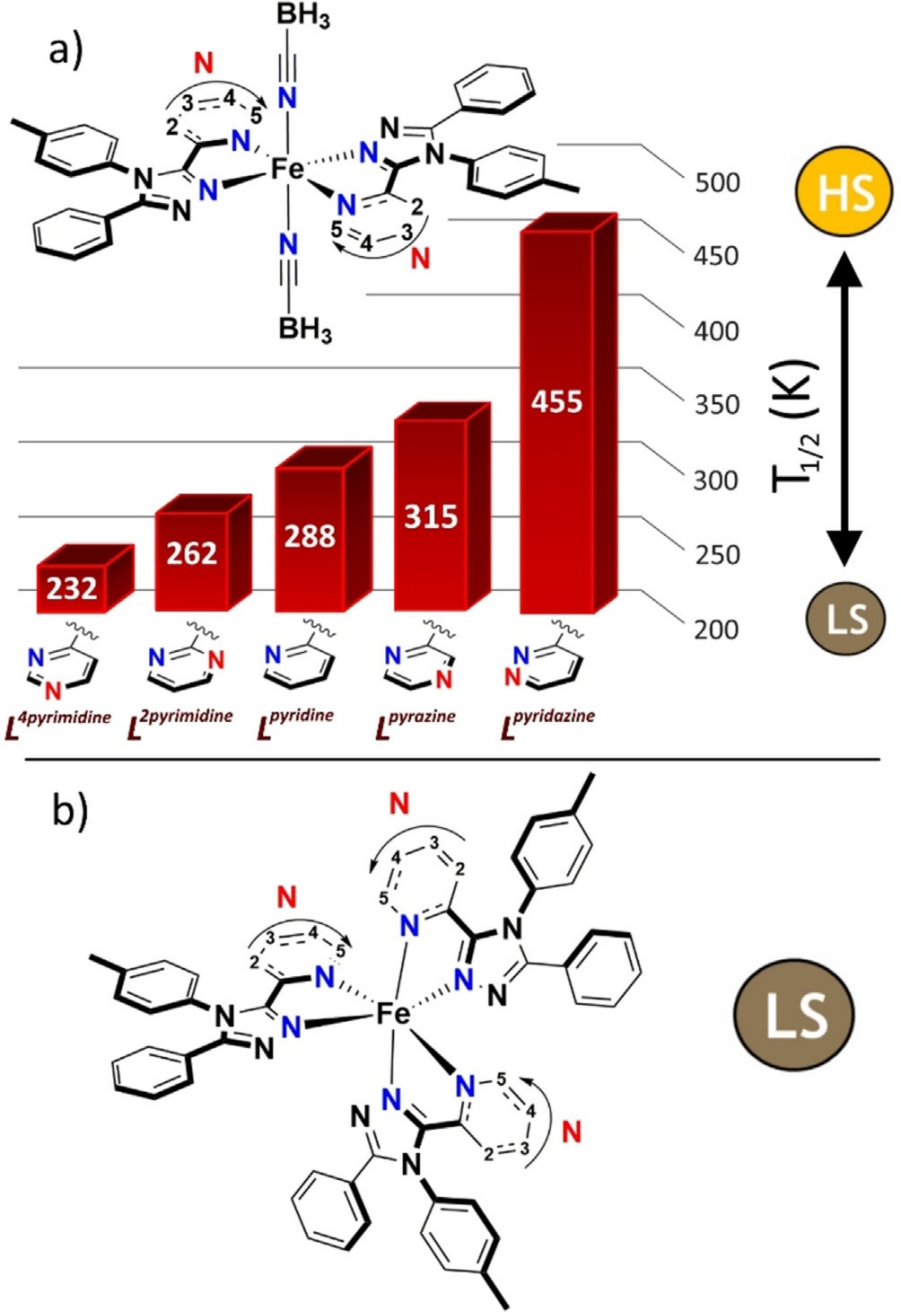
The two families of complexes studied here: a) five SCO‐active complexes, [Fe^II^(**L^azine^**)_2_(NCBH_3_)_2_], shown in order of increasing *T*_1/2_ in CDCl_3_ solution as a function of the azine, that is, position of the uncoordinated **N** (red): absent (**L^pyridine^**); or present in the 2‐position (**L^2pyrimidine^**), 3‐position (**L^pyrazine^**), 4‐position (**L^4pyrimidine^**), or 5‐position (**L^pyridazine^**)^[5k]^ and b) five LS [Fe^II^(**L^azine^**)_3_](BF_4_)_2_ complexes.^[16]^

Spin crossover (SCO)‐active complexes^[15]^ provide a very sensitive experimental probe of subtle changes in **M−L** bonds as **L** is modified, as the transition temperature (*T*_1/2_) at which the complex switches between the low‐spin (LS) and high‐spin (HS) states in solution is sensitive to these changes.[^5b^, ^5d^, ^5k^, ^6c^] Hence, a family of five [Fe^II^(***L*** ^***azine***^)_2_(NCBH_3_)_2_] complexes that vary in the choice of the azine ring (Figure 1), for which a linear correlation of the *T*_1/2_ with the ^15^N NMR chemical shift of the coordinating azine nitrogen atom in the respective ligand,^[5k]^ was chosen as the test system to trial this new approach to improving our detailed understanding of **M−L** interactions in octahedral complexes.^[7]^

Application of the resulting new protocol to this family of SCO‐active complexes then enabled us to evaluate the changes in the bonding properties across the family, obtained by EDA‐NOCV calculations,^[9]^ such as the σ‐donor and π‐acceptor character of the respective ligands, against the trend in the observed *T*_1/2_ values of the complexes. Doing this enabled us to determine whether or not the theoretical findings are consistent with experiment, and hence provide quantitative and chemically intuitive insights into the nature of the **M−L** bonds under consideration.

Finally, the optimized EDA‐NOCV protocol developed for the SCO‐active [Fe^II^(***L*** ^***azine***^)_2_(NCBH_3_)_2_] complexes was then used for the closely related [Fe^II^(***L*** ^***azine***^)_3_](BF_4_)_2_ family of LS complexes,^[16]^ where our calculations showed that three ***L*** ^***azine***^ ligands produce a stronger octahedral ligand field than a combination of 2 ***L*** ^***azine***^+2 NCBH_3_, which is in line with experimental findings.

Overall, this study has established and validated a generally applicable fragmentation and computational protocol for EDA‐NOCV **M−L** bonding analysis of any diamagnetic or paramagnetic, homoleptic or heteroleptic, octahedral transition metal complex.

## Computational Details

**Geometry optimization**: As a first step, accurate structures for these five [Fe^II^(***L*** ^***azine***^)_2_(NCBH_3_)_2_] complexes in both the LS and HS states are required, so density functional theory structure optimizations of the complexes were performed with the ORCA 4.1 software package.^[17]^ After testing several computational features (details in Section S1.1, Tables S1–S3 and Figures S2–S9 in the Supporting Information), the level of theory with the best overall performance was identified to be RI‐BP86‐D3(BJ)/def2‐TZVPP+CPCM(CHCl_3_).^[18]^ That is, usage of the BP86 functional[^18e^, ^18f^] together with the resolution of identity (RI) approximation,[^18h^, ^18i^] Grimme's D3 dispersion correction (including BJ damping),[^18a^, ^18b^] a def2‐TZVPP basis set^[18c]^ and implicit CPCM‐solvent model.^[18g]^ Using this protocol all of the calculated structures, for both the LS and HS complexes, are in good agreement with the available experimental X‐ray crystallographic data for the LS and HS states of the [Fe^II^(***L*** ^***pyridine***^)_2_(NCBH_3_)_2_] complex^[19]^ (Table S3). The [Fe^II^(***L*** ^***azine***^)_3_](BF_4_)_2_ complexes had been previously optimized by using the same protocol.^[16]^ These sets of optimized structures were then used in single‐point calculations for the subsequent EDA‐NOCV analyses performed using the ADF program package (version 2018.106; please note that the ADF version used does not allow the inclusion of solvent effects when performing EDA‐NOCV) at the BP86‐D3(BJ)/TZ2P level of theory.^[20]^

**Introduction to EDA‐NOCV**: The EDA‐NOCV^[9]^ method combines the classical EDA (Energy Decomposition Analysis), developed by Ziegler and Rauk,[^4b^, ^21^] with the natural orbitals for chemical valence (NOCV) extension, developed by Mitoraj and Michalak.^[8]^ As implemented in the 2009 release of the ADF program package,^[20b]^ it can be employed to quantify the bonding interactions in the complexes between the metal **M** and the surrounding ligands **L** in a chemically intuitive manner. To do so, EDA‐NOCV^[9]^ requires the complex to be split into two (or more) fragments, and the intrinsic, instantaneous interaction (relative stabilisation) energy Δ*E*_int_ of the **M−L** bonds formed between the two (or more) fragments in the frozen (unrelaxed) geometry of the molecule is then assessed.^[22]^ This total interaction energy, Δ*E*_int_, is comprised of four main contributions [Eq. 1]:

(1) $$\Delta E{}_{\mathrm{int}}=\Delta E{}_{\mathrm{elstat}}+\Delta E{}_{\mathrm{Pauli}}+\Delta E{}_{\mathrm{orb}}+\Delta E{}_{\mathrm{disp}}$$

The electrostatic interaction (Δ*E*_elstat_) is usually attractive (negative). It is computed quasi‐classically as the interaction between the unperturbed charge distributions of the atoms of the fragments. The Pauli repulsion (Δ*E*_Pauli_) comes from the energy increase arising from the required transformation from the superposition of the unperturbed electron densities of the isolated fragments to the proper, antisymmetrized and normalized wavefunction in the resulting bond, so is the only positive term in Equation (1). The orbital interaction term [Δ*E*_orb_; see also Eq. (2), below] is negative and accounts for the electron density distortion associated with the electron flow between 1) two different fragments to give the individual orbital contributions to the σ, π and δ bonds formed (Δ*E*_orb,i_, i=σ, π, δ) and 2) two regions of the same fragment to give the polarization term (Δ*E*_orb,pol_). The dispersion term (Δ*E*_disp_) is an extra contribution obtained from the explicit calculation of dispersive interactions,[^18a^, ^18b^] and is usually rather small and negative.

Comparison of Δ*E*_elstat_ and Δ*E*_orb_ can be used^[23]^ as a probe for determining the ratio between electrostatic (ionic) and covalent contributions to bonding between fragments.

Additionally, EDA‐NOCV is a charge decomposition method, since the Δ*E*_orb_ contribution to Δ*E*_int_ is commonly further split up into five subcontributions [Eq. 2]:

(2) $$\Delta E{}_{\mathrm{orb}}=\Delta E{}_{\mathrm{orb},\sigma }+\Delta E{}_{\mathrm{orb},\pi }+\Delta E{}_{\mathrm{orb},\delta }+\Delta E{}_{\mathrm{orb},\mathrm{pol}}+\Delta E{}_{\mathrm{orb},\mathrm{rest}}$$

and the charge flow associated with interactions between fragments (Δ*E*_orb,i_; σ, π and δ bond formation) and within fragments (Δ*E*_orb,pol_), into these different components can be separated via deformation densities Δ*ρ*_i_. The NOCV Scheme provides pairwise energy contributions to Δ*E*_orb,i_[^9a^, ^24^] for each pair of interacting orbitals. By visual inspection of the deformation densities Δ*ρ*_i_ it is possible to identify the various interaction types leading to bond formation (σ, π and δ) and hence their contributions (Δ*E*_orb,σ_, Δ*E*_orb,π_ and Δ*E*_orb,δ_) to the total orbital interaction Δ*E*_orb_. Additionally, information about the magnitude of the charge flow is given by the corresponding eigenvalues.^[9]^

Of the terms that contribute to the overall Δ*E*_orb_ term, herein particular attention is focussed on the nine terms identified by using Hoffmann's theory as these are of key importance for describing bonding in transition metal complexes:^[25]^ a total of six σ‐type interactions (Δ*E*_orb,σ_) between the **M** AOs (d${}_{x{}^{2}-y{}^{2}}$ , d${}_{z{}^{2}}$ , p_*x*_, p_*y*_, p_*z*_ and s orbitals) and the MOs with the corresponding symmetry in the **L_6_** fragment, plus three π‐type interactions (Δ*E*_orb,π_) between the remaining M AOs (d_*xy*_, d_*xz*_, d_*yz*_ orbitals) and the **L_6_** MOs of appropriate symmetry.

**Development of a computational protocol for a physically meaningful and chemically intuitive fragmentation scheme for any octahedral transition metal complex**: Interpretation of EDA‐NOCV results is known to be highly dependent on the choice of fragmentation of the molecule.[^4b^, ^26^] Moreover, complexes involving 3d metal ions pose a special challenge as it is desirable to reflect physically meaningful orbital occupations and energies in both possible situations: the bound complex and the isolated fragments. In the latter case, oftentimes the best representation would be achieved with fractionally occupying the energetically lower‐lying 3d orbitals of the metal,^[27]^ while in the former the occupation of the appropriate antibonding molecular orbitals with d character at the metal centre is mandatory. To find a balance between meaningful reference states, chemically intuitive orbital occupations and computational feasibility, a series of systematic EDA‐NOCV calculations with various fragmentation schemes and additional computational protocols has been performed which is detailed in the following. The result of this rigorous study is a robust fragmentation protocol that will enable the application of EDA‐NOCV analysis to any monometallic octahedral complex, regardless of whether homo‐ or heteroleptic and dia‐ or paramagnetic.

**Figure 2:**
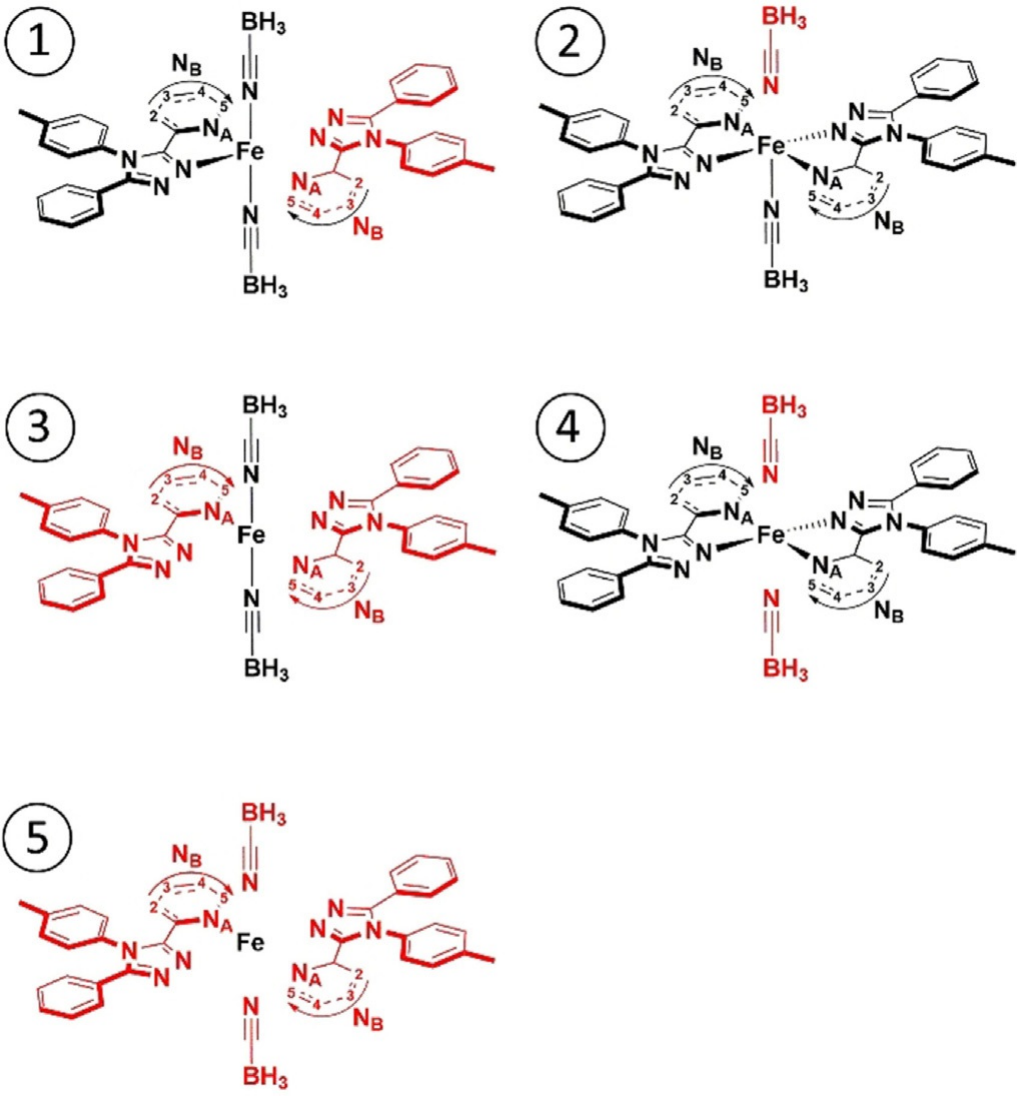
The five fragmentations **1**–**5** (top to bottom) trialled for EDA‐NOCV analysis of the five LS [Fe(**L^azine^**)_2_(NCBH_3_)_2_] complexes (fragment **1** in black; fragment **2** in red).

The family of five SCO‐active [Fe^II^(***L*** ^***azine***^)_2_(NCBH_3_)_2_] complexes comprise one metal ion (Fe^2+^), two constant axial anionic co‐ligands (NCBH_3_^−^) and two varying equatorial neutral bidentate ***L*** ^***azine***^ ligands. In the first step, a full test of five possible fragmentations that the LS [Fe^II^(***L*** ^***azine***^)_2_(NCBH_3_)_2_] complexes could be broken into (**1**–**5**, Figure 2) was carried out, as these being diamagnetic led to easier wavefunction convergence and clearer visual analysis of the NOCV results than for the analogous paramagnetic high‐spin state complexes. To our knowledge, a systematic study of fragmentation schemes, at the level presented here, is a novelty in the EDA‐NOCV‐based bonding analysis of transition metal complexes with d orbital configurations other than d^0^ and d^10^.[^13b^, ^13c^]

Fragmentations **1** and **2** (Figure 2) represent the most commonly used fragmentation types in the EDA‐NOCV literature when diamagnetic transition metal ions (LS d^6^ or d^10^) are present, removal of a single ligand.[^26a^, ^28^] Here either **L**=[NCBH_3_]^−^ (fragmentation **1**) or [***L*** ^***azine***^] (fragmentation **2**) is removed, so these provide detailed information on a single type of Fe−**L** interaction. However, the presence of another ligand of the same type in the other, iron‐containing, fragment makes these two fragmentation choices less than ideal here. Hence fragmentations **3** and **4** (Figure 2), in which a pair of identical ligands are removed, either both [2×**NCBH_3_** ^**−**^] (fragmentation **3**) or both [2×***L*** ^***azine***^] (fragmentation **4**) ligands, should provide a cleaner analysis of the details of the different types of Fe−**L** bonds. These fragmentation schemes are described in detail in Sections S3.1–S3.4. However, all four of these fragmentations, **1**–**4**, would only really be useful for examining trends within a family of very closely analogous complexes—confidently comparing very different coordination environments around **M** will be rather difficult, as the fragmentation is not general enough for that: The remaining metal‐bound ligands will surely affect the electronic environment of the metal ion so will subsequently influence the **M−L** bonding character.

In light of this, fragmentation **5** (Figure 2), in which all of the ligands are removed from the metal centre, is the most unbiased of all of these fragmentation options, and opens up the general application of the EDA‐NOCV analysis to any family of monometallic complexes. Whilst the Fe d orbital energies in fragmentations **1**–**4** are comparable to the frontier orbital energies of the ligands, as expected within Hoffman's MO diagram (Figure S1), this is not the case in fragmentation **5**. Due to the absence of partial ligand fields, which are induced by lone‐pair containing ligands surrounding the metal ion containing fragment in the other fragmentation schemes (**1**–**4**), the attractive potential of the Fe^2+^ centre is not “buffered” by electron density in the vicinity anymore and is therefore fully experienced by the d electrons.

So, although using Fe^2+^ instead of Fe^0^ as a fragment appears intuitive and convenient at first, the resulting Fe^2+^ d atomic energies for fragmentation **5 a** (no corrections, Section S2.9 and Table S4), are very low in energy (ca. −26.0 eV, see Tables 1 and S4), compared to the energies of the frontier orbitals of the ligands (between −4.0 and +4.0 eV, see Table S5).

**Table 1:** The calculated energy of the Fe(AO) frontier orbitals [eV] was used to establish the most appropriate way to deal with the very low energy observed for Fe^2+^ (ca. −26 eV) relative to Fe^0^ (ca. −8.0 eV) so that EDA‐NOCV analyses could be carried out for fragmentation **5** (**M**+**L_6_**) for the LS [Fe^II^(***L*** ^***azine***^)_2_(NCBH_3_)_2_] complexes. Note: the energy levels of the ligand frontier orbitals range from −4.0 to +4.0 eV.

Fe (O_h_)	T_2g_	E_g_	Δ*E* (E_g_−T_2g_)	Frag.
Fe^0^ (spherical sym.)	−7.93	−7.93	0.00	
Fe^2+^ (no charges)	−26.05	−25.61	0.56	**5 a**
Fe^2+^ (6×−0.425 *e*)	−8.00	−7.61	0.39	**5 b**
Fe^2+^ on Fe^0^ (AOs)	−7.96	−7.78	0.18	**5 e**

This strongly challenges the physical justification for this description of **M−L** bonding interactions because of the poor match in energies between interacting frontier orbitals. To overcome this dilemma, the free ion **M^n+^** of **5 a** was surrounded by varying amounts of negative charges in order to emulate the electron density of the ligand lone‐pairs (fragmentations **5 b**–**5 d**). A slightly different approach was taken with scheme **5 e**: Here the electron density of the isolated Fe^2+^ AOs was mapped onto the neutral Fe^0^ AOs. These approaches, **5 a**–**5 e**, are described in detail in Section S2.9, but are summarized as follows:

Fragmentation **5 a**: Fe^2+^

Fragmentation **5 b**: Fe^2+^+6×−0.425*e*

Fragmentation **5 c**: Fe^2+^+6×−1.0*e*

Fragmentation **5 d**: Fe^2+^+6×−2.0*e*

Fragmentation **5 e**: Fe^2+^ density mapped onto Fe^0^(AOs)

All treatments (**5 b–5 e**) effectively rescaled the Fe^2+^ d orbitals towards more positive energy levels (Tables 1 and S4). We found that through the computational protocols **5 b** and **5 e** the Fe^2+^ orbital energies were brought closest to the energy levels of Fe^0^ in spherical symmetry, and hence also to the ligand frontier orbital energies, which in turn yields a more chemically intuitive MO diagram for fragmentation into the isolated metal ion and the surrounding ligands with much better matching orbital energies.

Fragmentations **5 b** and **5 e** were found to have different advantages and disadvantages (vide infra; and see Sections S2.9 and S3.5 for more details) so both were applied for in depth analysis of the complexes depending on the quantity in question. Specifically, **5 b** allowed for identification of chemically intuitive bonding interactions by NOCV analysis but underestimated the Pauli repulsion (Δ*E*_Pauli_) in the EDA, whereas for **5 e** it is the other way around. Hence, we have employed fragmentation **5 e** to obtain information about the contributions to the intrinsic bond energy (Δ*E*_elstat_, Δ*E*_Pauli_, Δ*E*_orb_, Δ*E*_disp_) and—in separate calculations—fragmentation **5 b** to gain deeper insight into the orbital interactions and the relative contributions within by NOCV decomposition analysis.

As the purpose of this work is to provide a robust computational protocol to enable the application of EDA‐NOCV analysis to any monometallic complex, regardless of spin state or the exact nature of the coordination pocket provided by the coordinating ligands, a detailed description of the results of applying these two general fragmentations, **5 b/5 e** (i.e., corrected **M^n+^**+**L_6_**), in the EDA‐NOCV analysis of three families of complexes follows.

## Results and Discussion

As noted above, Δ*E*_elstat_ and Δ*E*_orb_ [Eq. (1)] are the EDA quantities that give indications of the ionic and covalent character of the chemical bonds formed between the two fragments (**5 e**; **M^n+^**+**L_6_**).

The term (fragmentation **5 b**; **M^n+^**+**L_6_**) that is expected to be most sensitive to the differences in the **M−L^azine^** bonds (due to the 5 different azines), and hence reflects the changes in the SCO properties, is Δ*E*_orb_ [Eq. (1)], in particular the σ and π contributions that involve the metal ion [Eq. (2); Δ*E*_orb,σ_ and Δ*E*_orb,π_]. Visual representations of all the σ and π contributions to the **M−L** bonding are provided by the NOCV deformation densities Δ*ρ*_**(i)**_ for each of the fragmentations employed. It should be noted that the general appearance is the same for the other four complexes in the respective family (treated with the same fragmentation), regardless of the different ***L*** ^***azine***^ ligands.

**Figure 3:**
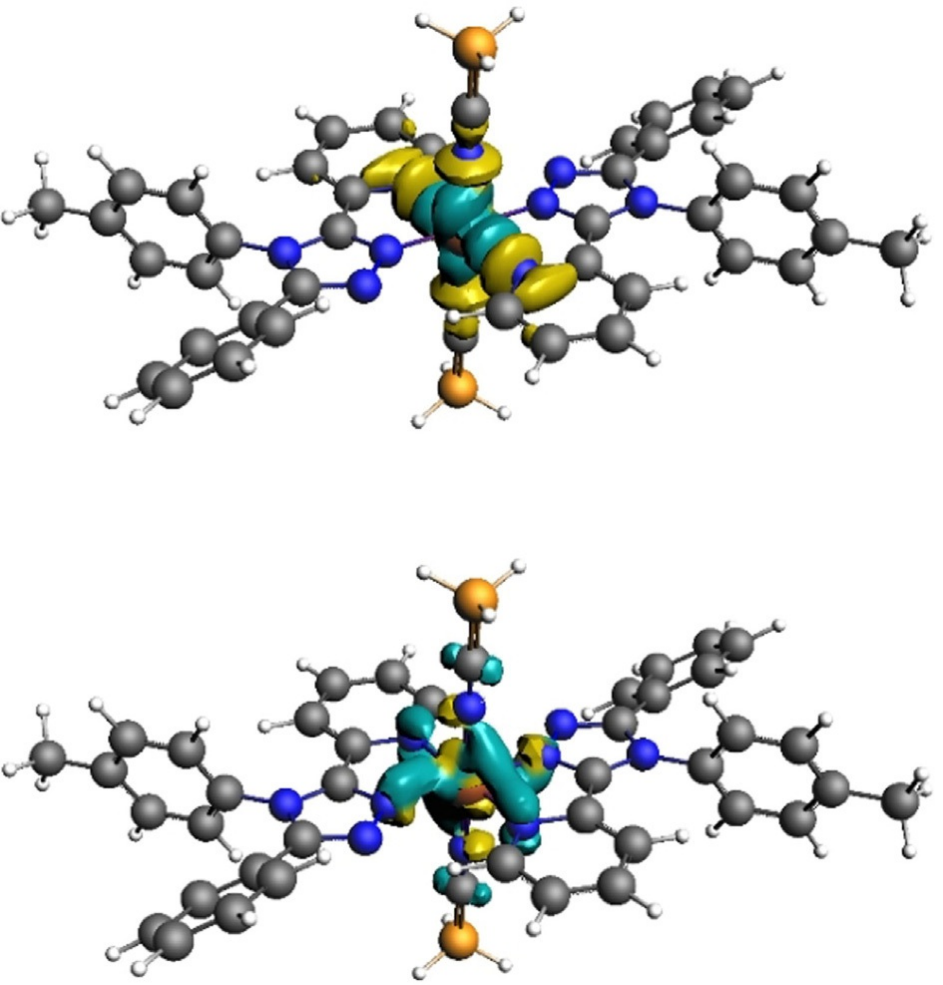
Plot of the deformation densities Δ*ρ*_(i)_ obtained for fragmentation **5 b** EDA‐NOCV analysis of LS Fe(***L*** ^***pyridine***^)_2_(NCBH_3_)_2_. These correspond to top: Δ*ρ*_2_, Fe(d${}_{x{}^{2}-y{}^{2}}$ )←ligand σ donation and bottom: Δ*ρ*_4_, Fe(d_*zx*_)→ligand π back donation. Direction of charge flow: yellow→turquoise. Cut‐off employed, Δ*ρ*_(i)_=0.003, produced the clearest image (see Figure S27 for more details).

These key parameters are presented for both the LS (Figures 3 and S27) and HS (Figure S28) state families of [Fe(***L*** ^***pyridine***^)_2_(NCBH_3_)_2_] and for the LS family of [Fe(***L*** ^***pyridine***^)_3_]^2+^ (see below, Figures 7 and S30).

### LS [Fe^II^(L^azine^)_2_(NCBH_3_)_2_]

As expected, due to the charged nature of the NCBH_3_^−^ co‐ligand, EDA using fragmentation **5 e** reveals that the bonding interaction is mainly ionic (Δ*E*_elstat_:Δ*E*_orb_=55:45; Figure 4).

**Figure 4:**
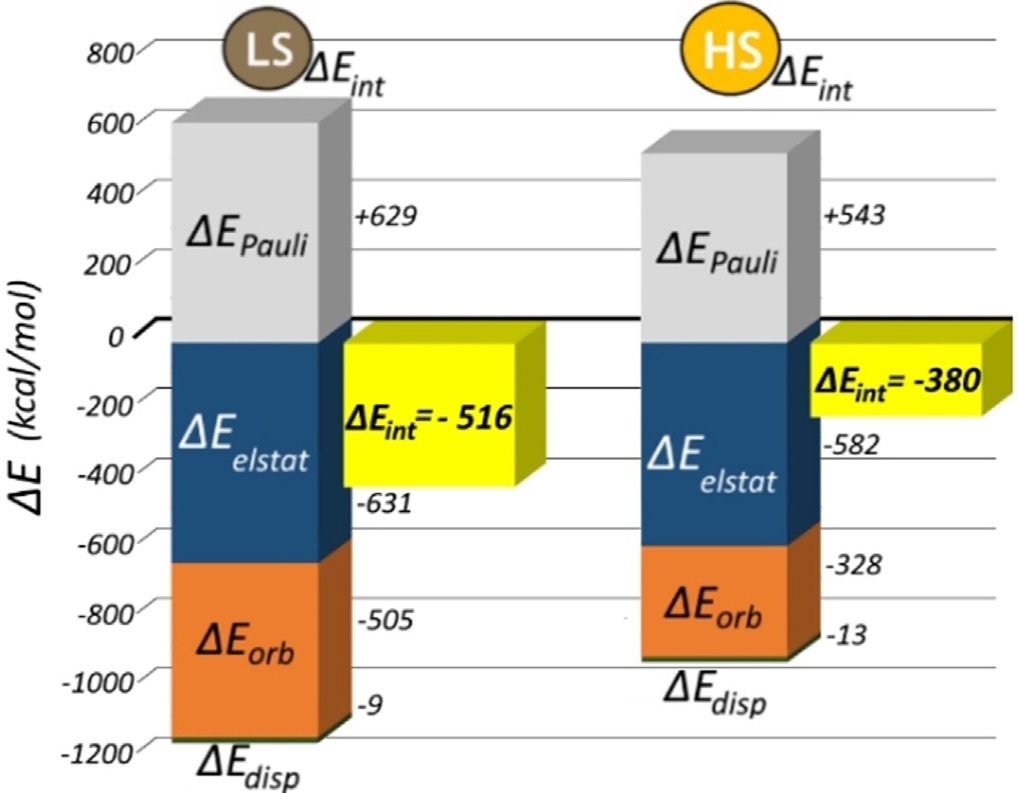
Results of EDA for LS versus HS [Fe(***L*** ^***pyridine***^)_2_ (NCBH_3_)_2_] using fragmentation **5 e**. For each spin state, the pair of bar graphs shows the four components of Δ*E*_int_ [Eq. (1); only Δ*E*_Pauli_ is positive] and their sum (Δ*E*_int_, yellow). Energies are in kcal mol^−1^.

**Figure 5:**
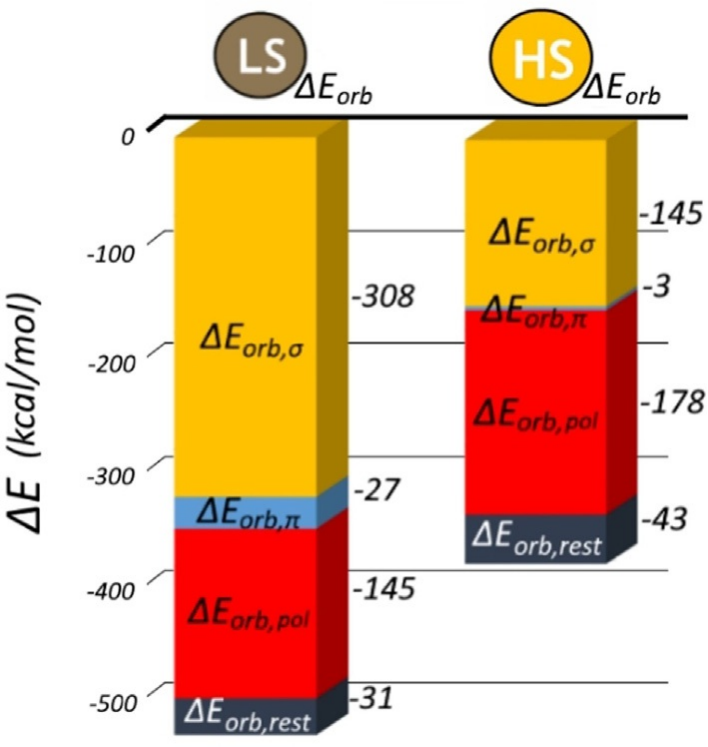
Results of NOCV decomposition of Δ*E*_orb_ for LS versus HS [Fe_2_(*L* ^*pyridine*^)_2_(NCBH_3_)] using fragmentation **5 b**. For each spin state, the bar graph shows the four components of Δ*E*_orb_ [Eq. (2)]. Energies are in kcal mol^−1^.

Furthermore, NOCV analysis using fragmentation **5 b** reveals the ratio of σ and π contributions to Δ*E*_orb_ is about 90:10 (Δ*E*_orb,σ_:Δ*E*_orb,π_; Figure 5, Table S21).

Focusing first on the **M←L** σ interactions, those involving the Fe^2+^ p and s orbitals provide a constant stabilization energy across the entire family (Table S21 and Figure S27). Hence, as expected, the variation in Δ*E*_orb,σ_ as the ***L*** ^***azine***^ changes from ***L*** ^***4pyrimidine***^ to ***L*** ^***pyrazine***^ is due to changes in the σ interactions formed by the Fe^2+^ d${}_{z{}^{2}}$ and d${}_{x{}^{2}-y{}^{2}}$ orbitals (Δ*ρ*_1_ and Δ*ρ*_2_, Figures 3 and S27). Unsurprisingly, these Δ*E*_orb,σ_ values do not fit the experimental observations (order of *T*_1/2_ values). The ***L*** ^***pyridazine***^ complex shows significantly smaller d${}_{z{}^{2}}$ (−102 kcal mol^−1^) and d${}_{x{}^{2}-y{}^{2}}$ (−110 kcal mol^−1^) orbital interactions than are seen in the other complexes (−113 to −114, and −116 to −119 kcal mol^−1^, respectively; Figure S27, Table S21).

Focusing next on the analysis of the three **M→L** π‐back‐donation contributions, Δ*E*_orb,π_, reveals: Δ*ρ*_3_ is mainly associated with the interaction of **M** with the *diazine* ring in the *yz* plane (Δ*E*_orb,3_ about −1 to −30 kcal mol^−1^ across the family); while Δ*ρ*_4_ is mainly associated with the interaction of **M** with the triazole ring in the *xz* plane (Δ*E*_orb,*4*_ constant at −11 kcal mol^−1^ across the family). Δ*ρ*_5_ lies in the *L* ^*azine*^ plane (*xy*) so both the diazine ring and the triazole ring of each ***L*** ^***azine***^ ligand participates in this bond (Δ*E*_orb,5_ constant at −15 kcal mol^−1^ across the family; Figure S27, Table S21). As for the Δ*E*_orb,σ_ values, the Δ*E*_orb,π_ values do not parallel the order of *T*_1/2_ values: again the ***L*** ^***pyridazine***^ complex is the outlier, with a significantly bigger Δ*E*_orb,3_ (−30 kcal mol^−1^) than the rest (−1 to −4 kcal mol^−1^).

### HS [Fe^II^(L^azine^)_2_(NCBH_3_)_2_]

**Figure 6:**
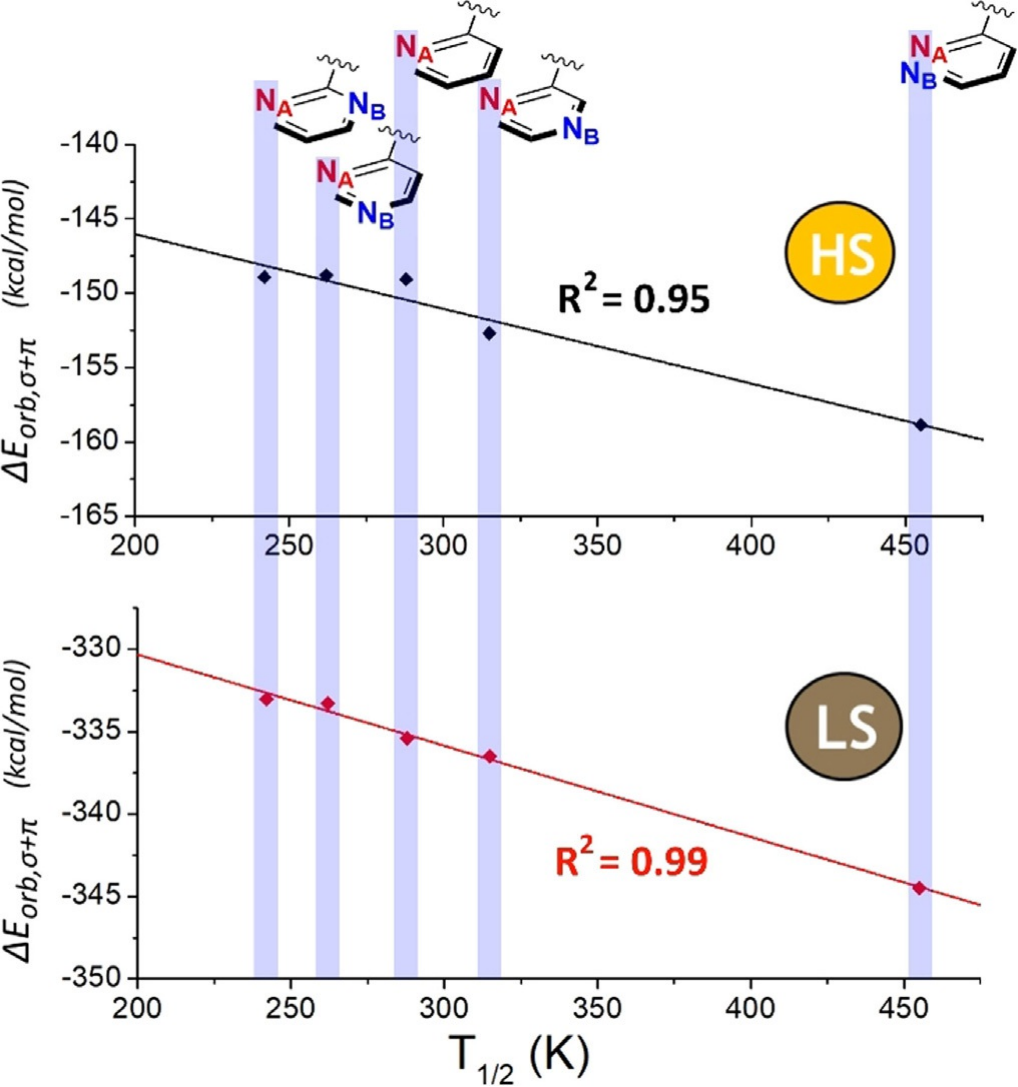
Strong correlations are seen between Δ*E*_orb,σ+π_ (calculated from fragmentation **5 b**) and *T*_1/2_, for both the LS‐state complexes (*R* ^2^=0.99) and the HS‐state complexes (*R* ^2^=0.95), but there is no correlation between the difference, Δ_LS‐HS_Δ*E*_orb,σ+π_, and *T*_1/2_ (*R* ^2^=0.12; Figure S29).

Moving to the HS family of [Fe(***L*** ^***azine***^)_2_(NCBH_3_)_2_] complexes (again using fragmentations **5 b** and **5 e**, Sections S2.9 and S3.6), unsurprisingly, the change in Fe^II^ spin state dramatically affects the **M−L** interactions. The EDA (fragmentation **5 e**) shows that on going from LS to HS the Δ*E*_int_ stabilization for the [Fe(***L*** ^***azine***^)_2_(NCBH_3_)_2_] family (Table S22) decreases by ca. 25 %, from about −500 to −370 kcal mol^−1^. The exact values depend on the ***L*** ^***azine***^ present; those for ***L*** ^***pyridine***^ are shown in Figure 4. This is consistent with the HS state being less stable enthalpically than the LS state, as expected as the HS state only becomes more stable than the LS state at higher temperatures when the entropic contributions become large enough to outweigh the enthalpic term. The three main contributions to Δ*E*_int_ [Δ*E*_orb_, Δ*E*_Pauli_ and Δ*E*_elstat_; Eq. (1), Figure 4, Table S22] are also reduced in magnitude when changing from LS to HS. Of them, the largest reduction is observed for Δ*E*_orb_ (from about −500 to about −330 kcal mol^−1^). In addition, the Δ*E*_orb_:Δ*E*_elstat_ ratio goes from 44:55 for LS to 35:63 for HS, values consistent with the HS state being less covalent and more ionic than the LS state. This quantitative analysis confirms the significant change in the nature of the **M−L** interactions that is anticipated on change of spin state. More details of the changes in **M−L** bonding on changing spin state are revealed by comparison of the results of the NOCV analysis (fragmentation **5 b**) for both spin states (Figures 5, 6 and S28, Table S22).

The Δ*E*_orb,σ+π_ for LS [Fe(***L*** ^***azine***^)_2_(NCBH_3_)_2_] lies between −330 and −350 kcal mol^−1^ and almost two‐thirds of this orbital interaction is provided by Δ*E*_orb,σ_, in particular by the formation of **M−L** σ bonds involving the **M** d${}_{z{}^{2}}$ and d${}_{x{}^{2}-y{}^{2}}$ (unoccupied) orbitals (Δ*E*_orb,σ_>100 kcal mol^−1^ each). In contrast, in HS [Fe(***L*** ^***azine***^)_2_(NCBH_3_)_2_] these two orbitals are now half‐occupied so **M−L** antibonding interactions are also present, dropping the Δ*E*_orb,σ_ stabilization energy values to less than −35 kcal mol^−1^ each; consequently, the total Δ*E*_orb,σ+π_ stabilization energy drops to between −145 and −160 kcal mol^−1^ in the HS state (Figure 6). As for the LS analogues, a constant contribution to Δ*E*_orb,σ_, almost unaffected by the spin state, is observed for the contributions where s and p of Fe^2+^ are involved, that is, Δ*E*_orb,σ_(s,p_*x*_,p_*y*_,p_*z*_) (Figure S28, Table S22).

Whilst the π contributions (Δ*E*_orb,π_) to Δ*E*_orb,σ+π_ are small in both spin states (Figure 5; LS −27 kcal mol^−1^ vs. HS −3 kcal mol^−1^), those involving the t_2g_ orbitals donating electron density back to the ligands show a large reduction in magnitude of stabilization on going from LS to HS (Figure S28, |*v_24_*|_α_) due to the lower number of electrons present in them.

In contrast, the fragment polarization contributions (Δ*E*_orb,pol_) provide greater stabilization in the HS state, by about −30 kcal mol^−1^ (Figure 5), regardless of ***L*** ^***azine***^.

In a nutshell, as expected by the occupation of antibonding orbitals, spin state switching from LS to HS (Figures 4, 5 and 6) greatly reduces the orbital contributions (Δ*E*_orb_) between **M** and **L_6_**, by ca 50 %, while the electrostatic interactions (Δ*E*_elstat_) only drop by =10 %, reflecting the reduction in the hardness of the metal ion as the radius increases (from 0.75 Å LS to 0.95 Å HS).^[29]^ This is consistent with the classical view, that on switching from LS to HS the **M−L** bond becomes more ionic and less covalent, with longer and weaker bonds due to decreases in both the σ and π interactions.

### Correlation of EDA‐NOCV parameters with *T*_1/2_

Given the above, the Δ*E*_orb,σ+π_ values obtained from the EDA‐NOCV analysis were expected to correlate with the ligand field strength of the bonds formed between the fragments **M^n+^** and **L_6_**. This is a useful test of whether or not this approach can provide a useful, general, quantitative and predictive tool for predicting *T*_1/2_ for an SCO system.

A very good correlation (R^2^=0.95) between the EDA‐NOCV calculated Δ*E*_orb,σ+π_ and the experimentally observed *T*_1/2_ is observed, regardless of whether the family of LS and HS state complexes is examined (Figure 6 and S29). This indicates that the new computational protocol is pleasingly sensitive, which is quite remarkable given that computed EDA‐NOCV Δ*E*_orb,σ+π_ values for ***L*** ^***4pyrimidine***^, ***L*** ^***2pyrimidine***^, ***L*** ^***pyridine***^ in particular lie within fractions of kcal mol^−1^ of each other. No correlation between *T*_1/2_ and the small difference between the Δ*E*_orb,σ+π_ values for the LS and HS states (Δ_LS‐HS_Δ*E*_orb,σ+π_) is observed (*R* ^2^=0.12, Figure S29). Rather, the single spin state trend (LS is the easier of the two to calculate) should be used, as it appears to be a good predictive tool. In summary, it is evident from these results that the change of ***L*** ^***azine***^ induces different alterations in the σ and π interactions, which only correlate (extremely well) with the *T*_1/2_ values when the synergy of the two contributions (Δ*E*_orb,σ+π_) is considered (Figure 6). The results also confirm the expected extreme difficulty in foreseeing the effect of a ligand on the *T*_1/2_ of a complex on the basis of simple consideration of σ or π contributions.

### LS [Fe^II^(L^azine^)_3_]^2+^

**Figure 7:**
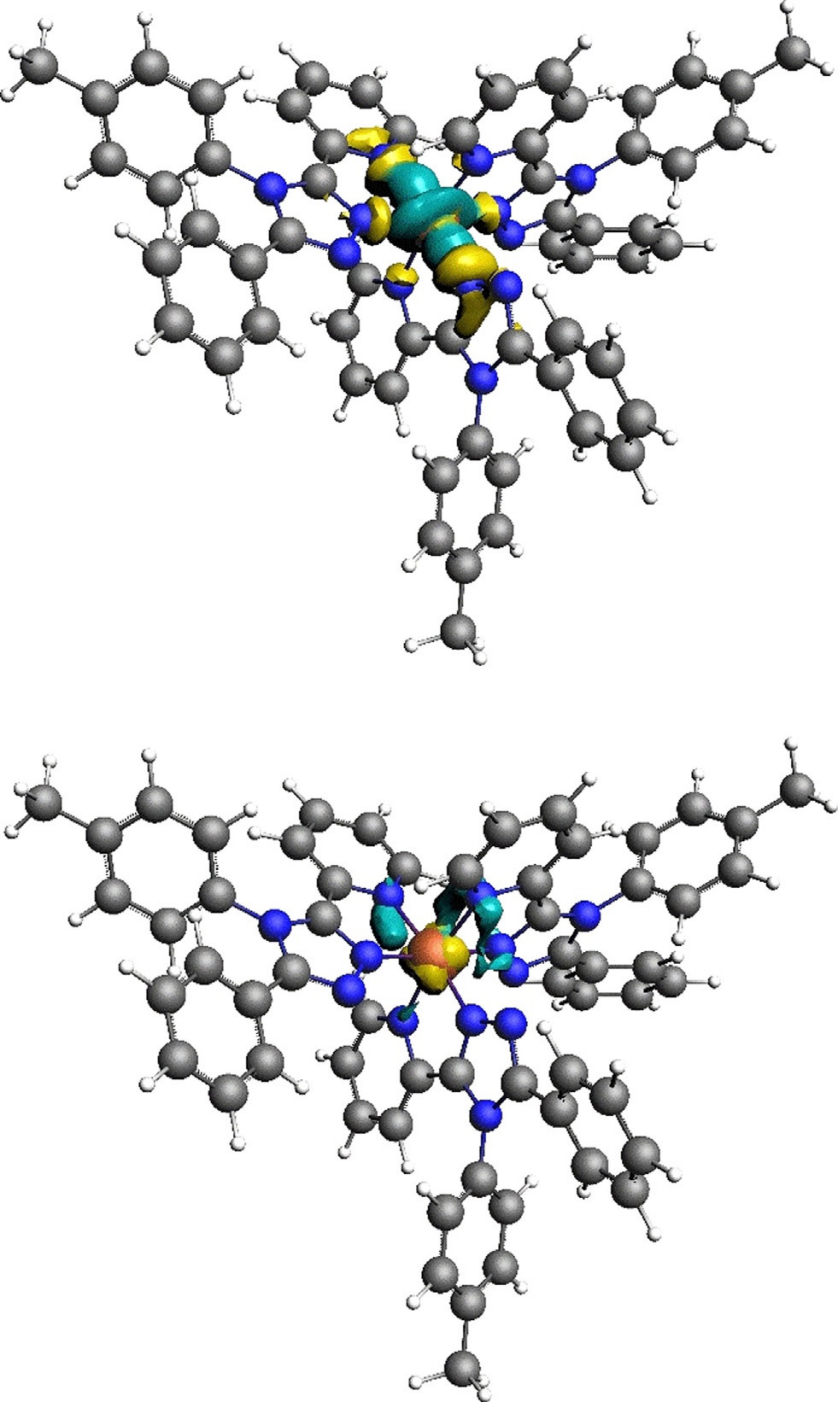
Plot of the deformation densities Δ*ρ*_(i)_ in fragmentation **5 b** (**M**+**L_6_**) of the [Fe(d${}_{z{}^{2}}$ )]←ligand σ donation (left) and the [Fe(d_*xz*_)] ligand π donation in reference complex LS [Fe(***L*** ^***pyridine***^)_3_^2+^]. The direction of the charge flow is yellow→turquoise. The eigenvalues |*v_i_*| indicate the relative size of the charge flow. Cut‐off employed, Δ*ρ*_(i)_=0.003, produced the clearest image (see Figure S30 for more details).

Application EDA‐NOCV (**M^n+^**+**L_6_**; based on **5 b/5 e**) to the closely related family of LS [Fe(***L*** ^***azine***^)_3_]^2+^ complexes (Figure 7) provided a new set of charged candidates to start to test the generality of these protocols. For the LS [Fe(***L*** ^***azine***^)_3_]^2+^ family the EDA revealed a Δ*E*_elstat_:Δ*E*_orb_ ratio of about 45:55 (Figure S30, Table S23), revealing greater covalent than ionic bonding, in contrast to the LS [Fe(***L*** ^***azine***^)_2_(NCBH_3_)_2_] complexes in which this ratio is reversed (Δ*E*_elstat_:Δ*E*_orb_=55:45) (Table S23).

This is not surprising as in the present case none of the ligands are charged, whereas in the [Fe(***L*** ^***azine***^)_2_(NCBH_3_)_2_] complexes two anions are involved. This results, when going from [Fe(***L*** ^***azine***^)_2_(NCBH_3_)_2_] to [Fe(***L*** ^***azine***^)_3_]^2+^ (Table S23), in a large decrease in Δ*E*_elstat_ stabilization (ca. −620 to −400 kcal mol^−1^) and a slight increase in Δ*E*_orb_ stabilization (=−15 to −20 kcal mol^−1^). The same magnitude of increase in stability observed for the Δ*E*_orb_ term is observed as an increase in Δ*E*_Pauli_ stabilization (=+15 to +20 kcal mol^−1^). This is consistent with the general trend that these two terms, Δ*E*_orb_ and Δ*E*_Pauli_, are intimately connected in describing the covalent bonding between fragments (Table S23). The NOCV analysis reveals that on stepping across the five ***L*** ^***azine***^ ligand from ***L*** ^***4pyrimidine***^ (weakest field strength, least negative Δ*E*_orb,σ+π_) *to **L*** ^***pyridazine***^ (strongest field strength, most negative Δ*E*_orb,σ+π_) that: 1) the σ bonds (Δ*E*_orb,σ_) involving the d${}_{z{}^{2}}$ and d${}_{x{}^{2}-y{}^{2}}$ orbitals strengthen by about −5 to −10 kcal mol^−1^ per bond per step and 2) the π backbonds (Δ*E*_orb,π_) involving the d_*xy*_, d_*zx*_, d_*zy*_ orbitals strengthen by about −5 to −15 kcal mol^−1^ per bond per step (Figures 7 and S30, Table S23).

On the other hand, as before, the bonds involving s, p_*x*_, p_*y*_ and p_*z*_ orbitals show marginal differences (Figure S23, Table S30).

Analysis of the σ and π contributions shows that the σ interaction is almost eight times larger than the π interaction regardless of ***L*** ^***azine***^. The σ strength (Δ*E*_orb,σ_) of the ***L*** ^***azine***^ ligands follows the order:

L^pyridazine^>L^4pyrimidine^>L^2pyrimidine^>L^pyrazine^>L^pyridine^.

Interestingly the order of the π strength (Δ*E*_orb,π_) of the ***L*** ^***azine***^ ligands differs (and the values are far from showing a monotonic trend):

L^pyridine^>L^pyrazine^>L^pyridazine^>L^2pyrimidine^>L^4pyrimidine^.

Adding those two contributions together gives Δ*E*_orb,σ+π_ and this puts the complexes into the same order as was observed experimentally for the [Fe(NCBH_3_)_2_(***L*** ^***azine***^)_2_] family, with an average magnitude decrease in Δ*E*_orb,σ+π_ stability of about 30 kcal mol^−1^ between LS [Fe(***L*** ^***azine***^)_3_]^2+^ and LS [Fe(***L*** ^***azine***^)_2_(NCBH_3_)_2_] (Table S21 and Table S23):

L^4pyrimidine^>L^2pyrimidine^>L^pyridine^>L^pyrazine^>L^pyridazine^.

### *L* ^*azine*^ versus 2x[NCBH_3_]^−^: ligand field strength comparison

**Figure 8:**
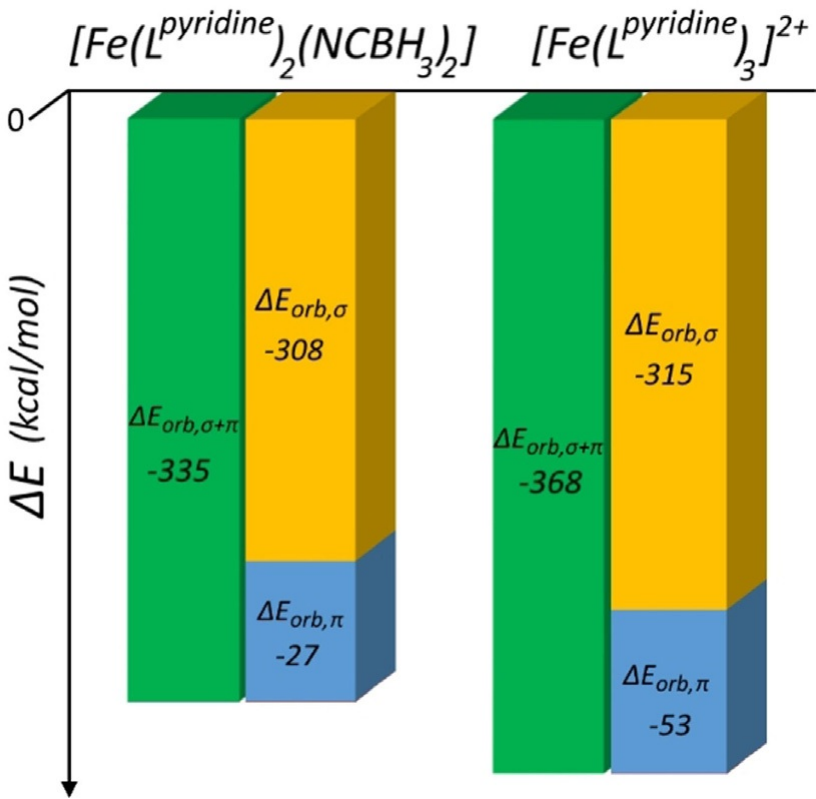
Comparison of Δ*E*_orb,σ+π_ (and components) calculated for LS [Fe(***L*** ^***pyridine***^)_2_(NCBH_3_)_2_] (left) and LS [Fe(***L*** ^***pyridine***^)_3_]^2+^ (right) using corrected **M+L_6_** (**5 b** for NOCV) is consistent with the former being SCO active and the latter remaining LS.

The above results enable another test of whether or not this EDA‐NOCV protocol (**M+L_6_**) can provide a useful, general, quantitative and predictive tool—in this case to compare the field strength of a pair of ligands in different types of complexes (Figure 8).

In contrast to the SCO‐active [Fe^II^(***L*** ^***azine***^)_2_(NCBH_3_)_2_] family,^[5k]^ the related family of [Fe^II^(***L*** ^***azine***^)_3_](BF_4_)_2_ complexes are all LS,^[16]^ which implies that the replacement of two NCBH_3_^−^ anions by one bidentate ***L*** ^***azine***^ ligand increases the ligand field experienced by the iron(II) centre. The Δ*E*_orb,σ+π_ values (Figure 8) for [Fe(***L*** ^***azine***^)_2_(NCBH_3_)_2_] (−335 kcal mol^−1^) and [Fe(***L*** ^***azine***^)_3_]^2+^ (−368 kcal mol^−1^) show that replacement of 2x**NCBH_3_** ^**−**^ by one ***L*** ^***azine***^ leads to an increase in the stabilization (ΔΔ*E*_orb,σ+π_) of −33 kcal mol^−1^ (Figure 8), which is consistent with the experimental observation that the [Fe(***L*** ^***azine***^)_2_(NCBH_3_)_2_] family are SCO‐active whereas the [Fe(***L*** ^***azine***^)_3_]^2+^ family are solely LS.

## Conclusions

In this study, we aimed to provide new insights into the details of the nature of **M−L** bonds. To do so, EDA‐NOCV was employed as it provides results that are both quantitative and chemically intuitive. This makes it a very powerful tool for both theoreticians and inorganic chemists. Hence, it was surprising to find that, prior to this study, the choice of fragmentation issue in EDA‐NOCV had not been rigorously developed to provide a general, widely applicable and consistent scheme for use in *any* 3d complex, regardless of whether the coordination was homoleptic or heteroleptic, or the complex was paramagnetic or diamagnetic.

Therefore, the first step was to consider a range of possible fragmentations of the complexes, starting from the usual literature fragmentation used (loss of one ligand). That, and the related fragmentations (loss of pairs of ligands) were found to be unsatisfactory, and also lacked generality, that is, the potential to be used for any complex regardless of ligand type or charge. Hence a protocol that enables robust and general EDA‐NOCV analysis of any octahedral coordination complex, fragmentation into **M^n+^+L_6_**, was developed. By keeping one fragment as a constant pure (unperturbed by the any ligand) metal ion **M^n+^**, any and all changes to the other, “all ligand”, fragment can then be analysed in depth and compared.

A family of SCO‐active Fe^II^ complexes, [Fe(***L*** ^***azine***^)_2_(NCBH_3_)_2_], was chosen as the test system for this study, as the experimentally observed solution switching temperatures (*T*_1/2_) provided the order of ***L*** ^***azine***^ ligand field strengths. Also, the chance to work on both spin states, diamagnetic LS and paramagnetic HS, enabled us to significantly increase the small handful of reports of EDA‐NOCV analysis of paramagnetic transition metal complexes^[11]^ and, above all, to critically tackle this class of system in depth for the first time. Moreover, this work is also the first to focus on EDA‐NOCV analysis of the complex electronic structures of SCO‐active systems, enabling in depth analysis and comparison of the **M−L** bonding in both of the thermodynamically accessible spin states, diamagnetic LS and paramagnetic HS.

Regardless of whether the LS or HS family of [Fe(***L*** ^***azine***^)_2_(NCBH_3_)_2_] complexes was examined by EDA‐NOCV, the analysis identified a good correlation (*R* ^2^: LS 0.99; HS 0.95) between decreasing *T*_1/2_ and increasing ligand field strength as quantified by the Δ*E*_orb,σ+π_ term. In addition, comparison of the results for [Fe(***L*** ^***azine***^)_2_(NCBH_3_)_2_] with those subsequently obtained on the LS [Fe(***L*** ^***azine***^)_3_](BF_4_)_2_ complexes revealed that only the corrected **M^n+^ + L_6_** fragmentation provides a general protocol suitable for comparing different types of complexes. It should be noted that the above analysis neglects any entropic contributions, which are known to be key in SCO, so the next big step in the development of this approach for applications in the SCO field will be understanding how the inclusion of computed entropic contributions can be included so that the unbiased determination of the *T*_1/2_ values on the basis of the EDA values will be possible.

In conclusion, the EDA‐NOCV protocol developed and validated herein employs a new and general fragmentation type (**M^n+^ + L_6_**) that provides a clear, quantitative and chemically intuitive description of the **M−L** bonds in these paramagnetic and diamagnetic transition metal complexes. This new protocol should be widely applicable, a point we are currently testing further (with more families of SCO and/or redox‐active 3d coordination complexes) in order to prove that it is general, thereby unlocking the great promise it holds as a predictive tool.

## Conflict of interest

The authors declare no conflict of interest.

## Supporting information

As a service to our authors and readers, this journal provides supporting information supplied by the authors. Such materials are peer reviewed and may be re‐organized for online delivery, but are not copy‐edited or typeset. Technical support issues arising from supporting information (other than missing files) should be addressed to the authors.

Supplementary

## References

[1a] L. C. Kuo , M. W. Makinen , J. Am. Chem. Soc. 1985, 107, 5255–5261;

[1b] K. P. Kepp , Coord. Chem. Rev. 2013, 257, 196–209.

[2a] K. E. Dalle , J. Warnan , J. J. Leung , B. Reuillard , I. S. Karmel , E. Reisner , Coord. Chem. Rev. 2019, 119, 2752–2875; 10.1021/acs.chemrev.8b00392 PMC6396143 30767519

[2b] A. G. Maher , G. Passard , D. K. Dogutan , R. L. Halbach , B. L. Anderson , C. J. Gagliardi , M. Taniguchi , J. S. Lindsey , D. G. Nocera , ACS Catal. 2017, 7, 3597–3606.

[3a] T. Glaser , V. Hoeke , K. Gieb , J. Schnack , C. Schröder , P. Müller , Coord. Chem. Rev. 2015, 289, 261–278;

[3b] S. Dhers , H. L. C. Feltham , S. Brooker , Coord. Chem. Rev. 2015, 296, 24–44.

[4a] L. W. Yarbrough , M. B. Hall , Inorg. Chem. 1978, 17, 2269–2275;

[4b] T. Ziegler , A. Rauk , Inorg. Chem. 1979, 18, 1755–1759;

[4c] G. Zhou , C. L. Ho , W. Y. Wong , Q. Wang , D. Ma , L. Wang , Z. Lin , T. B. Marder , A. Beeby , Adv. Funct. Mater. 2008, 18, 499–511.

[5a] K. Nakano , N. Suemura , K. Yoneda , S. Kawata , S. Kaizaki , Dalton Trans. 2005, 740–743; 15702186 10.1039/b416986g

[5b] L. J. Kershaw Cook , R. Kulmaczewski , R. Mohammed , S. Dudley , S. A. Barrett , M. A. Little , R. J. Deeth , M. A. Halcrow , Angew. Chem. Int. Ed. 2016, 55, 4327–4331; 10.1002/anie.201600165 PMC4804750 26929084

[5c] H. Phan , J. J. Hrudka , D. Igimbayeva , L. M. Lawson Daku , M. Shatruk , J. Am. Chem. Soc. 2017, 139, 6437–6447; 28402639 10.1021/jacs.7b02098

[5d] A. Kimura , T. Ishida , ACS Omega 2018, 3, 6737–6747; 31458846 10.1021/acsomega.8b01095 PMC6644749

[5e] K. P. Kepp , Inorg. Chem. 2016, 55, 2717–2727; 26913489 10.1021/acs.inorgchem.5b02371

[5f] A. Rudavskyi , C. Sousa , C. de Graaf , R. W. A. Havenith , R. Broer , J. Chem. Phys. 2014, 140, 184318; 24832281 10.1063/1.4875695

[5g] S. Ye , F. Neese , Inorg. Chem. 2010, 49, 772–774; 20050628 10.1021/ic902365a

[5h] E. C. Constable , P. J. Steel , Coord. Chem. Rev. 1989, 93, 205–223;

[5i] K. B. Wiberg , D. Nakaji , C. M. Breneman , J. Am. Chem. Soc. 1989, 111, 4178–4190;

[5j] J. A. Joule , K. Mills , G. F. Smith , Journal of Heterocyclic Chemistry , 3rd ed., Chapman & Hall, Padstow, 1995;

[5k] S. Rodríguez-Jiménez , M. Yang , I. Stewart , A. L. Garden , S. Brooker , J. Am. Chem. Soc. 2017, 139, 18392–18396. 29156884 10.1021/jacs.7b11069

[6a] I. Prat , A. Company , T. Corona , T. Parella , X. Ribas , M. Costas , Inorg. Chem. 2013, 52, 9229–9244; 23901826 10.1021/ic4004033

[6b] A. Kimura , T. Ishida , Inorganics 2017, 5, 52;

[6c] J. N. McPherson , R. W. Hogue , F. S. Akogun , L. Bondi , E. T. Luis , J. R. Price , A. L. Garden , S. Brooker , S. B. Colbran , Inorg. Chem. 2019, 58, 2218–2228. 30672281 10.1021/acs.inorgchem.8b03457

[7] I. B. Bersuker , Electronic Structure and Properties of Transition Metal Compounds: Introduction to the Theory, Wiley, New York, 2010.

[8a] M. Mitoraj , A. Michalak , J. Mol. Model 2008, 14, 681–687; 18278526 10.1007/s00894-008-0276-1

[8b] A. Michalak , M. Mitoraj , T. Ziegler , J. Phys. Chem. A 2008, 112, 1933–1939. 18266342 10.1021/jp075460u

[9a] S. Lin , C. S. Diercks , Y.-B. Zhang , N. Kornienko , E. M. Nichols , Y. Zhao , A. R. Paris , D. Kim , P. Yang , O. M. Yaghi , Science 2015, 349, 1208–1213; 26292706 10.1126/science.aac8343

[9b] G. Frenking , S. Shaik , The Chemical Bond: Chemical Bonding Across the Periodic Table, Vol. 2, Wiley, New York, 2014.

[10a] G. Frenking , L. Zhao , M. Zhou , S. Pan , G. Deng , S. Lei , G. Wang , J. Jin , Chem. Eur. J. 2020, 26, 10487–10500; 10.1002/chem.201905552 PMC7496348 32191361

[10b] A. J. Lupinetti , V. Jonas , W. Thiel , S. H. Strauss , G. Frenking , Chem. Eur. J. 1999, 5, 2573–2583;

[10c] Q. Wang , S. Pan , S. Lei , J. Jin , G. Deng , G. Wang , L. Zhao , M. Zhou , G. Frenking , Nat. Commun. 2019, 10, 1–8; 31358748 10.1038/s41467-019-11323-5 PMC6662891

[10d] Y. Kan , J. Mol. Struct. 2007, 805, 127–132.

[11a] N.-J. H. Kneusels , J. E. Münzer , K. Flosdorf , D. Jiang , B. Neumüller , L. Zhao , A. Eichhöfer , G. Frenking , I. Kuzu , Dalton Trans. 2020, 49, 2537–2546; 32022052 10.1039/c9dt04725e

[11b] L. S. Jeremias , J. Novotný , M. Repisky , S. Komorovsky , R. Marek , Inorg. Chem. 2018, 57, 8748–8759; 30004686 10.1021/acs.inorgchem.8b00073

[11c] M. von Hopffgarten , G. Frenking , WIREs Comput. Mol. Sci. 2012, 2, 43–62;

[11d] O. Lyubimova , O. V. Sizova , C. Loschen , G. Frenking , J. Mol. Struct. 2008, 865, 28–35;

[11e] C. H. Suresh , G. Frenking , Organometallics 2010, 29, 4766–4769;

[11f] H. Keypour , A. Shooshtari , M. Rezaeivala , M. Bayat , H. A. Rudbari , Inorg. Chim. Acta 2016, 440, 139–147;

[11g] M. Bayat , M. Hatami , Polyhedron 2016, 110, 46–54;

[11h] P. Pietrzyk , K. Podolska , T. Mazur , Z. Sojka , J. Am. Chem. Soc. 2011, 133, 19931–19943. 22035420 10.1021/ja208387q

[12a] W. A. Rabanal-León , J. A. Murillo-López , R. Arratia-Pérez , Phys. Chem. Chem. Phys. 2016, 18, 33218–33225; 27892559 10.1039/c6cp07001a

[12b] C. Chi , S. Pan , J. Jin , L. Meng , M. Luo , L. Zhao , M. Zhou , G. Frenking , Chem. Eur. J. 2019, 25, 11772–11784; 31276242 10.1002/chem.201902625 PMC6772027

[12c] J. Jin , S. Pan , X. Jin , S. Lei , L. Zhao , G. Frenking , M. Zhou , Chem. Eur. J. 2019, 25, 3229–3234. 30566753 10.1002/chem.201805260

[13a] W.-L. Li , Q. Zhang , M. Chen , H.-S. Hu , J. Li , M. Zhou , Angew. Chem. Int. Ed. 2020, 59, 4288–4293;

[13b] W. Yang , K. E. Krantz , L. A. Freeman , D. A. Dickie , A. Molino , G. Frenking , S. Pan , D. J. D. Wilson , R. J. Gilliard, Jr , Angew. Chem. Int. Ed. 2020, 59, 3850–3854; 10.1002/anie.201909627 PMC7064902 31816143

[13c] X. Wu , L. Zhao , J. Jin , S. Pan , W. Li , X. Jin , G. Wang , M. Zhou , G. Frenking , Science 2018, 361, 912–916; 30166489 10.1126/science.aau0839

[13d] X. Wu , L. Zhao , D. Jiang , I. Fernández , R. Berger , M. Zhou , G. Frenking , Angew. Chem. Int. Ed. 2018, 57, 3974–3980; 10.1002/anie.201713002 29431895

[13e] A. M. Priya , S. Lakshmipathi , Mol. Phys 2017, 115, 839–859.

[14] T. Stauch , R. Chakraborty , M. Head-Gordon , ChemPhysChem 2019, 20, 2742–2747. 31538686 10.1002/cphc.201900853 PMC6899727

[15a] R. Sieber , S. Decurtins , H. Stoeckli-Evans , C. Wilson , D. Yufit , J. A. K. Howard , S. C. Capelli , A. Hauser , Chem. Eur. J. 2000, 6, 361–368; 11931117 10.1002/(SICI)1521-3765(20000117)6:2<361::AID-CHEM361>3.0.CO;2-Y

[15b] P. Gütlich , A. Hauser , Compr. Coord. Chem. 2003, 2, 427–434;

[15c] P. Gütlich , H. A. Goodwin , Top. Curr. Chem. 2004, 233, 1–47;

[15d] J.-F. Létard , J. Mater. Chem. 2006, 16, 2550–2559;

[15e] K. Oka , M. Azuma , W.-t. Chen , H. Yusa , A. A. Belik , E. Takayama-Muromachi , M. Mizumaki , N. Ishimatsu , N. Hiraoka , M. Tsujimoto , M. G. Tucker , J. P. Attfield , Y. Shimakawa , J. Am. Chem. Soc. 2010, 132, 9438–9443; 20568754 10.1021/ja102987d

[15f] M. J. Murphy , K. A. Zenere , F. Ragon , P. D. Southon , C. J. Kepert , S. M. Neville , J. Am. Chem. Soc. 2017, 139, 1330–1335; 28045257 10.1021/jacs.6b12465

[15g] G. Chastanet , C. Desplanches , C. Baldé , P. Rosa , M. Marchivie , P. Guionneau , Chem. Sq. 2018, 2, 2.

[16] S. Rodríguez-Jiménez , L. Bondì , M. Yang , A. L. Garden , S. Brooker , Chem. Asian J. 2019, 14, 1158–1166. 30550630 10.1002/asia.201801537

[17] F. Neese , WIREs Comput. Mol. Sci. 2018, 8, e1327.

[18a] S. Grimme , S. Ehrlich , L. Goerigk , J. Comput. Chem. 2011, 32, 1456–1465; 21370243 10.1002/jcc.21759

[18b] S. Grimme , J. Antony , S. Ehrlich , H. Krieg , J. Chem. Phys. 2010, 132, 154104; 20423165 10.1063/1.3382344

[18c] F. Weigend , R. Ahlrichs , Phys. Chem. Chem. Phys. 2005, 7, 3297–3305; 16240044 10.1039/b508541a

[18d] F. Weigend , Phys. Chem. Chem. Phys. 2006, 8, 1057–1065; 16633586 10.1039/b515623h

[18e] A. D. Becke , Phys. Rev. A 1988, 38, 3098–3100; 10.1103/physreva.38.3098 9900728

[18f] J. P. Perdew , W. Yue , Phys. Rev. B 1986, 33, 8800–8802; 10.1103/physrevb.33.8800 9938293

[18g] Y. Takano , K. N. Houk , J. Chem. Theory Comput. 2005, 1, 70–77; 26641117 10.1021/ct049977a

[18h] O. Vahtras , J. Almlöf , M. W. Feyereisen , Chem. Phys. Lett. 1993, 213, 514–518;

[18i] F. Weigend , M. Häser , Theor. Chem. Acc. 1997, 97, 331–340.

[19] S. Rodríguez-Jiménez , S. Brooker , Inorg. Chem. 2017, 56, 13697–13708. 29112392 10.1021/acs.inorgchem.7b01338

[20a] E. Van Lenthe , E. J. Baerends , J. Comput. Chem. 2003, 24, 1142–1156; 12759913 10.1002/jcc.10255

[20b] G. T. Te Velde , F. M. Bickelhaupt , E. J. Baerends , C. Fonseca Guerra , S. J. van Gisbergen , J. G. Snijders , T. Ziegler , J. Comput. Chem. 2001, 22, 931–967.

[21] T. Ziegler , A. Rauk , Inorg. Chem. 1979, 18, 1558–1565.

[22] F. M. Bickelhaupt , E. J. Baerends , Rev. Comput. Chem. 2000, 15, 1–86.

[23] G. Frenking , J. Organomet. Chem. 2001, 635, 9–23.

[24a] M. P. Mitoraj , A. Michalak , T. Ziegler , J. Chem. Theory Comput. 2009, 5, 962–975; 26609605 10.1021/ct800503d

[24b] M. Mitoraj , A. Michalak , J. Mol. Model. 2007, 13, 347–355. 17024408 10.1007/s00894-006-0149-4

[25] T. A. Albright , J. K. Burdett , M.-H. Whangbo , Orbital Interactions in Chemistry, Wiley, New York, 2013.

[26a] P. Jerabek , H. W. Roesky , G. Bertrand , G. Frenking , J. Am. Chem. Soc. 2014, 136, 17123–17135; 25394669 10.1021/ja508887s

[26b] P. Jerabek , P. Schwerdtfeger , G. Frenking , J. Comput. Chem. 2019, 40, 247–264; 30365176 10.1002/jcc.25584

[26c] M. P. Mitoraj , M. Parafiniuk , M. Srebro , M. Handzlik , A. Buczek , A. Michalak , J. Mol. Model. 2011, 17, 2337; 21445707 10.1007/s00894-011-1023-6

[26d] L. Zhao , S. Pan , N. Holzmann , P. Schwerdtfeger , G. Frenking , Chem. Rev. 2019, 119, 8781–8845; 31251603 10.1021/acs.chemrev.8b00722

[26e] D. Munz , Organometallics 2018, 37, 275–289.

[27] E. Baerends , V. Branchadell , M. Sodupe , Chem. Phys. Lett. 1997, 265, 481–489.

[28] C. Loschen , G. Frenking , Inorg. Chem. 2004, 43, 778–784. 14731041 10.1021/ic034807e

[29] R. D. Shannon , Acta Crystallogr. A Cryst. Phys. Diffr. Theor. Gen. Crystallogr. 1976, 32, 751–767.

