# Supplementary material for: Quantitative and Chemically Intuitive Evaluation of the Nature of M−L Bonds in Paramagnetic Compounds: Application of EDA‐NOCV Theory to Spin Crossover Complexes
Source: Chemistry. 2020 Sep 24;26(60):13677–85. doi: 10.1002/chem.202002146 (PMC7702084; doi:10.1002/chem.202002146)
Supplement: Supplementary file 1 — Supplementary [file CHEM-26-13677-s001.pdf]

Chemistry–A European Journal

Supporting Information

Quantitative and Chemically Intuitive Evaluation of the Nature of M–L Bonds in Paramagnetic Compounds: Application of EDA-NOCV Theory to Spin Crossover Complexes

Luca Bondì,[a, b] Anna L. Garden,[a] Paul Jerabek,*[c, d] Federico Totti,*[b] and Sally Brooker*[a]

Table of Contents

S1 – Geometry Optimisation Protocol Development S3

S1.1 - Optimised structures of LS and HS states S4

S2 – EDA-NOCV Optimisation Protocol Development S11

S2.1 - Application of EDA-NOCV to paramagnetic complexes S11

S2.2 - Convergence Troubleshooting S11

S2.3 - Symmetry considerations in [Fe^{II}(L^{azine})$_2$($NCBH_3$)$_2$] family S11

S2.4 - Fragmentation 1 - LS [Fe^{II} (L^{azine})$_2$($NCBH_3$)$_2$] S12

S2.5 - Fragmentation 2 - LS [Fe^{II} (L^{azine})$_2$($NCBH_3$)$_2$] S13

S2.6 - Fragmentation 3 - LS [Fe^{II} (L^{azine})$_2$($NCBH_3$)$_2$] S14

S2.7 - Fragmentation 4 - LS [Fe^{II} (L^{azine})$_2$($NCBH_3$)$_2$] S14

S2.8 - L^{azine} vs 2$NCBH_3$: ligand strength comparison in fragmentations 1-4 .... S16

S2.9 - Fragmentation 5a-5e: correct Fe^{2+} energy levels in *LS* [Fe^{II}(L^{azine})$_2$($NCBH_3$)$_2$] family S17

S3 – EDA-NOCV Results S31

S3.1 - Fragmentation 1- *LS* Fe^{II}(L^{azine})$_2$($NCBH_3$)$_2$ S31

S3.2 - Fragmentation 2- *LS* Fe^{II}(L^{azine})$_2$($NCBH_3$)$_2$ S33

S3.3 - Fragmentation 3- *LS* Fe^{II}(L^{azine})$_2$($NCBH_3$)$_2$ S35

S3.4 - Fragmentation 4 - *LS* Fe^{II}(L^{azine})$_2$($NCBH_3$)$_2$ S37

S3.5 - Fragmentation 5b/5e - *LS* Fe^{II}(L^{azine})$_2$($NCBH_3$)$_2$ S39

S3.6 - Fragmentation 5b/5e - *HS* Fe^{II}(L^{azine})$_2$($NCBH_3$)$_2$ S42

S3.7 - Fragmentation 5b/5e - *LS* Fe(L^{azine})$_3^{2+}$ S50

S4 – Fe(L^{azine})$_2$($NCBH_3$)$_2$ COORDINATES S55

***HS* Fe($L^{4pyrimidine}$)$_2$($NCBH_3$)$_2$** S59

***LS* Fe($L^{4pyrimidine}$)$_2$($NCBH_3$)$_2$** S61

***HS* Fe($L^{2pyrimidine}$)$_2$($NCBH_3$)$_2$** S63

***LS* Fe($L^{2pyrimidine}$)$_2$($NCBH_3$)$_2$** S65

***HS* Fe($L^{pyrazine}$)$_2$($NCBH_3$)$_2$** S67

***LS* Fe($L^{pyrazine}$)$_2$($NCBH_3$)$_2$** S69

***HS* Fe($L^{pyridazine}$)$_2$($NCBH_3$)$_2$** S71

***LS* Fe($L^{pyridazine}$)$_2$($NCBH_3$)$_2$** S73

S5 – Fe(L^{azine})$_2$($NCBH_3$)$_2$ COORDINATES S75

***LS* Fe($L^{4pyrimindine}$)$_3$(BF_4)$_2$** S75

***LS* Fe($L^{2pyrimindine}$)$_3$(BF_4)$_2$** S78

***LS* Fe($L^{pyridine}$)$_3$(BF_4)$_2$** S81

***LS* Fe($L^{pyrazine}$)$_3$(BF_4)$_2$** S84

***LS* Fe($L^{pyridazine}$)$_3$(BF_4)$_2$** S87

References S90

S1 – Geometry Optimisation Protocol Development

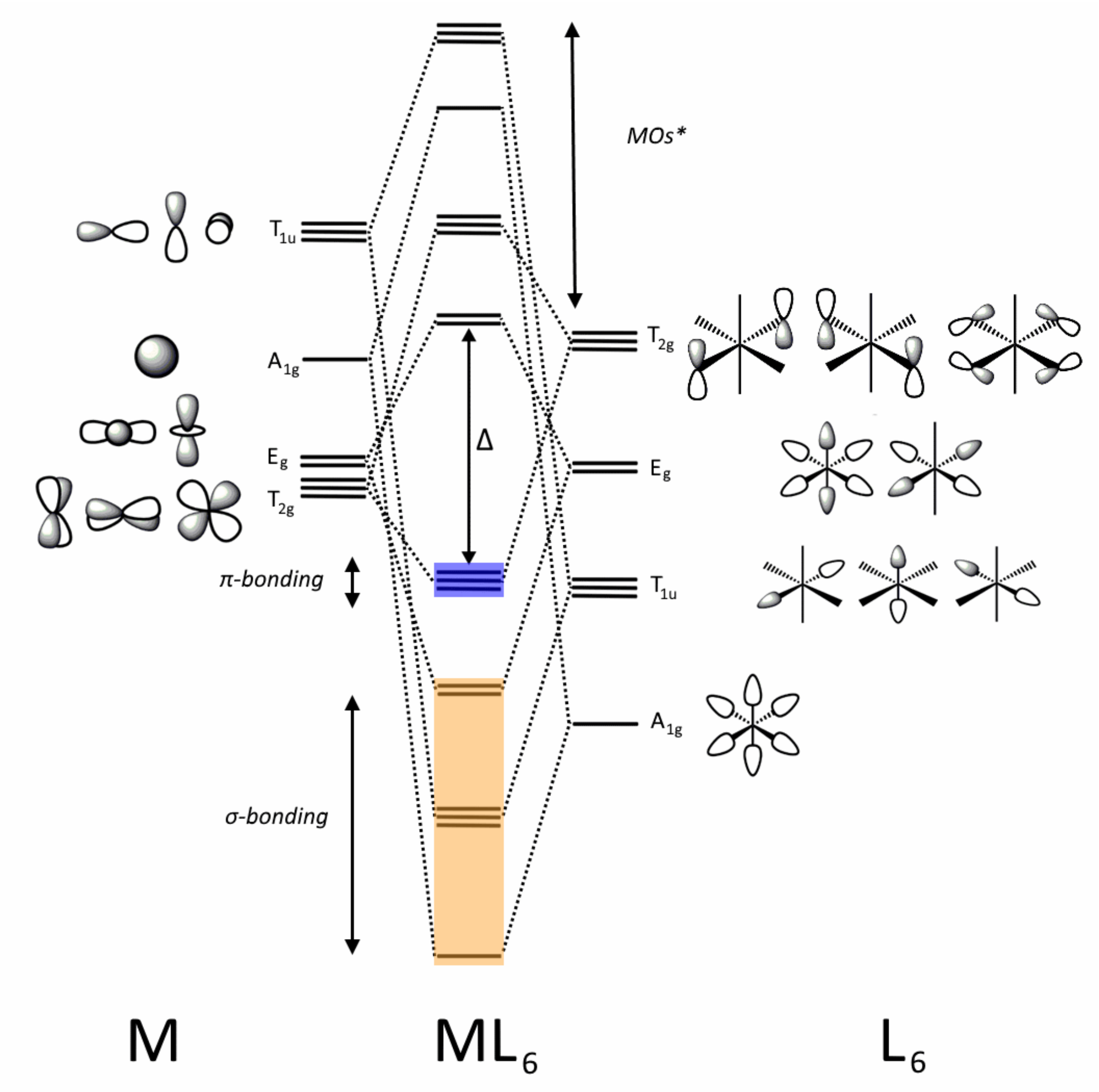

Figure S1. Classic qualitative representation of the ladder of σ- and π-MO energies (centre) for a perfect octahedral (O_h symmetry) **ML₆** complex which results from overlap of the valence AOs of **M** (left) with the **L₆** MOs of the same symmetry (right). For the sake of readability: on the left the **M** AO's are shown as already split into E_g and T_{2g} (but should be degenerate), and on the right the six **L₆** symmetry adapted linear combinations (SALCs) forming the σ-bonds with **M** are not shown as energetically degenerate (but should be).

S1.1 - Optimised structures of LS and HS states

As a first step, accurate structures for these complexes in both the low-spin (*LS*) and high-spin (*HS*) states are required, so density functional theory (DFT) calculations were performed, with the ORCA 4.1 software package,[1] to determine the optimal computational setup required to obtain these (Table S1, Figure 2).[2] Four different functionals (B3LYP, BP86, reVPBE, RPBE)[3] were tested, as well as the possible inclusion of dispersion effects, *via* a D3 dispersion correction including Becke-Johnson damping (BJ),[4] and of solvent effects ($CHCl_3$), by using the conductor-like polarizable continuum model (CPCM).[5] To determine the best combination of these, the optimized structures obtained were compared to the single-crystal X-ray structure data available for [Fe^{II}($\boldsymbol{L}^{pyridine}$)$_2$($NCBH_3$)$_2$] in both the *LS* and *HS* states,[2] with particular attention given to three key parameters: Fe-N distance, Fe-N≡C(BH_3) angle and the root-mean-square-deviation (RMSD) of all coordinates (Table S2). Deviations from the experimentally observed linear Fe-N≡C(BH_3) were observed for most of the tested combinations, especially when the complex was in the paramagnetic *HS* state (see Table S2 and Figures S2-S9). The best combination was determined to be BP86-D3(BJ)/def2-TZVPP+CPCM($CHCl_3$).[6, 7] i.e. use of a BP86 functional, with D3 dispersion correction (including BJ damping), def2-TZVPP basis set,[1, 8, 9] and the solvent modelled by CPCM. This protocol was therefore the one used to provide all of the optimized structures used as the start point for the subsequent EDA-NOCV analyses performed using ADF package[10] (Tables S1-S3).

Table S1. Summary of the combinations of computational features used to determine the best computational set up for the geometry optimization using ORCA code, with the best combination highlighted.

Name	***Functional***				***Basis Set***	***D3BJ***	***Solvent ($CHCl_3$)***
	B3LYP	***BP86***	***reVPBE***	***RPBE***	***TZVPP***		
T.B3	X				X		
T.B3.D	X				X	X	
T.B3.s	X				X		X
T.B3.D.s	X				X	X	X
T.BP		X			X		
T.BP.D		X			X	X	
T.BP.s		X			X		X
T.BP.D.s		X			X	X	X
T.RV			X		X		
T.RV.D			X		X	X	
T.RV.s			X		X		X
T.RV.D.s			X		X	X	X
T.RP				X	X		
T.RP.D				X	X	X	
T.RP.s				X	X		X
T.RP.D.s				X	X	X	X

Table S2. RMSD values ($Å^2$) and Fe-$NCBH_3$ Axial Angle – referred to crystal structure of both candidates – of the final optimized structures of [Fe($\boldsymbol{L}^{pyridine}$)$_2$($NCBH_3$)$_2$] in both the *HS* and *LS* states, with the method of choice highlighted in blue.

Name	***HS***		***LS***	
	RMSD	***Fe-NC(BH_3) Angle***	***RMSD***	***Fe-($NCBH_3$) Angle***
REF	-	175.44	-	177.61
T.B3	0.1867	156.6	0.2187	166.98
T.B3.D	0.1876	156.8	0.2976	153.37
T.B3.s	0.1807	155.6	0.2357	171.45
T.B3.D.s	0.1827	155.7	0.2491	169.44
T.BP	0.3970	156.72	0.2959	174.39
T.BP.D	0.3218	149.39	0.4893	179.07
T.BP.s	0.1978	177.50	0.2457	153.41
T.BP.D.s	0.2142	178.97	0.2602	177.99
T.RV	0.3977	158.54	0.3615	169.13
T.RV.D	0.3254	141.00	0.4893	154.35
T.RV.s	0.2019	177.71	0.2374	179.24
T.RV.D.s	0.3371	152.84	0.2477	177.15
T.RP	0.1815	158.16	0.2959	168.68
T.RP.D	0.1787	148.79	0.4893	157.19
T.RP.s	0.1027	177.70	0.2457	179.38
T.RP.D.s	0.2602	153.20	0.1152	178.28

Table S3. Fe-N bond distances (Å) and Fe-N≡C(BH_3) angles in the *LS* (*HS*) states of the experimentally determined structures of [Fe(***L***^{pyridine})$_2$($NCBH_3$)$_2$] and in the calculated structures of all five [Fe(***L***^{azine})$_2$($NCBH_3$)$_2$] complexes obtained by DFT at the BP86-D3(BJ)/def2-TZVPP (+CPCM) level of theory, along with the observed $T_{1/2}$ and calculated N_A chemical shift.

L^{azine}		**Fe-N$_A$**	**Fe-N$_{tr}$**	**Fe-NCBH$_3$**	**Fe-N≡C(BH$_3$)**	**T$_{1/2}$**	**δ(N$_A$)**
L^{pyridine}	**Exp.**	2.02 (2.18)	1.98 (2.12)	1.95 (2.14)	177.6 (175.3)	**288**	**300**
L^{pyridine}	**Calc.**	1.99 (2.15)	1.95 (2.13)	1.90 (2.10)	178.2 (179.2)		
L^{4pyrimidine}	**Calc.**	1.99 (2.16)	1.94 (2.13)	1.90 (2.09)	179.3 (179.2)	**232**	**269**
L^{2pyrimidine}	**Calc.**	1.98 (2.15)	1.94 (2.13)	1.90 (2.09)	179.0 (179.0)	**262**	**282**
L^{pyrazine}	**Calc.**	**1.97** (2.15)	1.95 (2.13)	1.90 (2.09)	178.3(179.3)	**327**	**327**
L^{pyridazine}	**Calc.**	**1.95** (2.15)	1.95 (2.15)	1.90 (2.09)	177.6(179.3)	**455**	**402**

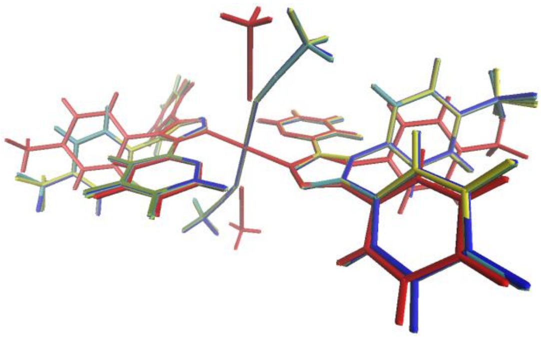

Legend:

- **RED** – crystalline structure
- **YELLOW** – B3.D.s
- **BLUE** – B3
- **CYANO** – B3.D
- **GREEN** – B3.s

Figure S2. Superimposed structures for *HS* [Fe(***L**^pyridine^*)$_2$(NCBH$_3$)$_2$]. The experimentally determined single crystal X-ray structure (**red**) is compared with those calculated by DFT using the def2-TZVPP basis set and a B3LYP density functional alone (**blue**), or in combination with other terms: worst combination B3LYP+D3(BJ) (**cyan**); B3LYP +CPCM (**yellow**); optimal combination B3LYP + D3(BJ)+CPCM (**green**).

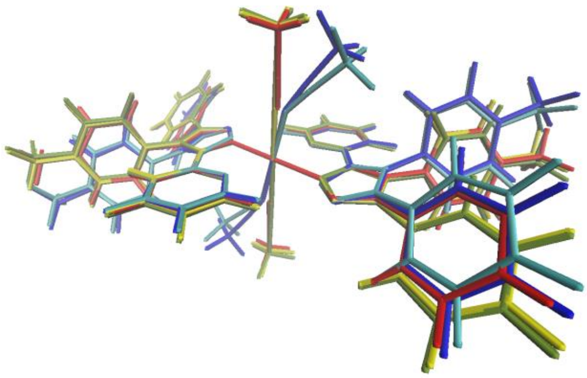

Legend:

- **RED** – crystalline
- **YELLOW** – BP.D.s
- **BLUE** – BP
- **CYANO** – BP.D
- **GREEN** – BP.s

Figure S3. Superimposed structures for *HS* [Fe(***L**^pyridine^*)$_2$(NCBH$_3$)$_2$]. The experimentally determined single crystal X-ray structure (**red**) is compared with those calculated by DFT using the def2-TZVPP basis set and a BP86 density functional alone (**blue**), or in combination with other terms: worst combination BP86+D3(BJ) (**cyan**); BP86+CPCM (**yellow**); optimal combination BP86+ D3(BJ)+CPCM (**green**).

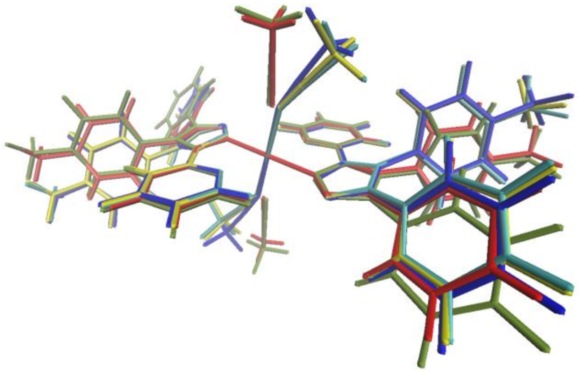

Legend:

- **RED** – crystalline structure
- **YELLOW** – RV.D.s
- **BLUE** – RV
- **CYANO** – RV.D
- **GREEN** – RV.s

Figure S4. Superimposed structures for *HS* [Fe($\boldsymbol{L}^{pyridine}$)$_2$(NCBH$_3$)$_2$]. The experimentally determined single crystal X-ray structure (**red**) is compared with those calculated by DFT using the def2-TZVPP basis set and a reVPBE density functional alone (**blue**), or in combination with other terms: worst combination reVPBE+D3(BJ) (**cyan**); reVPBE +CPCM (**yellow**); optimal combination BP86+ D3(BJ)+CPCM (**green**).

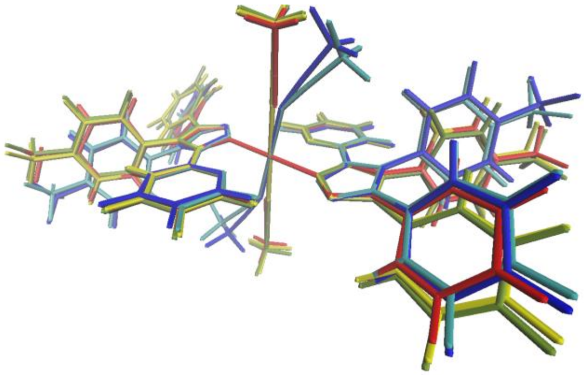

Legend:

- **RED** – crystalline structure
- **YELLOW** – B3.D.s
- **BLUE** – B3
- **CYANO** – B3.D
- **GREEN** – B3.s

Figure S5. Superimposed structures for *HS* [Fe($\boldsymbol{L}^{pyridine}$)$_2$(NCBH$_3$)$_2$]. The experimentally determined single crystal X-ray structure (**red**) is compared with those calculated by DFT using the def2-TZVPP basis set and a RPBE density functional alone (**blue**), or in combination with other terms: worst combination RPBE+D3(BJ) (**cyan**); RPBE+CPCM (**yellow**); optimal combination RPBE + D3(BJ)+CPCM (**green**).

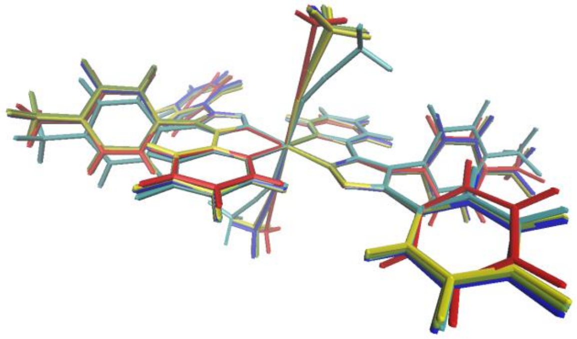

Legend:

- **RED** – crystalline structure
- **YELLOW** – B3.D.s
- **BLUE** – B3
- **CYANO** – B3.D
- **GREEN** – B3.s

Figure S6. Superimposed structures for *LS* [Fe($\boldsymbol{L}^{pyridine}$)$_2$(NCBH$_3$)$_2$]. The experimentally determined single crystal X-ray structure (**red**) is compared with those calculated by DFT using the def2-TZVPP basis set and a B3LYP density functional alone (**blue**), or in combination with other terms: worst combination B3LYP+D3(BJ) (**cyan**); B3LYP +CPCM (**yellow**); optimal combination B3LYP + D3(BJ)+CPCM (**green**).

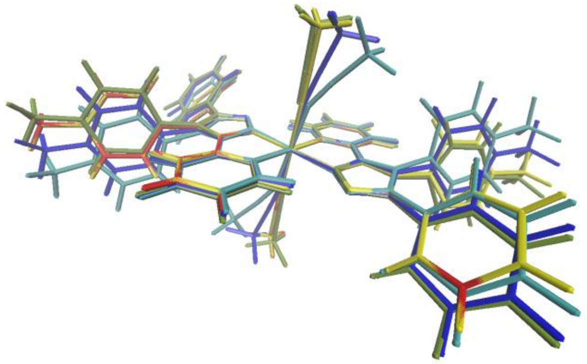

Legend:

- **RED** – crystalline structure
- **YELLOW** – BP.D.s
- **BLUE** – BP
- **CYANO** – BP.D
- **GREEN** – BP.s

Figure S7 Superimposed structures for *LS* [Fe($\boldsymbol{L}^{pyridine}$)$_2$(NCBH$_3$)$_2$]. The experimentally determined single crystal X-ray structure (**red**) is compared with those calculated by DFT using the def2-TZVPP basis set and a BP86 density functional alone (**blue**), or in combination with other terms: worst combination BP86+D3(BJ) (**cyan**); BP86+CPCM (**yellow**); optimal combination BP86+ D3(BJ)+CPCM (**green**).

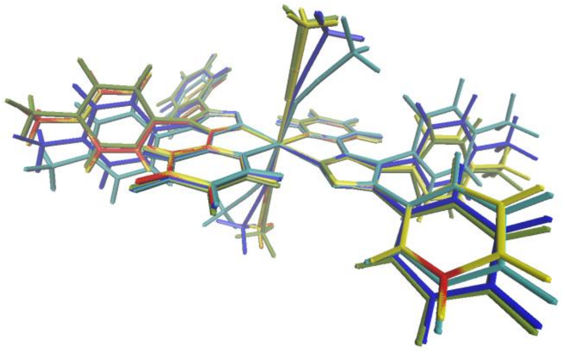

Legend:
- **RED** – crystalline structure
- **YELLOW** – B3.D.s
- **BLUE** – B3
- **CYANO** – B3.D
- **GREEN** – B3.s

Figure S8. Superimposed structures for *LS* [Fe($\boldsymbol{L}^{pyridine}$)$_2$(NCBH$_3$)$_2$]. The experimentally determined single crystal X-ray structure (**red**) is compared with those calculated by DFT using the def2-TZVPP basis set and a reVPBE density functional alone (**blue**), or in combination with other terms: worst combination reVPBE+D3(BJ) (**cyan**); reVPBE +CPCM (**yellow**); optimal combination BP86+ D3(BJ)+CPCM (**green**).

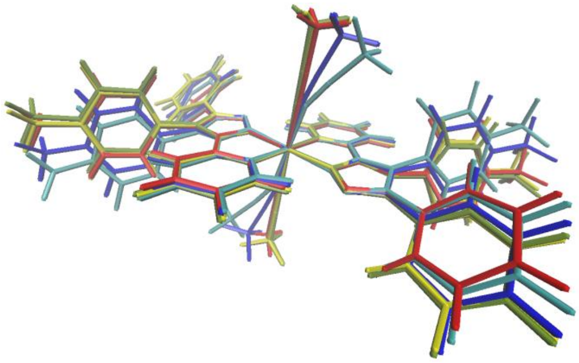

Legend:
- **RED** – crystalline structure
- **YELLOW** – B3.D.s
- **BLUE** – B3
- **CYANO** – B3.D
- **GREEN** – B3.s

Figure S9. Superimposed structures for *LS* [Fe($\boldsymbol{L}^{pyridine}$)$_2$(NCBH$_3$)$_2$]. The experimentally determined single crystal X-ray structure (**red**) is compared with those calculated by DFT using the def2-TZVPP basis set and a RPBE density functional alone (**blue**), or in combination with other terms: worst combination RPBE+D3(BJ) (**cyan**); RPBE+CPCM (**yellow**); optimal combination RPBE + D3(BJ)+CPCM (**green**).

S2 – EDA-NOCV Optimisation Protocol Development

S2.1 - Application of EDA-NOCV to paramagnetic complexes

EDA-NOCV analysis is rigorously applied to an open shell (paramagnetic) metal system – specifically to the family of $[Fe^{II}(\boldsymbol{L^{azine}})_2(NCBH_3)_2]$ complexes in the *high spin* state. Here this is done in order to observe the changes which occur as a result of a spin state change; here from diamagnetic *LS* Fe^{II} (S=0) to paramagnetic *HS* Fe^{II} (S=4). But the general approach developed herein should be applicable to other paramagnetic complexes. In contrast to the *LS* Fe^{II} systems (above), the treatment of the open-shell *HS* Fe^{II} systems requires the use of separate *alpha* and *beta* electrons during all of the calculations. Hence, the NOCV deformation densities $\boldsymbol{\Delta\rho_i}$ are also split into *alpha*- and *beta*- contributions (Figure S28); they are then merged to obtain the final values (as reported in Table S22).

S2.2 - Convergence troubleshooting

Convergence issues were met in attempts to prepare the $[Fe(\boldsymbol{L^{azine}})_2]^{2+}$ and $[Fe(NCBH_3)_2]$ fragments (for fragmentations **3** and **4**) with the correct occupancy of the *d* orbitals. To overcome this problem, fractional charges (0.2 e^-, distance from Fe 2.00 Å) were introduced and placed at the coordinates, relative to Fe(II), of the coordinating **N** of the $NCBH_3^-$ and $\boldsymbol{L^{azine}}$ ligands.

S2.3 - Symmetry considerations in $[Fe^{II}(\boldsymbol{L^{azine}})_2(NCBH_3)_2]$ family

Starting from the ideal and correct description of the M-L bond in Hoffman's theory[11] where fragments are treated in their native electronic state and chargeless, EDA-NOCV imposes two method constraints which have forced the authors to introduce approximations in order to perform a bond analysis using this model. Firstly, according to the Hoffmann **M-L** bond description,[11, 12] the metal fragment should be treated in its own native state (Fe^0). This condition cannot be applied in EDA-NOCV analysis as the model imposes agreement between the oxidation state between each fragment and the final system; consequently Fe^{2+} species must be used instead of Fe^0. A possible 'trick' to overcome this constraint is reported below in fragmentation **5e**. Secondly, according to the Hoffmann **M-L** bond description,[11, 12] in the most faithful description of the **M**(*AOs*) prior to bonding the five metal *d* orbitals would be degenerate each other (spherical symmetry). Unfortunately, in order to provide the correct *d*-orbital occupancies, in EDA-NOCV analysis a symmetry reduction must be imposed.

For all the *LS* species into this study where a ($d_{xy}^2\ d_{xz}^2\ d_{yz}^2\ d_{x2-y2}^0 d_{z2}^0$) electronic structure is requested, a symmetry reduction from *spherical* to O_h is enough (Table S4). This symmetry

applied to the un-coordinated metal ion introduces a small bias, due to the introduction of a t_{2g}-e_g gap at this earlier stage. But in the case of the *HS* systems the use of O_h symmetry is not enough, as it does not grant a unique allocation of the only *beta* electron in the former t_{2g} orbitals. Due to the requirement to unequivocally allocate electrons into fragment orbitals, the Fe^{2+} fragment was prepared by further reducing the symmetry, from O_h to C_{2v}, and choosing to assign the double occupation to the d_{xy} orbital ($d_{xy}^2\ d_{xz}^1\ d_{yz}^1\ d_{x2-y2}^1\ d_{z2}^1$) (Table S6). This choice is justified by the fact that the d_{xy} orbital is found to be the lowest orbital in the *d* set in the final complex.

S2.4 - Fragmentation 1 - LS [Fe^{II} (L^{azine})$_2$(NCBH$_3$)$_2$]

This fragmentation (removal of one **NCBH$_3^-$**) enables the details of the bonding between the **ML$_5$** fragment and a single **NCBH$_3^-$** co-ligand to be probed. **Δ*E*$_{elstat}$** and **Δ*E*$_{orb}$** are quantities that give indications of the *ionic* and *covalent* character of the chemical bond(s) formed between the two fragments, respectively. As expected, due to the charged nature of the $NCBH_3^-$ co-ligand, the interaction is mainly ionic (**Δ*E*$_{elstat}$:Δ*E*$_{orb}$** = ca. 70:30) (Figure S11 and Table S17). Furthermore, the σ- and π-contributions to **Δ*E*$_{orb}$** are 75:25 **Δ*E*$_{orb,σ}$:Δ*E*$_{orb,π}$**. The EDA-NOCV analysis reveals three interactions (deformation densities $\Delta\rho_{(i)}$), forming one σ-type-bond and two π-type-bonds between the *LS* Fe^{II} and $NCBH_3^-$ anion (Figure S10 and Figure S23, Table S17). The σ-interaction (Figure S10, left) occurs between the unoccupied **ML$_5$** (*MOs*) with high Fe(d_{z2}) character and the occupied $NCBH_3^-$(*MOs*) with high **N$_{NCBH3}$** lone pair character. The two π-acceptor interactions (Figure S10, right, 1 of these) occur between the occupied **ML$_5$**(*MOs*) with high Fe(d_{xz} *and* d_{yz}) character and the unoccupied $NCBH_3^-$ (*MOs*).

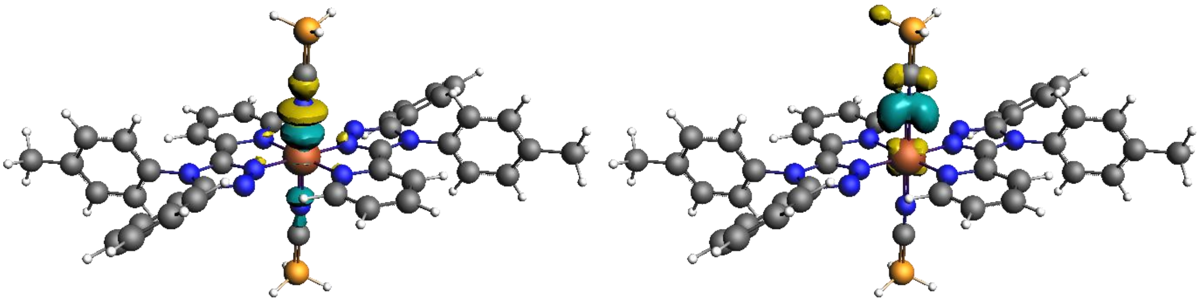

Figure S10. Plot of the deformation densities $\Delta\rho_{(i)}$ (reported using cut-off on $\Delta\rho_{(i)}$ of 0.003) in fragmentation **1** of *LS* Fe(***L**^{pyridine}*)$_2$(NCBH$_3$)$_2$ corresponding to the [TM]←ligand σ-donation (top), the [TM]→ligand π-backdonation (bottom). The direction of the charge flow is yellow → turquoise.

The total of σ- and π-type orbital interactions **Δ*E*$_{orb,σ+π}$** found for the $NCBH_3^-$ co-ligand is practically constant at 50-51 kcal/mol across the entire family (independent of the choice of ***L**^{azine}*). Hence the role of the $NCBH_3^-$ apical ligands in the modulation of the SCO process appears constant, as expected given that it is ***L**^{azine}* that is being varied (Figure S23, Table S17).

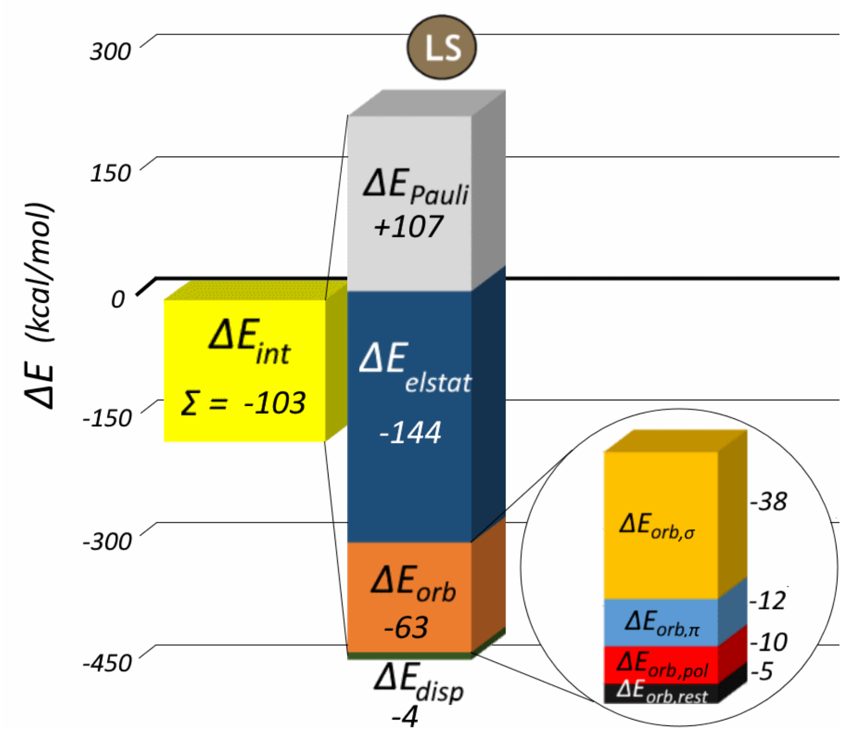

Figure S11. Results of EDA-NOCV for *LS* [Fe($\boldsymbol{L}^{pyridine}$)$_2$($NCBH_3$)$_2$] using fragmentation **1**: (left) yellow column is total ΔE_{int} and middle column is components of ΔE_{int} (eq. 1); (right) expansion showing contributions to ΔE_{orb} (eq. 2). Energies are in kcal/mol.

S2.5 - Fragmentation 2 - LS [Fe^{II} (L^{azine})$_2$($NCBH_3$)$_2$]

Fragmentation **2** considers the removal of a single neutral bidentate $\boldsymbol{L}^{azine}$ ligand (Figure S24, Table S18). Clearly, this fragmentation was expected to be the most valuable with regard to establishing the relative ligand field strength of each of these five $\boldsymbol{L}^{azine}$ ligands. Indeed, EDA-NOCV analysis appears to detect the differences in the electronic structure in the $\boldsymbol{L}^{azine}$ ligands (Figure S24, Table S18), but the differences in energy are very small (4 kcal/mol), so should be taken with caution. The $\boldsymbol{\Delta E}_{\mathbf{orb},\sigma+\pi}$ values show a trend consistent with the expected ligand field increases across the series (Table S18), with the $\boldsymbol{L}^{4pyrimidine}$complex (lowest $T_{1/2}$) experiencing the weakest (-85.2 kcal/mol) and the $\boldsymbol{L}^{pyridazine}$ complex (highest $T_{1/2}$) the strongest ligand field (-89.0 kcal/mol) (Figure S24, Table S18), respectively. Both the σ- and π-contributions, $\boldsymbol{\Delta E}_{\mathbf{orb},\sigma}$ and $\boldsymbol{\Delta E}_{\mathbf{orb},\pi}$, also follow this trend (Figure S24, Table S18), with both steadily increasing on going from $\boldsymbol{L}^{4pyrimidine}$across to $\boldsymbol{L}^{pyridazine}$, again consistent with the experimentally observed steadily increasing $T_{1/2}$ (and hence ligand field). The $\boldsymbol{L}^{pyridine}$ ligand is out of line with this trend, by about 2 kcal/mol, but this is not surprising, as it is not simply an isomer of the other azines: While four of the others are *diazines*, pyridine contains only one N atom in the six-membered ring. The ratio of $\boldsymbol{\Delta E}_{\mathbf{orb},\sigma}$ and $\boldsymbol{\Delta E}_{\mathbf{orb},\pi}$ is about 75:25, regardless of the $\boldsymbol{L}^{azine}$ involved in the **M-**$\boldsymbol{L}^{azine}$ bonds being formed (Figure S24, Table S18).

S2.6 - Fragmentation 3 - LS [Fe^{II} (L^{azine})$_2$($NCBH_3$)$_2$]

To avoid spurious contributions to the EDA coming from the presence of additional ligands of the same kind still being present in the **ML_x** fragment, fragmentations **3** and **4** were trialled. In the case of fragmentation **3** (removal of both **$NCBH_3^-$**, Figure S25, Table S19), the energies of the Fe(*d*) orbitals only experience the different strength of the ***L^{azine}*** ligands *within* the **ML_2^{azine}** fragment, not *between* the two fragments. The minimal differences observed for fragmentation **1** are enhanced enough in fragmentation **3** to give a clearer trend of field strength for the family of ***L^{azine}*** complexes. Specifically, **$\Delta E_{orb,\sigma+\pi}$** is observed to steadily decrease from ***$L^{4pyrimidine}$*** (-95.7 kcal/mol) through to ***$L^{pyridazine}$*** (-98.1 kcal/mol) as expected from the trend in $T_{1/2}$ (Figure 1, Figure S25, Table S19), with just ***$L^{pyridine}$*** (-95.0 kcal/mol) representing a discontinuity in the trend, as it is not a *diazine*.

S2.7 - Fragmentation 4 - LS [Fe^{II} (L^{azine})$_2$($NCBH_3$)$_2$]

Moving forward to fragmentation 4 we expect better results, as it better reflects the chemical point of view[2] - as experimentally $Fe(NCBH_3)_2$ reacts with two equivalents of ***L^{azine}*** - and it should enhance the differences between the members of the ***L^{azine}*** family, whilst maintaining a constant electronic structure for the other fragment, **ML_2** = $Fe(NCBH_3)_2$. Analysis of the σ- and π-contributions (Figure S12, Figure S26, Table S20) shows that the σ-interaction is almost three times larger than the π-interaction regardless of ***L^{azine}***.

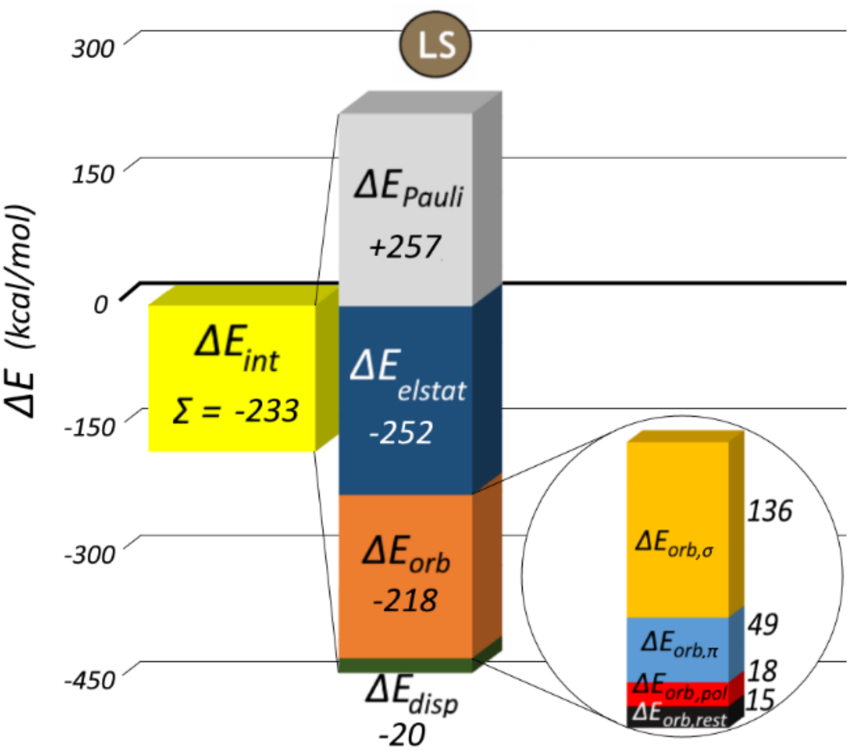

Figure S12. Results of EDA-NOCV for *LS* [Fe(***$L^{pyridine}$***)$_2$($NCBH_3$)$_2$] using fragmentation **4**: (left) yellow column is total ΔE_{int} and middle column is components of ΔE_{int} (eq. 1); (right) expansion showing contributions to ΔE_{orb} (eq. 2). Energies are in kcal/mol.

The σ-strength ($\Delta E_{orb,\sigma}$) of the L^{azine} ligands follows the order (Table S20):

$L^{pyridazine} > L^{pyrazine} > L^{pyridine} > L^{4pyrimidine} > L^{2pyrimidine}$.

This matches the observed order of $T_{1/2}$ until the pyrimidines are considered; they are in the reverse order (Figure 1).[13] Interestingly the order of the π-strength ($\Delta E_{orb,\pi}$) of the L^{azine} ligands differs (and the values are far from showing a monotonic trend):

$L^{pyrazine} > L^{pyridazine} > L^{4pyrimidine} > L^{2pyrimidine} > L^{pyridine}$.

This order bears no relationship to the observed order of $T_{1/2}$ values (Figure 1, Table S20). Crucial is the collective contribution of $\Delta E_{orb,\sigma}$ and $\Delta E_{orb,\pi}$, $\Delta E_{orb,\sigma+\pi}$, as this describes the total effect of the pair of L^{azine} ligands on the metal ion in the final complex correctly (Figure S12, Figure S26, Table S20):

$L^{pyridazine} > L^{pyrazine} > L^{pyridine} > L^{2pyrimidine} > L^{4pyrimidine}$.

Indeed, an extremely strong correlation between the $\Delta E_{orb,\sigma+\pi}$ term and the experimental $T_{1/2}$ values ($R^2 = 0.99$) is observed (Figure S13 red).

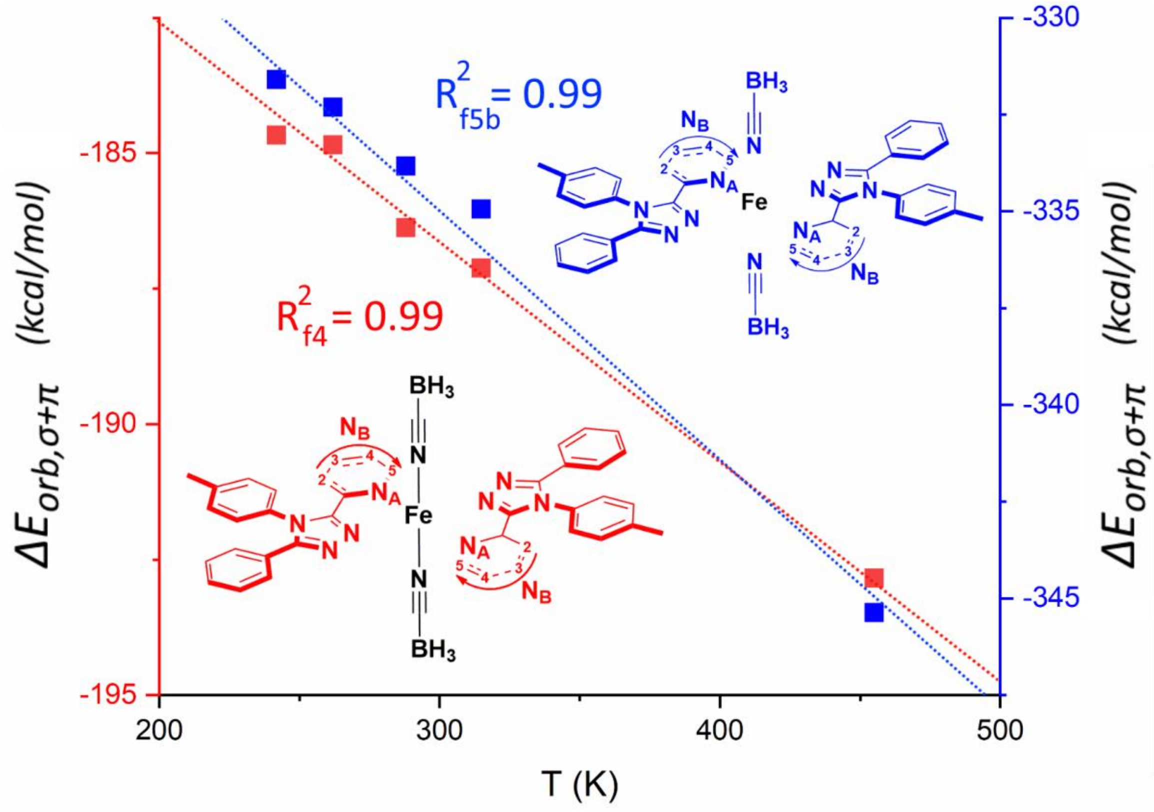

Figure S13. Linear correlation between the experimental $T_{1/2}$ values of the *LS* [Fe($L^{pyridine}$)$_2$(NCBH$_3$)$_2$] complexes and the $\Delta E_{orb,\sigma+\pi}$ values (sum of σ- and π- character orbital interactions between the fragments), using fragmentations **4** (red, $R^2 = 0.99$) and **5b** (blue, $R^2 = 0.99$).

S2.8 - L^{azine} vs 2NCBH₃ : ligand strength comparison in fragmentations 1-4

Regardless of $\boldsymbol{L^{azine}}$, comparisons of **$\Delta E_{orb,\sigma+\pi}$** for fragmentations **1** (**$NCBH_3^-$: -**48 to -49 kcal/mol) vs **2** ($\boldsymbol{L^{azine}}$: -85 to -89 kcal/mol) vs **3** (2x**$NCBH_3^-$** -95 to -98 kcal/mol) vs **4** (2x$\boldsymbol{L^{azine}}$ -183 to -193 kcal/mol), consistently show that the **$\Delta E_{orb,\sigma+\pi}$** for two **$NCBH_3^-$** ligands (-95 to -98 kcal/mol) contributes a similar or slightly larger stabilization energy than one bidentate $\boldsymbol{L^{azine}}$ (-85 to -97 kcal/mol) does (Figures S14). This is inconsistent with the above experimental observations, which clearly show that bidentate $\boldsymbol{L^{azine}}$ actually possesses a stronger ligand field ligand than 2x**$NCBH_3^-$** (monodentate).

This disagreement between the theoretical results and experimental data highlights why fragmentations **1-4** are not good choices for such a comparison. It occurs because of these fragmentation choices not sharing a constant reference fragment (and hence lacking generality). If the strength of a ligand is to be assessed then this can be done only if the rest of the coordination sphere is maintained unaltered. This is achieved by employing fragmentation **5**, in the form of **5b**, to obtain **$\Delta E_{orb,\sigma+\pi}$**.

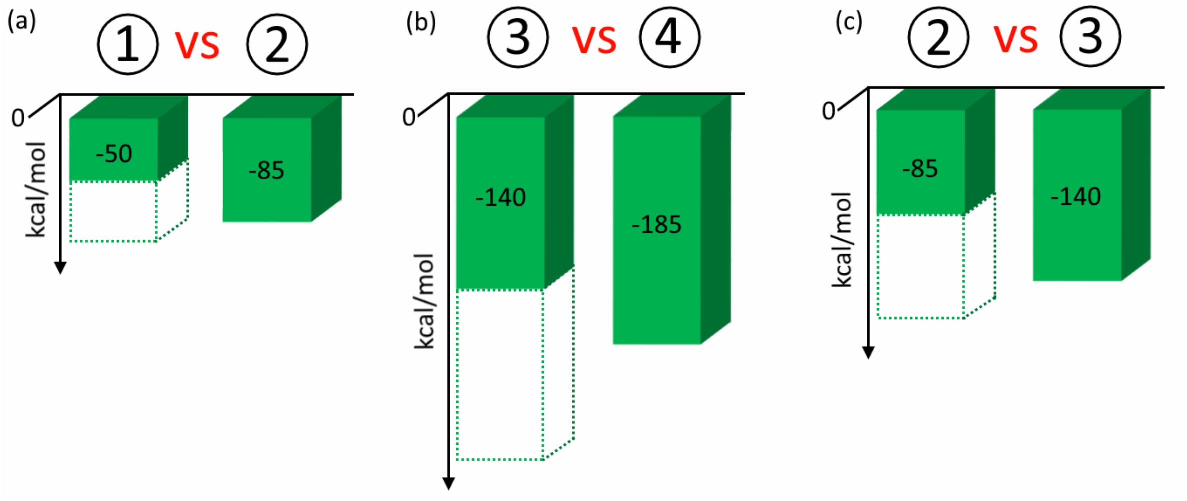

Figure S14. $\boldsymbol{\Delta E_{orb,\sigma+\pi}}$ results (kcal/mol) across fragmentation 1-4; scheme leads comparing theoretical results versus experimental evidences on the ligand strength of **$NCBH_3^-$** versus $\boldsymbol{L^{azine}}$ for *LS* [Fe ($\boldsymbol{L^{pyridine}}$)$_2$($NCBH_3$)$_2$] complex. Results are consistent for all the other four *LS* [Fe($\boldsymbol{L^{azine}}$)$_2$($NCBH_3$)$_2$]. (a) $\boldsymbol{\Delta E_{orb,\sigma+\pi}}$ comparison between fragmentation 1 and 2. (b) $\boldsymbol{\Delta E_{orb,\sigma+\pi}}$ comparison between fragmentation 3 and 4. (c) $\boldsymbol{\Delta E_{orb,\sigma+\pi}}$ comparison between fragmentation 2 and 3. Dotted boxes help to visualise twice the amount of $\boldsymbol{\Delta E_{orb,\sigma+\pi}}$ for specific fragmentation.

S2.9 - Fragmentation 5a-5e: correct Fe^{2+} energy levels in *LS* [$Fe^{II}(L^{azine})_2(NCBH_3)_2$] family

Whilst the energies of the Fe *d* orbitals in fragmentations **1**-**4** have comparable energies to the valence orbitals of the ligands, as expected within Hoffman's MO diagram (see Figure S1), thanks to the partial ligand fields induced by the ligands included in the fragments, this does not happen in fragmentation **5**. In this case, the Fe^{2+} *d* atomic energies, for fragmentation **5a** (no modifications/corrections) are calculated to be unrealistically low in energy, approx. -26.0 *eV* (Table S4), with respect to the MO energies of the ligands, -4.0 to +4.0 *eV* (Table S5). This huge difference in energy is unacceptable in Hoffman's MO diagram, and may well present a bias in the EDA analysis. So, to overcome this problem the free ion $\mathbf{M^{n+}}$ in **5a** was instead treated by four different methods **5b** to **5e** in order to determine which generated the most appropriate Fe^{2+} *d* atomic energy levels:

- Fragmentation **5a.** Fe^{2+} ($\mathbf{t_{2g}}$: -26.0 *eV*; $\mathbf{e_g}$: -25.6 *eV*).
- Fragmentation **5b.** Fe^{2+} + 6x -0.425e ($\mathbf{t_{2g}}$: -8.0 *eV*; $\mathbf{e_g}$: -7.61 *eV*).
- Fragmentation **5c.** Fe^{2+} + 6x -1.0e ($\mathbf{t_{2g}}$: +16.4 *eV*; $\mathbf{e_g}$: +16.9 *eV*).
- Fragmentation **5d.** Fe^{2+} +6x -2.0e ($\mathbf{t_{2g}}$: +58.7 *eV*; $\mathbf{e_g}$: +59.6 *eV*).
- Fragmentation **5e.** Fe^{2+} density mapped onto Fe^0(*AOs*) ($\mathbf{t_{2g}}$: -8.0 *eV*; $\mathbf{e_g}$: -7.8 *eV*).

In three of these fragmentations, **5b-5d**, six negative charges are placed octahedrally around the Fe^{2+} ion at a distance of 2.00 Å. The magnitude of the charges was tuned (from -0.425e^- to -1e^- to -2e^- each) in order to obtain Fe^{2+}(*AO*) energies closer to the ligand *MO* energies (Table S5); the best fragmentation is **5b** (approx. -8.0 *eV*). This occurs at the cost of introducing a small t_{2g}-e_g splitting but this has the advantage of ensuring the correct occupancy of the *d*-orbitals in the final complex with marginal effects on the final absolute energy of the Fe^{2+} fragment (Table S4). But, fragmentation of **5b** suffers from underestimating the $\mathbf{\Delta}\boldsymbol{E}_{\mathbf{Pauli}}$ (see later) so an additional fragmentation, **5e**, was also developed. Here the wavefunction obtained by calculation of the Fe^0 in an octahedral symmetry (Table S4) was manually manipulated to simply remove two electrons from 4s orbital, *i.e.* Fe^0($3d^64s^2$) to Fe^{2+}($3d^64s^0$), whilst retaining the calculated energy levels (which are appropriate in Hoffman's MO diagram), in preparation for EDA-NOCV analysis.

To our knowledge, such a fragmentation at the level we present it here represents a novelty in the EDA-NOCV analysis of transition metal complexes. Indeed, to apply this approach successfully, first the problem of accurate representation of the *d* orbitals energies had to be addressed and hence several computational setups were trialed for fragmentation **5** (see also above). In fragmentations **5a-5e** comparison each term of the EDA-NOCV analysis was proved a full understanding of the relationship between Fe orbitals and EDA-NOCV energy terms. Fragmentations **5a-5d** (octhaedral negative charges from 0e to 2e) do not show substantial changes in any of the EDA-NOCV (Tables S7-S16 and Figures S15-S21) terms: $\mathbf{\Delta}\boldsymbol{E}_{\mathbf{int}}$ and $\mathbf{\Delta}\boldsymbol{E}_{\mathbf{Pauli}}$ are shifted by charges inclusion in the Fe^{2+} fragment, but regardless of this the trend across the family is maintained (Tables S12-S16 and

Figures S15-S16). In contrast, for **Δ*E*orb** and **Δ*E*elstat** a trend inversion is reported: increasing the charge intensity around the metal ion caused **Δ*E*orb** to decrease slightly, whilst **Δ*E*elstat** rose (Tables S12-S16 and Figures S17-S18). This trend inversion only introduces differences of around 1-2% so is not significant. Similarly, **$\Delta E_{orb,\sigma}$** decreased and **$\Delta E_{orb,\pi}$** increased as the the charge intensity around the Fe^{2+}(*AOs*) was increased by charge inclusion (Tables S12-S16 and Figures S19-S20). Once the Fe^{2+}(*AOs*) energies were raised above reasonable levels (fragmentations **5c-5d**) **$\Delta E_{orb,\pi}$** becomes extremely small (almost negligible) or even positive (Tables S12-S16 and Figures S20) consistent with a shift of the formally "t_{2g}" *d* orbitals far above the *diazine* π* orbital energies, inhibiting π-back donation and leading to σ-only interactions. Interestingly, the **$\Delta E_{orb,\sigma+\pi}$** term maintains a stable value across fragmentations **5a-5d** (Tables S12-S16 and Fig. S21) despite the variations in single constitutive **$\Delta E_{orb,\pi}$** and **$\Delta E_{orb,\sigma}$** contributions which change due to the different setups (partial charges) used to handle the Fe^{2+} ion.

Hence fragmentation **5e**, in which the density of the Fe^{2+}(*AOs*) was mapped onto the Fe^0 (*AOs*), to emulate a molecular orbital interaction in Hoffmann's framework,[11] was also trialed. The EDA-NOCV analysis results from this fragmentation are very different from those observed for all of the previous fragmentations **5a-5d**. A remarkable difference was observed in the **ΔE_{Pauli}** contribution, which rises from about 250 kcal/mol (fragmentations **5a-5d**) to more than 600 kcal/mol (Tables S16 and Figure S15). Consequently, **ΔE_{int}** decreases considerably, indicating the quality of the new densities (Tables S16 and Figure S15), since smaller **ΔE_{int}** indicates smaller dissociation energies for the involved fragments, and, therefore, fragment densities that are closer to the final one. The same range of **ΔE_{int}** values as found in **5a-5e** have been reported in a previous study in literature when **M^0** or **M^{2+}** are used. Furthermore, in the same study the authors point out that the use of a **M^{2+}** fragment in EDA can introduce a bias into the results. The choice of the correct representation of **ΔE_{Pauli}** energy should be the one where the calculated amount is comparable (often higher) than **ΔE_{elstat}**. **ΔE_{elstat}** + **ΔE_{Pauli}** can be considered as the lost energy (consequently with positive (+) contribution) to be overtaken by **Δ*E*orb** to engage a new bond. From this angle, fragmentation **5e** gives the most correct representation in the EDA analysis as it avoids to underestimate **ΔE_{Pauli}** as for fragmentation **5b**.

On other hand, when results obtained from NOCV are analyzed considerable error is observed. Deformation density **$\Delta\rho_3$** (identified as a d_{xz} π-backdonation) unphysically positive and considerably stronger than the two other π-interactions (occurring through d_{yz} and d_{xy}) (Table S11, Table S16). This result would drop to zero the entire π-strength of the coordination sphere with unique contribution of the metal ion through σ-bonds (except for Fe(***L****pyridazine*)$_2$(NCBH$_3$)$_2$). A similar error in this π-interaction (as that specific interaction would be de-stabilizing term) was observed, indeed, in fragmentation **5d** when Fe^{2+} are risen to +50 *eV* (Table S15). However, **$\Delta E_{orb,\sigma+\pi}$** values are in line with the ones computed in any **5** fragmentation (Figure S21). The correlation established earlier in fragmentation **4** between **$\Delta E_{orb,\sigma+\pi}$** and the experimental $T_{1/2}$ is reported also for fragmentations **5** where, despite the fluctuations observed into σ- and π-interactions when Fe^{2+} is treated differently, the correlation shows $R^2 > 0.95$ for all the **5** fragmentations (Figure S21).

In summary, considering all of the data reported above, fragmentations **5b** and **5e** appear to be the most accurate for two different types of analysis: fragmentation **5b** shows the best results for the NOCV analysis – but the error in the underestimation of the ΔE_{Pauli} has to be reported. On the other hand, fragmentation **5e** corrects this error and it provides the best EDA analysis in order to get information on the energies of bond(s) formed between the fragments. They should both be employed, as together this provides the best description of EDA **5e** and NOCV **5b** analysis.

Table S4. Results reported for the analysis of the *LS Fe(AOs)* energies (*eV*) frontier orbitals used for establish the most correct EDA-NOCV analysis in the **M**+L_6 fragmentation. The best are fragmentations are **5b** and **5e** as the Fe^{2+} orbital energies are close to those of the **L** (-4.0 to 4.0 *eV*; Table S5). †Energy levels for **5e** come directly from those calculated for Fe^0 (O_H); the only difference is that the 2 *s* electrons have been manually removed in preparation for EDA-NOCV analysis.

LS Fe (O_H)	*T_{2g}*	*E_g*	*$\Delta E(E_g$-$T_{2g})$*	*Energy (H)*	*Fragmentation*
Fe^0 (Spherical Symmetry)	**-7.93**	**-7.93**	**0.0**	-1263.66	-
Fe^0 (O_H)	-7.96†	-7.78†	0.18†	-1263.66	-
Fe^{2+} (no charges)	-26.05	-25.61	0.56	-1262.74	***5a***
Fe^{2+} (6x -0.425e)	-8.00	-7.61	0.39	-1262.75	***5b***
Fe^{2+} (6x -1.0e)	+16.37	+16.94	0.57	-1262.75	***5c***
Fe^{2+} (6x -2.0e)	+58.68	+59.55	0.87	-1262.74	***5d***
Fe^{2+} on Fe^0(AOs)	-7.96†	-7.78†	0.18†	-1263.66	***5e***

Table S5. Reported energies (*eV*) of the MO of the L_6 coordination sphere who interact with *Fe(AOs)*. Each MO(L_6) is paired with the relative *Fe(AOs)* in bracket. MOs pairing was obtained by analysis of the contributing SFOs from density flowing ($\Delta\rho_{(i)}$) in EDA-NOCV analysis.

	MO(d_{x2-y2})	*MO(d_{z2})*	*MO(d_{xz})*	*MO(d_{zy})*	*MO(d_{xy})*	*MO(p_x)*	*MO(p_y)*	*MO(p_z)*	*MO(s)*
$L^{4pyrimidine}$	0.20	0.53	2.62	3.26	**-**	-0.73	-0.37	-0.35	-2.98
$L^{2pyrimidine}$	0.27	0.54	2.78	2.93	-	-0.66	-0.35	-0.35	-1.55
$L^{pyridine}$	0.41	0.74	3.47	3.10	-	-0.56	-0.23	-0.23	-1.71
**$L^{pyrazine}$**	0.19	0.48	2.58	3.07	-	-0.75	-0.43	-0.42	-3.19
**$L^{pyridazine}$**	0.99	0.53	2.94	2.91	-	-0.39	0.14	-0.23	-1.13

Table S6. Results reported for the analysis of the *HS Fe(AOs)* energies (*eV*) frontier orbitals used for establish the most correct EDA-NOCV analysis in the **M**+L_6 fragmentation.

HS Fe (C_{2v})	*d_{xy}*	*d_{xz}*	*d_{zy}*	*d_{x2-y2}*	*d_{z2}*	*Fragmentation*
Fe^0 (Spherical Symmetry)	**-7.93**	**-7.93**	**-7.93**	**-7.93**	**-7.93**	-
Fe^{2+} (no charges)	-26.05	-26.31	-26.31	-25.46	-26.58	***5a***
Fe^{2+} (6x -0.425e)	-7.28	-8.13	-8.13	-7.13	-8.25	***5b***
Fe^{2+} on Fe^0(AOs)	-7.42	-8.23	-8.23	-7.44	-8.47	***5e***

Table S7. EDA-NOCV results (kcal/mol) across the five fragmentations **5a-5e** for the treatment of the isolated metal ion **M**. Results for *LS* [Fe($L^{4pyrimidine}$)$_2$($NCBH_3$)$_2$] are reported. Decomposition in specific contribution of ΔE_{int} (top), and ΔE_{orb} (bottom) are reported.

	5a	*5b*	*5c*	*5d*	*5e*
ΔE_{int}	-865.6	-865.8	-866.7	-870.3	-505.1
ΔE_{Pauli}	264.4	263.9	263.1	261.4	631.0
ΔE_{elstat}	-609.1	-610.6	-612.8	-617.1	-622.4
ΔE_{orb}	-511.3	-509.6	-507.4	-505.0	-504.1
$\Delta E_{orb,\sigma}$	-301.9	-304.4	-308.0	-314.9	-321.3
$\Delta E_{orb,\pi}$	-33.1	-28.7	-22.8	-12.9	-0.2
$\Delta E_{orb,\sigma+\pi}$	-334.9	-333.0	-330.8	-327.7	-321.5

Table S8. EDA-NOCV results (kcal/mol) across the five fragmentations **5a-5e** for the treatment of the isolated metal ion **M**. Results for *LS* [Fe($L^{2pyrimidine}$)$_2$($NCBH_3$)$_2$] are reported. Decomposition in specific contribution of ΔE_{int} (top), and ΔE_{orb} (bottom) are reported.

	5a	*5b*	*5c*	*5d*	*5e*
ΔE_{int}	-865.9	-866.1	-867.0	-870.3	-505.5
ΔE_{Pauli}	265.9	265.5	264.6	-262.9	632.3
ΔE_{elstat}	-611.4	-612.9	-615.1	-619.4	-624.7
ΔE_{orb}	-510.9	-509.1	-507.0	-504.6	-503.6
$\Delta E_{orb,\sigma}$	-303.5	-305.9	-312.1	-315.7	-322.1
$\Delta E_{orb,\pi}$	-31.7	-27.4	-21.4	-15.3	0.6
$\Delta E_{orb,\sigma+\pi}$	-335.2	-333.3	-333.5	-331.0	-321.4

Table S9. EDA-NOCV results (kcal/mol) across the five fragmentations **5a-5e** for the treatment of the isolated metal ion **M**. Results for *LS* [Fe($L^{pyridine}$)$_2$($NCBH_3$)$_2$] are reported. Decomposition in specific contribution of ΔE_{int} (top), and ΔE_{orb} (bottom) are reported.

	5a	*5b*	*5c*	*5d*	*5e*
ΔE_{int}	-876.7	-876.9	-877.8	-881.4	-516.0
ΔE_{Pauli}	262.7	262.9	262.1	-260.3	630.0
ΔE_{elstat}	-618.7	-619.5	-621.7	-625.9	-631.1
ΔE_{orb}	-511.2	-510.6	-508.5	-506.2	-505.2
$\Delta E_{orb,\sigma}$	-302.2	-308.1	-311.6	-318.2	-325.0
$\Delta E_{orb,\pi}$	-33.8	-27.3	-21.6	-12.2	0.1
$\Delta E_{orb,\sigma+\pi}$	-335.9	-335.4	-333.2	-330.4	-324.9

Table S10. EDA-NOCV results (kcal/mol) across the five fragmentations **5a-5e** for the treatment of the isolated metal ion **M**. Results for *LS* [Fe($\boldsymbol{L}^{pyrazine}$)$_2$($NCBH_3$)$_2$] are reported. Decomposition in specific contribution of ΔE_{int} (top), and ΔE_{orb} (bottom) are reported.

	5a	*5b*	*5c*	*5d*	*5e*
ΔE_{int}	-864.3	-864.5	-865.4	-869.0	-503.9
ΔE_{Pauli}	268.0	267.6	266.8	-265.0	634.5
ΔE_{elstat}	-607.5	-609.1	-611.3	-615.6	-620.9
ΔE_{orb}	-515.2	-513.4	-511.2	-508.8	-507.8
$\Delta E_{\text{orb},\sigma}$	-304.6	-307.3	-311.1	-317.9	-324.2
$\Delta E_{\text{orb},\pi}$	-33.7	-29.2	-23.0	-13.0	-0.7
$\Delta E_{\text{orb},\sigma+\pi}$	-338.3	-336.5	-341.3	-330.9	-324.9

Table S11. EDA-NOCV results (kcal/mol) across the five fragmentations **5a-5e** for the treatment of the isolated metal ion **M**. Results for *LS* [Fe($\boldsymbol{L}^{pyridazine}$)$_2$($NCBH_3$)$_2$] are reported. Decomposition in specific contribution of ΔE_{int} (top), and ΔE_{orb} (bottom) are reported.

	5a	*5b*	*5c*	*5d*	*5e*
ΔE_{int}	-885.4	-883.0	-883.9	-887.5	-522.4
ΔE_{Pauli}	268.4	-271.11	-270.3	-268.6	-638.0
ΔE_{elstat}	-607.7	-623.0	-625.3	-629.6	-634.9
ΔE_{orb}	-515.4	-521.6	-519.5	-517.1	-516.1
$\Delta E_{\text{orb},\sigma}$	-290.9	-289.1	-292.8	-301.4	-308.5
$\Delta E_{\text{orb},\pi}$	-55.4	-55.4	-48.5	-37.1	-23.4
$\Delta E_{\text{orb},\sigma+\pi}$	-346.2	-344.5	-341.3	-338.4	-331.9

Table S12. EDA-NOCV results (kcal/mol) reported for fragmentation **5a** for all the five *LS* [Fe($\boldsymbol{L}^{azine}$)$_2$(NCBH$_3$)$_2$] systems. First section (top) reports $\boldsymbol{\Delta E_{int}}$ energy splitting; second section (middle) reports $\boldsymbol{\Delta E_{orb}}$ energy splitting; third section (bottom) reports all the nine orbitalic interaction due to **M** + **L$_6$** interaction.

	$L^{4pyrimidine}$	$L^{2pyrimidine}$	$L^{pyridine}$	$L^{pyrazine}$	$L^{pyridazine}$
ΔE_{int}	-865.6	-865.9	-876.7	-864.3	-885.4
ΔE_{Pauli}	265.9	265.9	262.7	268.0	268.4
ΔE_{elstat}	-609.1 (53.9%)	-611.4 (54.0%)	-618.7 (54.3%)	-607.5 (53.6%)	-607.7 (53.7%)
ΔE_{orb}	-511.3 (45.2%)	-510.9 (45.1%)	-511.2 (44.8%)	-515.2 (45.5%)	-515.4 (45.5%)
ΔE_{disp}	-9.6 (0.9%)	-9.5 (0.9%)	-9.7 (0.9%)	-9.6 (0.9%)	-9.4 (0.8%)
$\Delta E_{orb,\sigma}$	-301.9 (59.1%)	-303.5 (59.3%)	-302.2 (59.1%)	-304.6 (59.2%)	-290.9 (56.5%)
$\Delta E_{orb,\pi}$	-33.1 (6.5%)	-31.7 (6.3%)	-33.8 (6.7%)	-33.7 (6.6%)	-55.4 (10.8%)
$\Delta E_{orb,\sigma+\pi}$	-334.9 (65.6%)	-335.2 (65.6%)	-335.9 (65.8%)	-338.3 (65.8%)	-346.2 (67.3%)
$\Delta E_{orb,pol}$	-146.6 (28.8%)	-148.9 (29.2%)	-145.9 (28.6%)	-147.2 (28.5%)	-158.7 (30.9%)
$\Delta E_{orb,rest}$	-29.8 (5.6%)	-29.8 (5.2%)	-29.4 (5.8%)	-29.4 (5.7%)	-31.2 (5.8%)
$\Delta E_{orb,dz2}$	-112.7	-112.3	-113.0	-111.3	-111.5
$\Delta E_{orb,dx2-y2}$	-115.2	-116.6	-114.6	-118.1	-103.0
$\Delta E_{orb,dzx}$	-5.3	-4.5	-6.0	-5.2	-26.9
$\Delta E_{orb,dzy}$	-12.1	-11.4	-12.4	-12.8	-12.0
$\Delta E_{orb,dxy}$	-15.7	-15.8	-15.4	-15.7	-16.4
$\Delta E_{orb,s}$	-23.3	-23.1	-23.6	-23.6	-23.2
$\Delta E_{orb,pz}$	-18.8	-19.3	-19.3	-19.7	-21.2
$\Delta E_{orb,px}$	-18.5	-19.0	-18.2	-19.1	-18.9
$\Delta E_{orb,py}$	-13.3	-13.2	-13.4	-12.8	-13.1

Table S13. EDA-NOCV results (kcal/mol) reported for fragmentation **5b** for all the five *LS* [Fe($\boldsymbol{L}^{azine}$)$_2$($NCBH_3$)$_2$] systems. First section (top) reports $\boldsymbol{\Delta E_{int}}$ energy splitting; second section (middle) reports $\boldsymbol{\Delta E_{orb}}$ energy splitting; third section (bottom) reports all the nine orbitalic interaction due to **M** + $\mathbf{L_6}$ interaction.

	$L^{4pyrimidine}$	$L^{2pyrimidine}$	$L^{pyridine}$	$L^{pyrazine}$	$L^{pyridazine}$
ΔE_{int}	-866.1	-876.9	-864.5	-883.0	-866.1
ΔE_{Pauli}	265.5	262.9	267.6	271.11	265.5
ΔE_{elstat}	-610.6 (54.0%)	-612.8 (54.1%)	-619.5 (54.4%)	-609.1 (53.7%)	-623.0 (53.8%)
ΔE_{orb}	-509.6 (45.1%)	-509.1 (45.0%)	-510.6 (44.7%)	-513.4 (45.4%)	-521.6 (45.4%)
ΔE_{disp}	-9.6 (0.9%)	-9.5 (0.9%)	-9.7 (0.9%)	-9.6 (0.9%)	-9.4 (0.8%)
$\Delta E_{orb,\sigma}$	-304.3 (59.6%)	-305.9 (60.1%)	-308.1 (60.3%)	-307.3 (60.4%)	-289.1 (56.4%)
$\Delta E_{orb,\pi}$	-28.7 (5.7%)	-27.4 (5.3%)	-27.3 (5.3%)	-29.2 (4.7%)	-55.4 (8.8%)
$\Delta E_{orb,\sigma+\pi}$	-333.0 (65.3%)	-333.3 (65.4%)	-335.4 (65.6%)	-336.5 (65.1%)	-344.5 (65.2%)
$\Delta E_{orb,pol}$	-146.5 (28.6%)	-145.8 (28.7%)	-145.2 (28.4%)	-146.9 (24.1%)	-146.6 (23.6%)
$\Delta E_{orb,rest}$	-30.6 (5.9%)	-33.5 (5.9%)	-31.6 (6.0%)	-30.5 (5.8%)	-31.3 (4.9%)
$\Delta E_{orb,dz2}$	-113.8	-113.4	-112.8	-112.5	-102.2
$\Delta E_{orb,dx2-y2}$	-116.3	-117.7	-120.4	-119.3	-110.3
$\Delta E_{orb,dzx}$	-3.4	-2.6	-1.3	-3.2	-29.2
$\Delta E_{orb,dzy}$	-10.8	-10.1	-11.2	-11.5	-10.9
$\Delta E_{orb,dxy}$	-14.6	-14.7	-14.9	-14.6	-15.3
$\Delta E_{orb,s}$	-23.4	-23.2	-23.8	-23.6	-23.3
$\Delta E_{orb,pz}$	-18.8	-19.4	-19.3	-19.8	-21.3
$\Delta E_{orb,px}$	-18.6	-19.0	-19.3	-19.1	-19.0
$\Delta E_{orb,py}$	-13.4	-13.3	-13.5	-12.9	-13.1

Table S14. EDA-NOCV results (kcal/mol) reported for fragmentation **5c** for all the five *LS* [Fe(L^{azine})$_2$(NCBH$_3$)$_2$] systems. First section (top) reports ΔE_{int} energy splitting; second section (middle) reports ΔE_{orb} energy splitting; third section (bottom) reports all the nine orbitalic interaction due to **M** + **L$_6$** interaction.

	$L^{4pyrimidine}$	$L^{2pyrimidine}$	$L^{pyridine}$	$L^{pyrazine}$	$L^{pyridazine}$
ΔE_{int}	-867.0	-877.8	-865.4	-883.9	-867.0
ΔE_{Pauli}	263.1	264.6	262.0	266.7	270.3
ΔE_{elstat}	-612.8 (54.2%)	-615.1 (54.3%)	-621.6 (54.6%)	-611.3 (53.9%)	-625.3 (54.0%)
ΔE_{orb}	-507.4 (44.9%)	-507.0 (44.8%)	-508.5 (44.5%)	-511.2 (45.2%)	-519.5 (45.2%)
ΔE_{disp}	-9.6 (0.9%)	-9.5 (0.9%)	-9.7 (0.9%)	-9.6 (0.9%)	-9.4 (0.8%)
$\Delta E_{orb,\sigma}$	-308.0 (60.7%)	-312.1 (61.5%)	-311.6 (61.4%)	-311.1 (60.9%)	-292.8 (56.3%)
$\Delta E_{orb,\pi}$	-22.7 (3.8%)	-21.5 (3.4%)	-21.6 (4.3%)	-23.0 (4.5%)	-48.5 (9.3%)
$\Delta E_{orb,\sigma+\pi}$	-330.8 (64.5%)	-333.5 (64.9%)	-333.2 (65.7%)	-334.2 (65.4%)	-341.3 (65.6%)
$\Delta E_{orb,pol}$	-146.6 (29.0%)	-143.4 (28.2%)	-145.4 (28.5%)	-147.1 (28.8%)	-148.2 (28.5%)
$\Delta E_{orb,rest}$	-31.4 (6.5%)	-33.6 (6.9%)	-29.3 (6.8%)	-30.9 (6.8%)	-31.3 (6.9%)
$\Delta E_{orb,dz2}$	-115.6	-115.0	-114.3	-114.3	-105.1
$\Delta E_{orb,dx2-y2}$	-117.9	-119.3	-122.0	-121.1	-111.6
$\Delta E_{orb,dzx}$	-0.7	-0.1	+1.2	-0.3	-25.6
$\Delta E_{orb,dzy}$	-9.0	-8.2	-9.4	-9.7	-9.1
$\Delta E_{orb,dxy}$	-13.1	-13.2	-13.4	-13.1	-13.7
$\Delta E_{orb,s}$	-23.4	-23.2	-23.9	-23.7	-23.3
$\Delta E_{orb,pz}$	-18.9	-19.5	-19.4	-19.9	-20.4
$\Delta E_{orb,px}$	-18.7	-19.2	-18.4	-19.2	-19.1
$\Delta E_{orb,py}$	-13.5	-16.0	-13.6	-13.0	-13.2

Table S15. EDA-NOCV results (kcal/mol) reported for fragmentation **5d** for all the five *LS* [Fe($\boldsymbol{L}^{azine}$)$_2$($NCBH_3$)$_2$] systems. First section (top) reports $\boldsymbol{\Delta E_{int}}$ energy splitting; second section (middle) reports $\boldsymbol{\Delta E_{orb}}$ energy splitting; third section (bottom) reports all the nine orbitalic interaction due to **M** + **L$_6$** interaction.

	$L^{4pyrimidine}$	$L^{2pyrimidine}$	$L^{pyridine}$	$L^{pyrazine}$	$L^{pyridazine}$
**Δ*E*$_{int}$**	-870.6	-881.4	-869.0	-887.5	-870.6
**Δ*E*$_{Pauli}$**	261.4	262.9	260.3	265.0	268.6
**Δ*E*$_{elstat}$**	-617.1 (54.5%)	-619.4 (54.6%)	-625.9 (54.9%)	-615.6 (54.2%)	-629.6 (54.3%)
**Δ*E*$_{orb}$**	-505.0 (44.6%)	-504.6 (44.5%)	-506.2 (44.2%)	-508.8 (44.9%)	-517.1 (45.5%)
**Δ*E*$_{disp}$**	-9.6 (0.9%)	-9.5 (0.9%)	-9.7 (0.9%)	-9.6 (0.9%)	-9.4 (0.8%)
**Δ*E*$_{orb,\sigma}$**	-314.9 (62.4%)	-315.7 (62.6%)	-318.2 (62.8%)	-317.9 (62.5%)	-301.4 (58.2%)
**Δ*E*$_{orb,\pi}$**	-12.9 (2.6%)	-15.3 (3.0%)	-12.2 (2.4%)	-13.0 (2.6%)	-37.1 (7.1%)
**Δ*E*$_{orb,\sigma+\pi}$**	-327.7 (65.0%)	-331.0 (65.6%)	-330.4 (65.2%)	-330.9 (65.1%)	-338.4 (65.3%)
**Δ*E*$_{orb,pol}$**	-147.3 (29.1%)	-143.6 (28.5%)	-145.8 (28.9%)	-147.9 (29.1%)	-148.6 (28.8%)
**Δ*E*$_{orb,rest}$**	-31.0 (5.9%)	-33.8 (5.9%)	-30.8 (5.9%)	-30.9 (5.8%)	-31.6 (5.9%)
**Δ*E*$_{orb,dz2}$**	-118.9	-118.0	-117.1	-117.6	-110.4
**Δ*E*$_{orb,dx2-y2}$**	-121.0	-122.5	-125.1	-124.4	-114.3
**Δ*E*$_{orb,dzx}$**	+3.9	+4.3	+5.5	+4.5	-19.4
**Δ*E*$_{orb,dzy}$**	-6.1	-5.3	-6.5	-6.8	-6.2
**Δ*E*$_{orb,dxy}$**	-10.6	-14.3	-11.2	-10.7	-11.4
**Δ*E*$_{orb,s}$**	-23.4	-23.1	-24.0	-23.7	-23.3
**Δ*E*$_{orb,pz}$**	-19.0	-19.1	-19.6	-19.6	-20.5
**Δ*E*$_{orb,px}$**	-18.9	-19.4	-18.6	-19.5	-19.4
**Δ*E*$_{orb,py}$**	-13.7	-13.6	-13.8	-13.2	-13.4

Table S16. EDA-NOCV results (kcal/mol) reported for fragmentation **5e** for all the five *LS* [Fe($\boldsymbol{L}^{azine}$)$_2$($NCBH_3$)$_2$] systems. First section (top) reports $\boldsymbol{\Delta E_{int}}$ energy splitting; second section (middle) reports $\boldsymbol{\Delta E_{orb}}$ energy splitting; third section (bottom) reports all the nine orbitalic interaction due to **M** + $\mathbf{L_6}$ interaction.

	$L^{4pyrimidine}$	$L^{2pyrimidine}$	$L^{pyridine}$	$L^{pyrazine}$	$L^{pyridazine}$
ΔE_{int}	-505.1	-505.5	-516.0	-503.9	-522.4
ΔE_{Pauli}	631.0	632.3	630.0	634.5	638.0
ΔE_{elstat}	-622.4 (54.9%)	-624.7 (55.0%)	-631.1 (55.3%)	-620.9 (54.6%)	-634.9 (54.7%)
ΔE_{orb}	-504.1 (44.5%)	-503.6 (44.4%)	-505.2 (44.1%)	-507.8 (44.8%)	-516.1 (44.4%)
ΔE_{disp}	-9.6 (0.6%)	-9.5 (0.6%)	-9.7 (0.6%)	-9.6 (0.6%)	-9.4 (0.9%)
$\Delta E_{orb,\sigma}$	-321.3 (63.7%)	-322.1 (63.8%)	-325.0 (64.4%)	-324.2 (63.8%)	-308.5 (59.7%)
$\Delta E_{orb,\pi}$	-0.2 (0.0%)	+0.6 (-0.1%)	+0.1 (0.0%)	-0.7 (-0.1%)	-23.4 (4.5%)
$\Delta E_{orb,\sigma+\pi}$	-321.5 (63.7%)	-321.4 (63.7%)	-324.9 (64.4%)	-324.9 (63.7%)	-331.9 (64.1%)
$\Delta E_{orb,pol}$	-152.5 (30.4%)	-152.2 (30.2%)	-150.3 (29.7%)	-156.3 (30.7%)	-164.6 (32.0%)
$\Delta E_{orb,rest}$	-31.4 (5.9%)	-35.5 (5.9%)	-31.1 (5.9%)	-31.2 (5.6%)	-32.0 (5.9%)
$\Delta E_{orb,dz2}$	-124.2	-123.2	-122.1	-122.9	-117.1
$\Delta E_{orb,dx2-y2}$	-126.3	-128.0	-130.5	-130.0	-119.2
$\Delta E_{orb,dzx}$	+10.2	+10.5	+11.7	+11.1	-12.2
$\Delta E_{orb,dzy}$	-2.3	-1.3	-2.7	-3.0	-2.4
$\Delta E_{orb,dxy}$	-8.1	-8.5	-8.9	-8.9	-8.7
$\Delta E_{orb,s}$	-23.0	-22.7	-24.1	-23.5	-23.3
$\Delta E_{orb,pz}$	-19.2	-19.8	-19.7	-19.6	-19.6
$\Delta E_{orb,px}$	-14.8	-14.7	-14.6	-14.9	-16.0
$\Delta E_{orb,py}$	-13.8	-13.7	-13.9	-13.3	-13.4

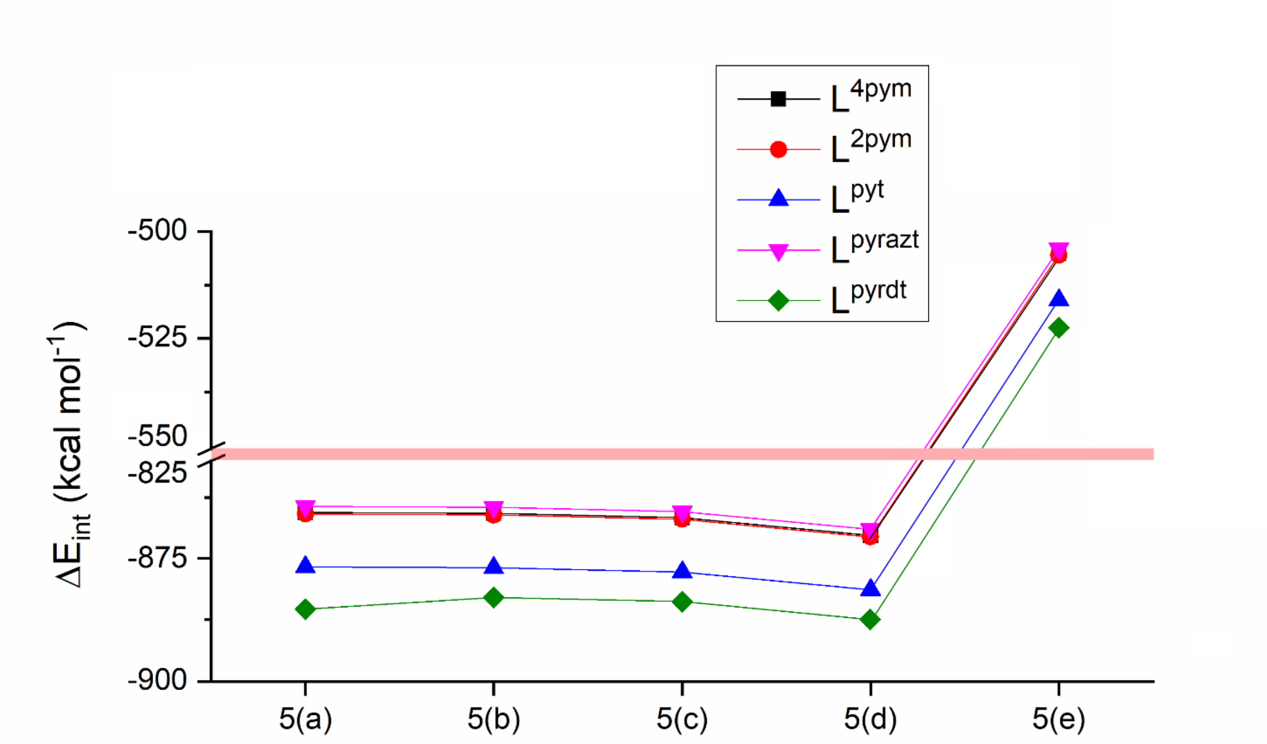

Figure S15. Trend of $\boldsymbol{\Delta E_{\text{int}}}$ energy contribution (kcal/mol) for each of the five *LS* [Fe($\boldsymbol{L^{azine}}$)$_2$(NCBH$_3$)$_2$] systems across the five sub fragmentations **5a-5e**.

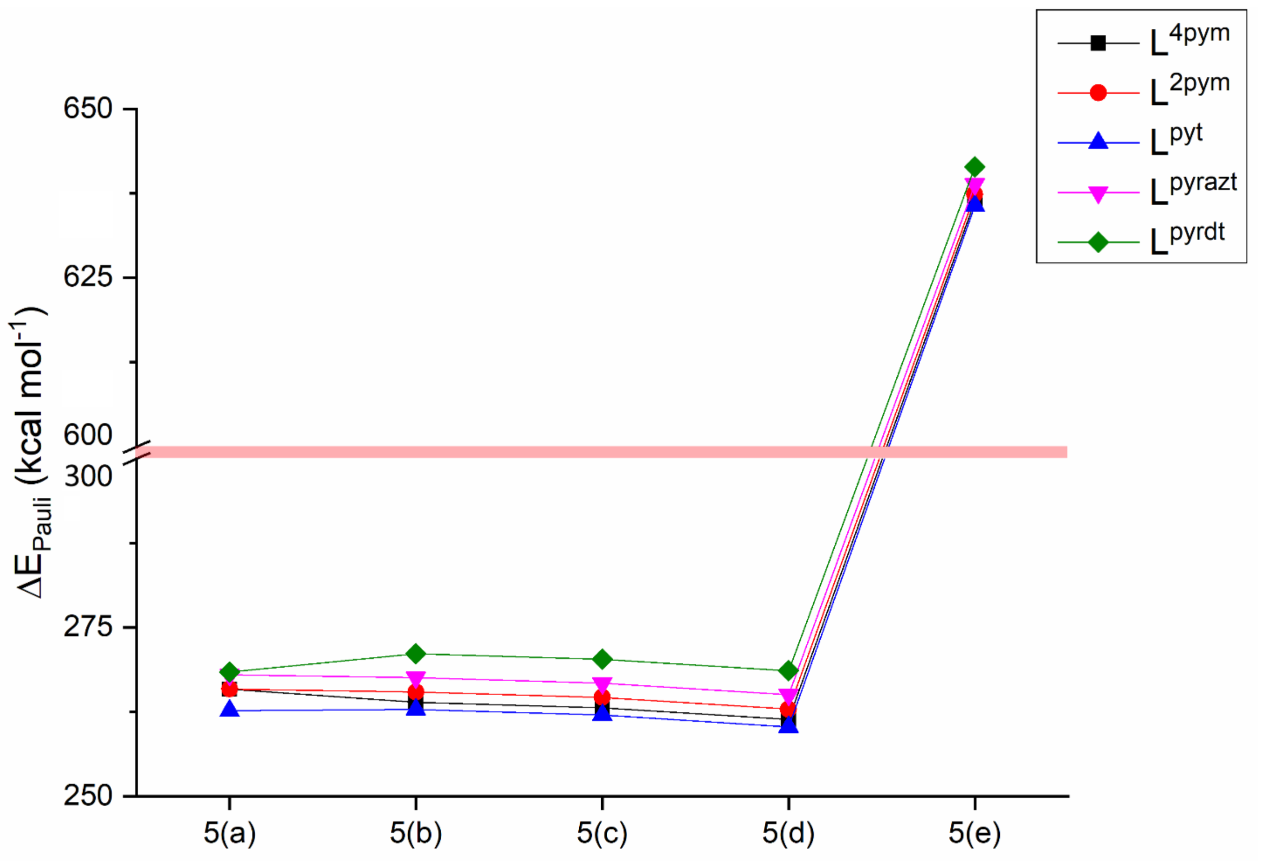

Figure S16. Trend of $\boldsymbol{\Delta E_{\text{Pauli}}}$ energy contribution (kcal/mol) for each of the five *LS* [Fe($\boldsymbol{L^{azine}}$)$_2$(NCBH$_3$)$_2$] systems across the five sub fragmentations **5a-5e**.

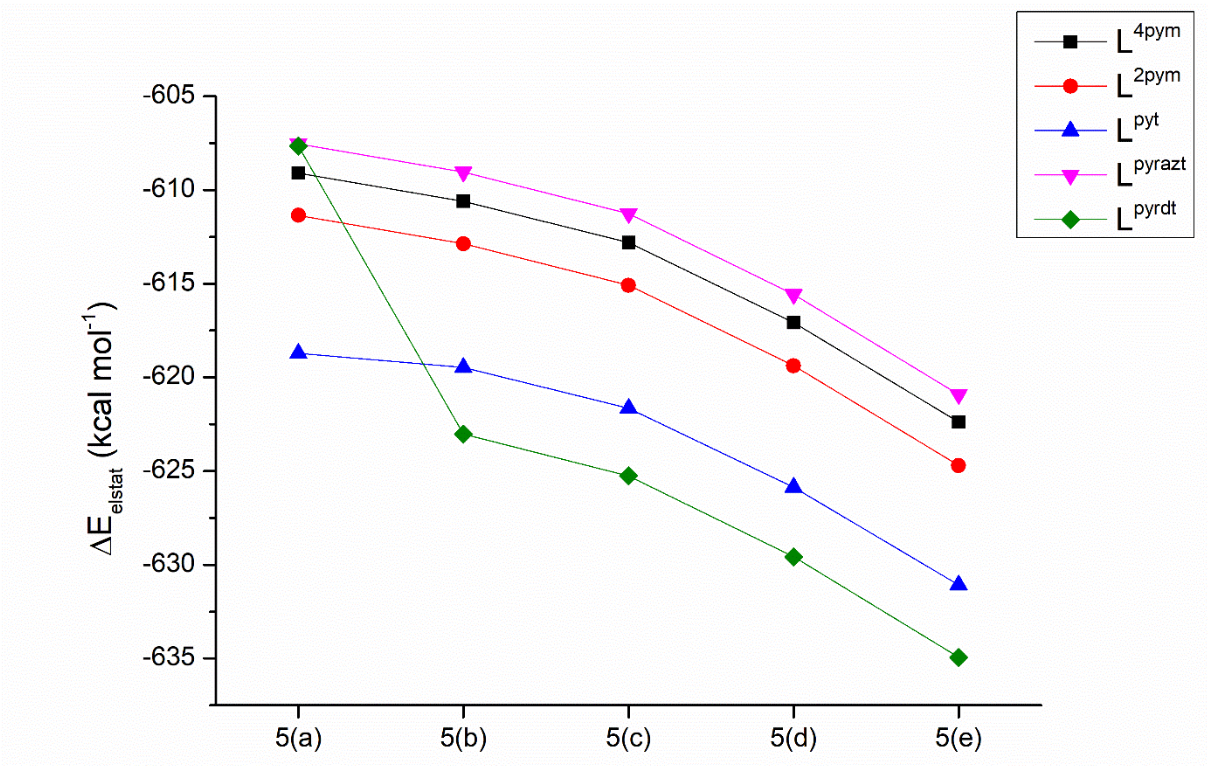

Figure S17. Trend of $\boldsymbol{\Delta E_{elstat}}$ energy contribution (kcal/mol) for each of the five *LS* [Fe($\boldsymbol{L^{azine}}$)$_2$(NCBH$_3$)$_2$] systems across the five sub fragmentations **5a-5e**.

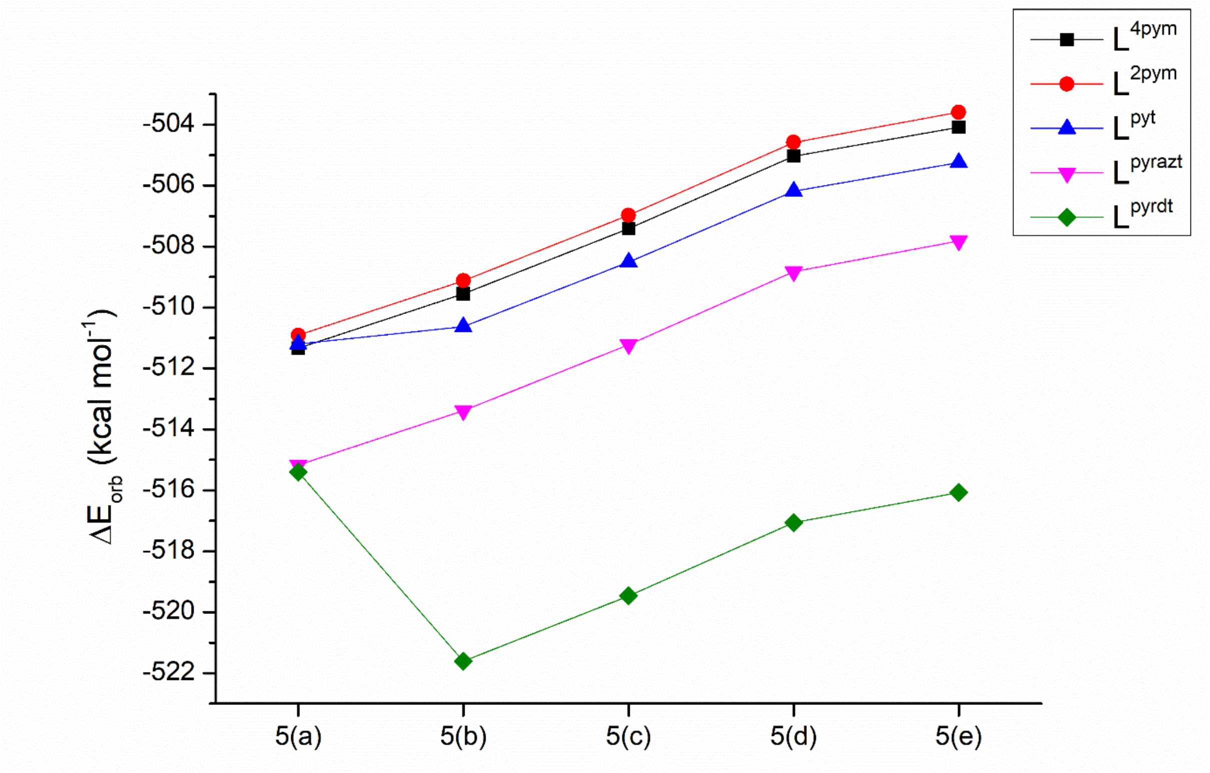

Figure S18. Trend of $\boldsymbol{\Delta E_{orb}}$ energy contribution (kcal/mol) for each of the five *LS* [Fe($\boldsymbol{L^{azine}}$)$_2$(NCBH$_3$)$_2$] systems across the five sub fragmentations **5a-5e**.

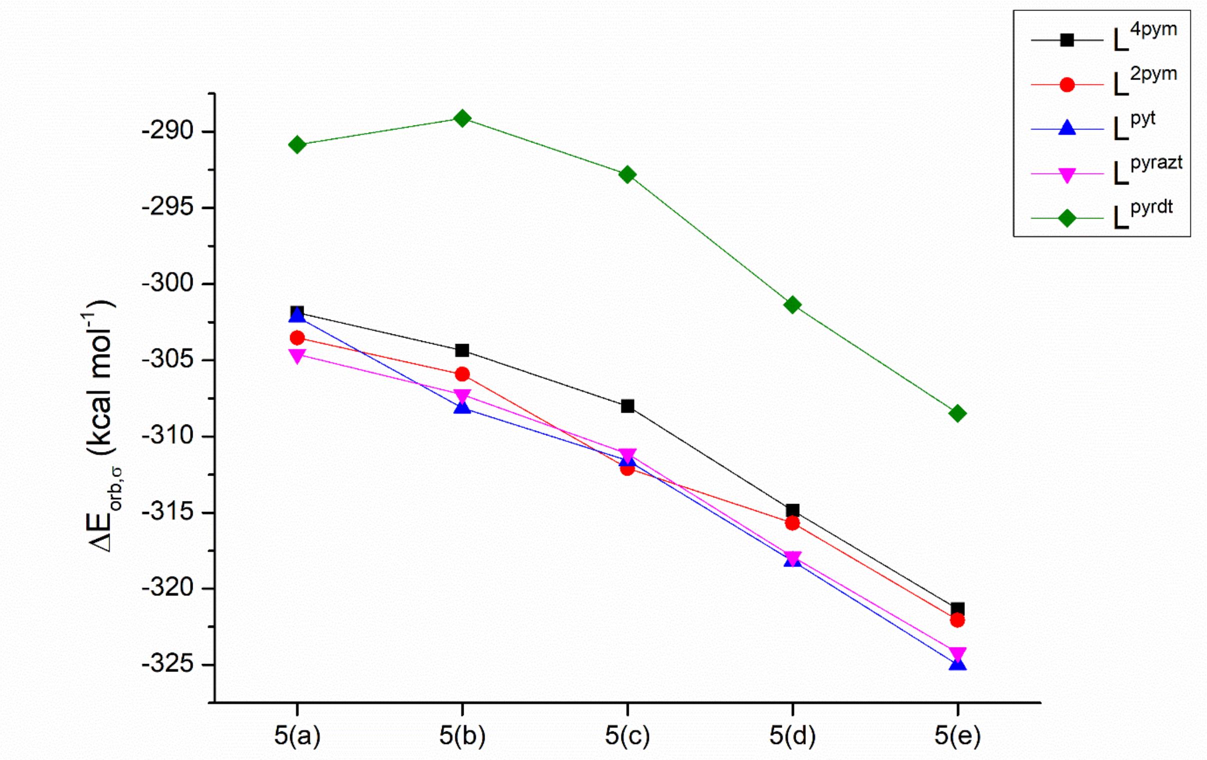

Figure S19. Trend of $\Delta E_{orb,\sigma}$ energy contribution (kcal/mol) for each of the five *LS* [Fe(L^{azine})$_2$($NCBH_3$)$_2$] systems across the five sub fragmentations **5a-5e**.

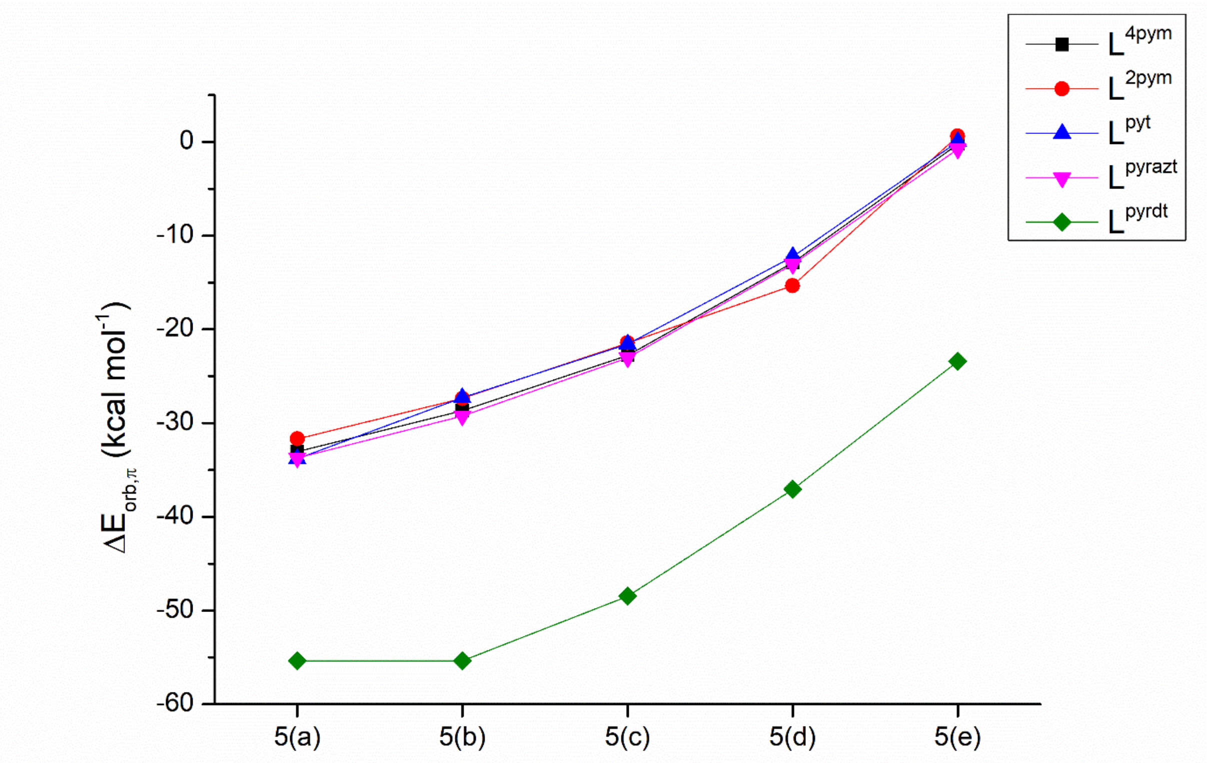

Figure S20. Trend of $\Delta E_{orb,\pi}$ energy contribution (kcal/mol) for each of the five *LS* [Fe(L^{azine})$_2$($NCBH_3$)$_2$] systems across the five sub fragmentations **5a-5e**.

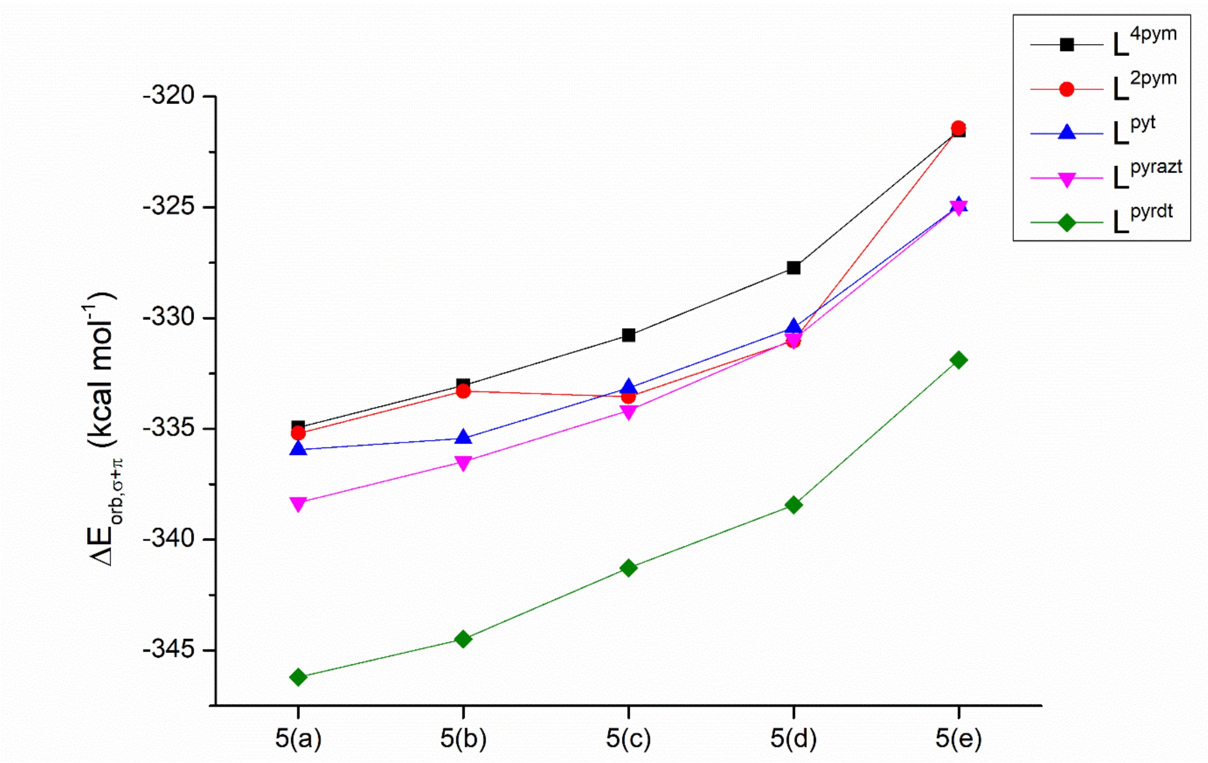

Figure S21. Trend of $\Delta E_{orb,\sigma+\pi}$ energy contribution (kcal/mol) for each of the five *LS* [Fe(L^{azine})$_2$(NCBH$_3$)$_2$] systems across the five sub fragmentations **5a-5e**.

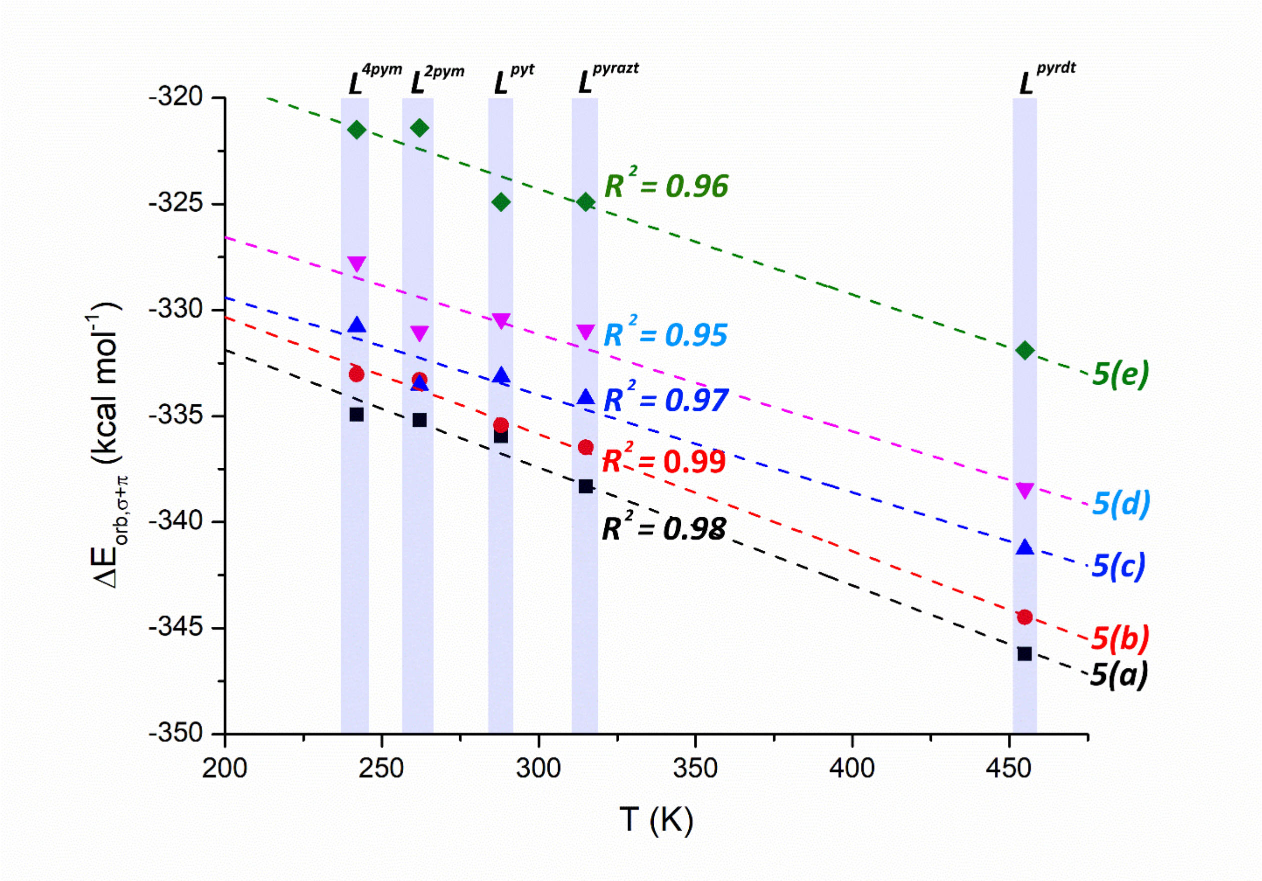

Figure S22. Correlation lines observed for $\Delta E_{orb,\sigma+\pi}$ energy contribution (kcal/mol) across the *LS* [Fe(L^{azine})$_2$(NCBH$_3$)$_2$] family in the five sub fragmentations **5a-5e**.

S3 – EDA-NOCV Results

S3.1 - Fragmentation 1- *LS* $Fe^{II}(L^{azine})_2(NCBH_3)_2$

Table S17. Summary EDA-NOCV results (kcal/mol) for fragmentation **(1)** for all the five *LS* $[Fe(L^{azine})_2(NCBH_3)_2]$ systems. Fragmentation **(1)** describes interaction between one of the axial coligands **$NCBH_3$** with the remaining **ML_5** system. First section (top) reports ΔE_{int} energy splitting; second section (middle) reports ΔE_{orb} energy splitting; third section (bottom) reports all the orbitalic interaction due to **ML_5** + **L** interaction.

	$L^{4pyrimidine}$	$L^{2pyrimidine}$	$L^{pyridine}$	$L^{pyrazine}$	$L^{pyridazine}$
ΔE_{int}	-108.1	-108.3	-103.4	-110.0	-105.0
ΔE_{Pauli}	106.9	106.9	107.2	107.0	106.5
ΔE_{elstat}	-147.2 (68.4%)	-147.5 (68.4%)	-143.6 (68.1%)	-148.8 (68.8%)	-143.7 (67.7%)
ΔE_{orb}	-63.5 (29.5%)	-63.5 (29.5%)	-62.7 (30.0%)	-64.0 (29.5%)	-63.4 (29.9%)
ΔE_{disp}	-4.2 (2.1%)	-4.2 (2.1%)	-4.3 (2.9%)	-4.3 (1.8%)	-4.4 (2.4%)
$\Delta E_{orb,\sigma}$	-36.0 (56.3%)	-36.0 (56.3%)	-35.6 (57.1%)	-36.4 (56.3%)	-36.3 (57.1%)
$\Delta E_{orb,\pi}$	-12.3 (19.4%)	-12.3 (19.4%)	-12.3 (19.6%)	-12.2 (19.4%)	-11.9 (19.4%)
$\Delta E_{orb,\sigma+\pi}$	-48.3 (75.7%)	-48.3 (75.7%)	-47.9 (76.7%)	-48.6 (75.7%)	-48.2 (76.5%)
$\Delta E_{orb,pol}$	-7.9 (13.2%)	-8.4 (13.4%)	-9.7 (13.3%)	-8.5 (13.2%)	-7.8 (12.4%)
$\Delta E_{orb,rest}$	-7.3 (11.1%)	-6.8 (10.9%)	-5.1 (10.0%)	-6.9 (11.1%)	-7.4 (11.1%)
$\Delta E_{orb,dz2}$	-36.0	-36.0	-35.6	-36.4	-36.3
$\Delta E_{orb,dzx}$	-6.3	-6.3	-6.3	-6.3	-6.1
$\Delta E_{orb,dzy}$	-6.0	-6.0	-6.0	-6.0	-5.8

Figure S23. Plot of the deformation densities $\Delta\rho_{(i)}$ in fragmentation **(1)** with corresponding energy contribution to the total orbital term $\boldsymbol{\Delta E}$ (given in kcal/mol) of the [TM]←ligand σ-donation, the [TM]→ligand π-backdonation in reference complex *LS* [Fe($\boldsymbol{L}^{pyridine}$)$_2$(NCBH$_3$)$_2$]. The direction of the charge flow is yellow → turquoise. The eigenvalues |*v*| indicate the relative size of the charge flow. Deformation densities describing bond interaction are reported using a cut-off on $\Delta\rho_{(i)}$ of 0.003 as this produced the clearest image; please note that the EDA-NOCV analysis are performed by applying the default cutoffs on NOCVs energies (0.5 kcal/mol) and individual SFO contribution (0.001).

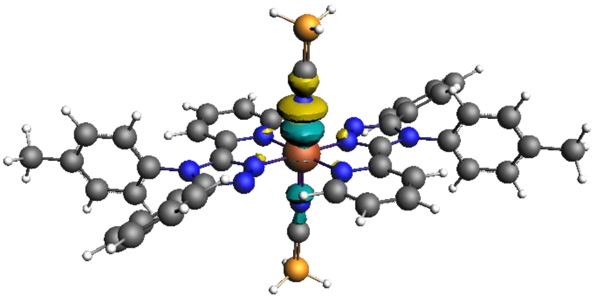

Δρ(1) $\Delta E_1 = -35.6$, $|v_1| = 0.57$ ($d_{[TM]}$←ligand σ)

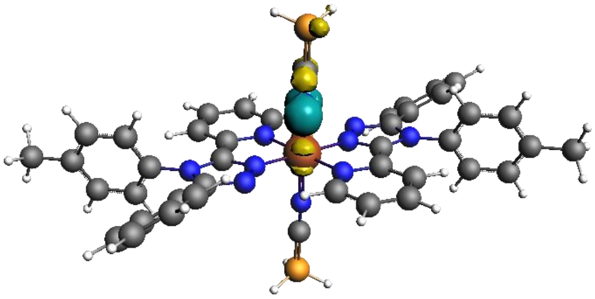

Δρ(2) $\Delta E_2 = -6.3$, $|v_2| = 0.25$ ($d_{[TM]}$→ligand π)

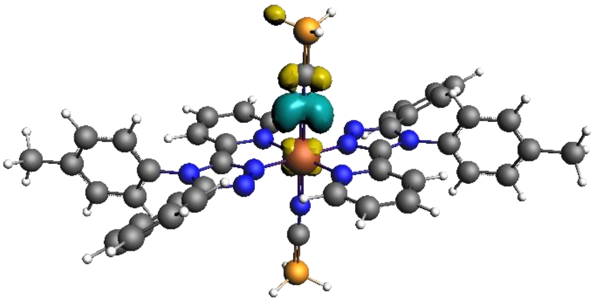

Δρ(3) $\Delta E_3 = -6.0$, $|v_3| = 0.24$ ($d_{[TM]}$→ligand π)

S3.2 - Fragmentation 2- *LS* Fe^{II}(L^{azine})$_2$($NCBH_3$)$_2$

Table S18. Summary EDA-NOCV results (kcal/mol) for fragmentation **(2)** for all the five *LS* [Fe(L^{azine})$_2$($NCBH_3$)$_2$] systems. Fragmentation **(2)** describes interaction between one of the equatorial ligands L^{azine} with the remaining **ML_5** system. First section (top) reports ΔE_{int} energy splitting; second section (middle) reports ΔE_{orb} energy splitting; third section (bottom) reports all the orbital interaction due to **ML_4** + **L_2** interaction.

	$L^{4pyrimidine}$	$L^{2pyrimidine}$	$L^{pyridine}$	$L^{pyrazine}$	$L^{pyridazine}$
ΔE_{int}	-89.4	-91.0	-90.3	-91.2	-83.4
ΔE_{Pauli}	174.2	175.6	174.1	178.4	168.7
ΔE_{elstat}	-148.2 (56.3%)	-150.2 (56.4%)	-148.7 (56.4%)	-151.3 (55.9%)	-138.3 (55.0%)
ΔE_{orb}	-101.3 (38.4%)	-102.4 (38.3%)	-101.5 (38.3%)	-104.2 (38.5%)	-100.8 (39.8%)
ΔE_{disp}	-14.0 (5.3%)	-14.0 (5.3%)	-14.3 (5.3%)	-14.2 (5.6%)	-12.9 (5.2%)
$\Delta E_{orb,\sigma}$	-62.6 (62.4%)	-62.9 (61.8%)	-62.2 (60.8%)	-63.7 (61.3%)	-64.3 (63.6%)
$\Delta E_{orb,\pi}$	-22.6 (22.3%)	-22.9 (22.5%)	-22.4 (21.6%)	-23.5 (22.1%)	-24.7 (24.7%)
$\Delta E_{orb,\sigma+\pi}$	-85.2 (84.7%)	-85.7 (84.3%)	-84.6 (82.4%)	-87.2 (83.4%)	-89.0 (88.3%)
$\Delta E_{orb,pol}$	11.1 (10.3%)	12.0 (11.1%)	12.1 (12.9%)	15.3 (7.8%)	7.1 (7.0%)
$\Delta E_{orb,rest}$	-5.0 (5.0%)	-4.7 (4.6%)	-4.8 (4.7%)	-4.7 (4.8%)	-4.7 (4.7%)
$\Delta E_{orb,dz2}$	-22.8	-22.8	-22.7	-23.0	-22.8
$\Delta E_{orb,dx2-y2}$	-39.9	-40.1	-39.5	-40.7	-41.5
$\Delta E_{orb,dzx}$	-11.0	-10.8	-10.9	-11.4	-12.4
$\Delta E_{orb,dzy}$	-6.1	-6.5	-6.1	-6.5	-6.6
$\Delta E_{orb,dxy}$	-5.6	-5.5	-5.4	-5.5	-5.7

Figure S24. Plot of the deformation densities $\Delta\rho_{(i)}$ in fragmentation **(2)** with corresponding energy contribution to the total orbital term $\boldsymbol{\Delta E}$ (given in kcal/mol) of the [TM]←ligand σ-donation, the [TM]→ligand π-backdonation in reference complex *LS* [Fe($\boldsymbol{L}^{pyridine}$)$_2$($NCBH_3$)$_2$]. The direction of the charge flow is yellow → turquoise. The eigenvalues $|v|$ indicate the relative size of the charge flow. Deformation densities describing bond interaction are reported using a cut-off on $\Delta\rho_{(i)}$ of 0.003 as this produced the clearest image; please note that the EDA-NOCV analysis are performed by applying the default cutoffs on NOCVs energies (0.5 kcal/mol) and individual SFO contribution (0.001).

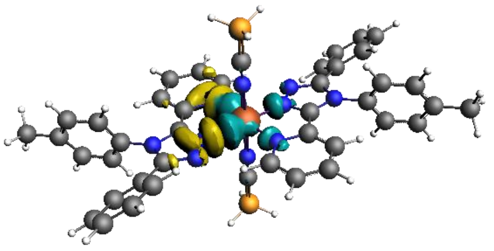

$\Delta\rho_{(1)}$ ΔE_1 = –-39.5, $|v_1|$ = 0.69 ($d_{[TM]}$←ligand σ)

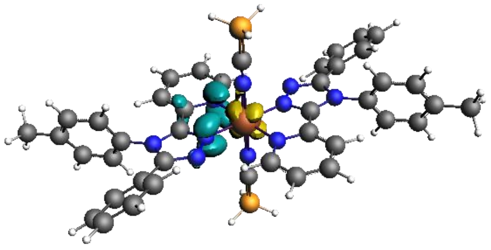

$\Delta\rho_{(2)}$ ΔE_2 = –10.9, $|v_2|$ = 0.51 ($d_{[TM]}$→ligand π)

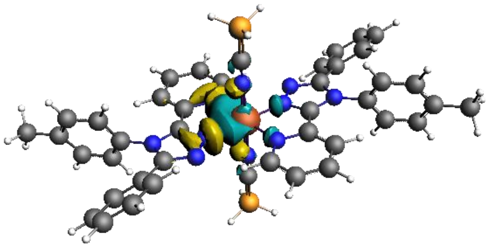

$\Delta\rho_{(3)}$ ΔE_3 = –22.7, $|v_3|$ = 0.45 ($d_{[TM]}$←ligand σ)

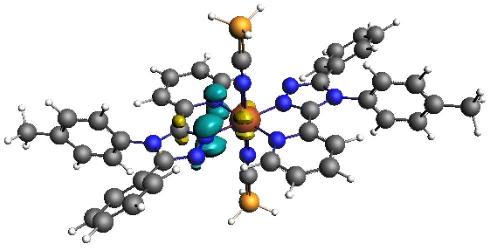

$\Delta\rho_{(4)}$ ΔE_4 = –6.1, $|v_4|$ = 0.30 ($d_{[TM]}$→ligand π)

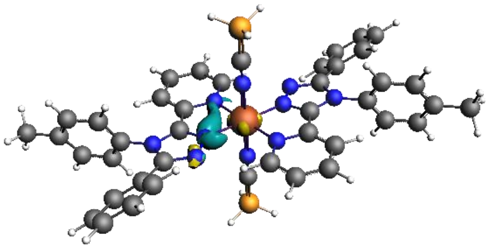

$\Delta\rho_{(5)}$ ΔE_3 = –5.4, $|v_3|$ = 0.19 ($d_{[TM]}$→ligand π)

S3.3 - Fragmentation 3- *LS* $Fe^{II}(L^{azine})_2(NCBH_3)_2$

Table S19. Summary EDA-NOCV results (kcal/mol) for fragmentation **(3)** for all the five *LS* [Fe($\boldsymbol{L}^{azine}$)$_2$(NCBH$_3$)$_2$] systems. Fragmentation **(3)** describes interaction between one of both axial coligands **2xNCBH$_3$** with the remaining **ML$_4$** system. First section (top) reports ΔE_{int} energy splitting; second section (middle) reports ΔE_{orb} energy splitting; third section (bottom) reports all the orbitalic interaction due to **ML$_4$** + **L$_2$** interaction.

	$L^{4pyrimidine}$	$L^{2pyrimidine}$	$L^{pyridine}$	$L^{pyrazine}$	$L^{pyridazine}$
ΔE_{int}	-345.9	-346.1	-335.9	-349.7	-337.7
ΔE_{Pauli}	176.1	176.4	170.6	177.6	179.4
ΔE_{elstat}	-377.4 (72.2%)	-378.0 (72.3%)	-370.3 (72.1%)	-380.7 (72.1%)	-370.8 (71.8%)
ΔE_{orb}	-132.3 (25.3%)	-132.4 (25.2%)	-129.9 (25.3%)	-134.4 (25.7%)	-134.2 (25.9%)
ΔE_{disp}	-12.2 (2.5%)	-12.2 (2.5%)	-12.3 (2.6%)	-12.3 (2.2%)	-12.1 (2.3%)
$\Delta E_{orb,\sigma}$	-72.4 (54.8%)	-72.3 (54.6%)	-71.3 (54.6%)	-74.2 (55.2%)	-75.0 (55.9%)
$\Delta E_{orb,\pi}$	-23.3 (17.4%)	-23.6 (17.8%)	-23.8 (18.3%)	-23.5 (17.5%)	-23.1 (17.2%)
$\Delta E_{orb,\sigma+\pi}$	-95.7 (72.2%)	-95.9 (72.4%)	-95.0 (72.9%)	-97.7 (72.7%)	-98.1 (72.1%)
$\Delta E_{orb,pol}$	28.3 0 (21.6%)	28.3 (21.6%)	27.4 (21.1%)	27.6 (20.8%)	27.1 (21.2%)
$\Delta E_{orb,rest}$	8.2 (6.2%)	8.2 (6.2%)	-7.6 (5.8%)	-8.7 (6.5%)	-9.0 (6.7%)
$\Delta E_{orb,dz2}$	-58.4	-58.4	-57.6	-60.0	-61.1
$\Delta E_{orb,dzx}$	-11.3	-11.8	-11.8	-11.6	-11.5
$\Delta E_{orb,dzy}$	-12.0	-11.8	-12.0	-11.9	-11.5
$\Delta E_{orb,pz}$	-14.0	-13.9	-13.7	-14.2	-14.0

Figure S25. Plot of the deformation densities $\Delta\rho_{(i)}$ in fragmentation **(4)** with corresponding energy contribution to the total orbital term ***ΔE*** (given in kcal/mol) of the [TM]←ligand σ-donation, the [TM]→ligand π-backdonation in reference complex *LS* [Fe($L^{pyridine}$)$_2$($NCBH_3$)$_2$]. The direction of the charge flow is yellow → turquoise. The eigenvalues |*v*| indicate the relative size of the charge flow. Deformation densities describing bond interaction are reported using a cut-off on $\Delta\rho_{(i)}$ of 0.003 as this produced the clearest image; please note that the EDA-NOCV analysis are performed by applying the default cutoffs on NOCVs energies (0.5 kcal/mol) and individual SFO contribution (0.001).

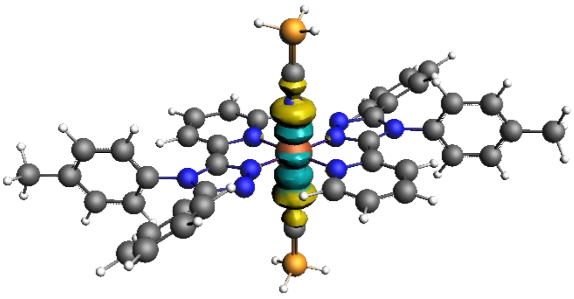

$\Delta\rho_{(1)}$ $\Delta E_1 = -57.6$, $|v_1| = 0.72$ ($d_{[TM]}$←ligand σ)

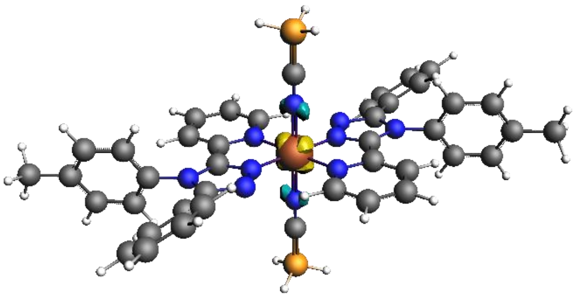

$\Delta\rho_{(3)}$ $\Delta E_3 = -11.8$, $|v_3| = 0.37$ ($d_{[TM]}$→ligand π)

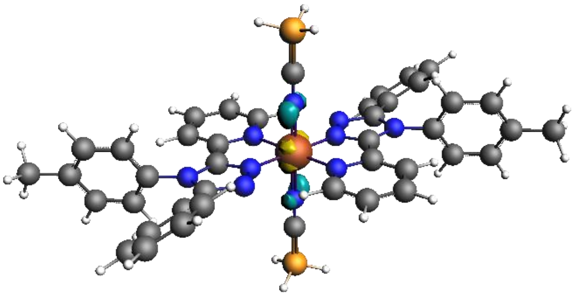

$\Delta\rho_{(2)}$ $\Delta E_2 = -12.0$, $|v_2| = 0.35$ ($d_{[TM]}$→ligand π)

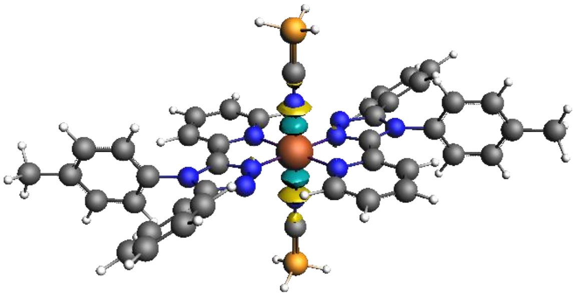

$\Delta\rho_{(4)}$ $\Delta E_4 = -13.7$, $|v_4| = 0.36$ ($d_{[TM]}$←ligand σ)

S3.4 - Fragmentation 4 - *LS* Fe^{II}(L^{azine})$_2$($NCBH_3$)$_2$

Table S20. Summary EDA-NOCV results (kcal/mol) for fragmentation **(4)** for all the five *LS* [Fe($\boldsymbol{L}^{azine}$)$_2$($NCBH_3$)$_2$] systems. Fragmentation **(4)** describes interaction between one of both equatorial ligands **2x$\boldsymbol{L}^{azine}$** with the remaining **ML_4** system. First section (top) reports **ΔE_{int}** energy splitting; second section (middle) reports **ΔE_{orb}** energy splitting; third section (bottom) reports all the orbitalic interaction due to **ML_2** + **L_4** interaction.

	$L^{4pyrimidine}$	$L^{2pyrimidine}$	$L^{pyridine}$	$L^{pyrazine}$	$L^{pyridazine}$
ΔE_{int}	-228.1	-228.3	-233.0	-229.7	-238.8
ΔE_{Pauli}	260.7	262.6	257.5	265.3	270.7
ΔE_{elstat}	-250.9 (51.4%)	-252.3 (51.3%)	-252.3 (51.3%)	-253.1 (51.1%)	-259.3 (40.1%)
ΔE_{orb}	-218.4 (44.7%)	-219.1 (44.6%)	-218.3 (44.4%)	-222.2 (44.8%)	-231.0 (45.4%)
ΔE_{disp}	-19.4 (3.9%)	-19.5 (4.1%)	-19.7 (4.3%)	-19.7 (4.1%)	-19.2 (4.6%)
$\Delta E_{orb,\sigma}$	-134.9 (61.8%)	-134.6 (61.5%)	-136.0 (62.4%)	-139.4 (62.7%)	-141.9 (61.4%)
$\Delta E_{orb,\pi}$	-48.8 (22.3%)	-49.6 (22.6%)	-49.2 (22.6%)	-46.6 (21.0%)	-51.6 (22.3%)
$\Delta E_{orb,\sigma+\pi}$	-183.6 (84.1%)	-184.2 (84.1%)	-185.2 (85.0%)	-186.0 (83.7%)	-193.5 (83.7%)
$\Delta E_{orb,pol}$	-19.6 (9.0%)	-19.8 (9.0%)	-18.1 (8.3%)	-20.7 (9.3%)	-21.4 (9.3%)
$\Delta E_{orb,rest}$	-15.2 (6.9%)	-15.1 (6.9%)	-15.0 (6.9%)	-15.5 (7.0%)	-16.1 (7.0%)
$\Delta E_{orb,dz2}$	-30.1	-29.2	-29.0	-33.4	-33.0
$\Delta E_{orb,dx2-y2}$	-82.8	-83.6	-84.2	-84.6	-87.2
$\Delta E_{orb,dzx}$	-24.5	-24.0	-25.5	-21.5	-26.8
$\Delta E_{orb,dzy}$	-12.2	-11.9	-11.6	-12.1	-11.3
$\Delta E_{orb,dxy}$	-12.1	-11.9	-11.6	-12.1	-11.3
$\Delta E_{orb,px}$	-12.0	-11.9	-11.9	-11.5	-11.2
$\Delta E_{orb,py}$	-10.1	-9.9	-10.9	-9.9	-10.5

Figure S26. Plot of the deformation densities $\Delta\rho_{(i)}$ in fragmentation **(4)** with corresponding energy contribution to the total orbital term $\boldsymbol{\Delta E}$ (given in kcal/mol) of the [TM]←ligand σ-donation, the [TM]→ligand π-backdonation in reference complex *LS* [Fe($\boldsymbol{L}^{pyridine}$)$_2$($NCBH_3$)$_2$]. The direction of the charge flow is yellow → turquoise. The eigenvalues $|v|$ indicate the relative size of the charge flow. Deformation densities describing bond interaction are reported using a cut-off on $\Delta\rho_{(i)}$ of 0.003 as this produced the clearest image; please note that the EDA-NOCV analysis are performed by applying the default cutoffs on NOCVs energies (0.5 kcal/mol) and individual SFO contribution (0.001).

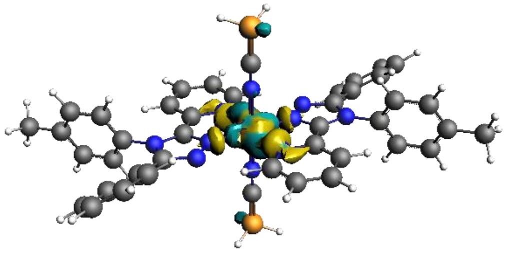

$\boldsymbol{\Delta\rho_{(1)}}$ ΔE_1 = –84.2, $|v_1|$ = 1.19 ($d_{[TM]}$←ligand σ)

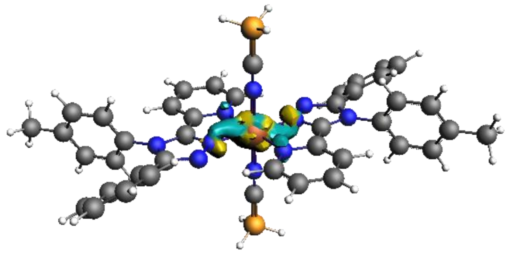

$\boldsymbol{\Delta\rho_{(2)}}$ ΔE_2 = –25.5, $|v_2|$ = 0.70 ($d_{[TM]}$→ligand π)

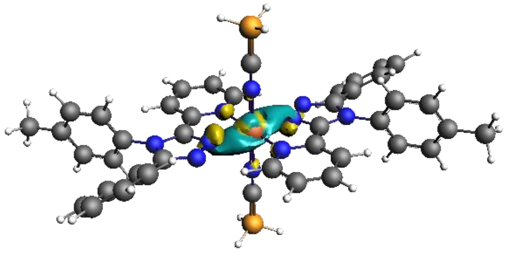

$\boldsymbol{\Delta\rho_{(3)}}$ ΔE_3 = –29.0, $|v_3|$ = 0.54 ($d_{[TM]}$←ligand σ)

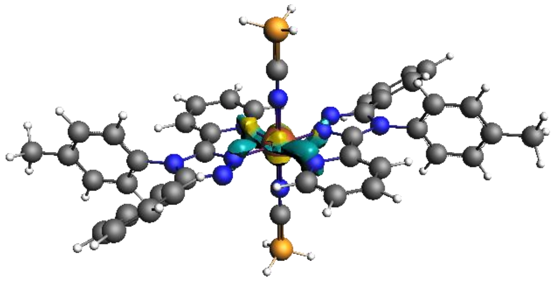

$\boldsymbol{\Delta\rho_{(4)}}$ ΔE_4 = –11.6, $|v_4|$ = 0.36 ($d_{[TM]}$→ligand π)

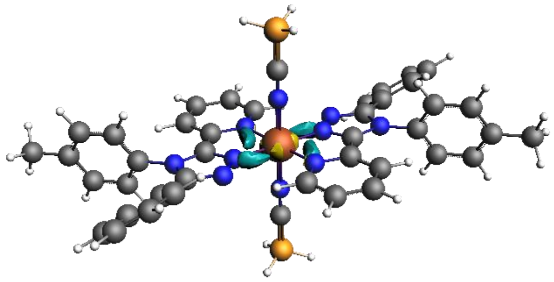

$\boldsymbol{\Delta\rho_{(5)}}$ ΔE_3 = –11.6, $|v_3|$ = 0.27 ($d_{[TM]}$→ligand π)

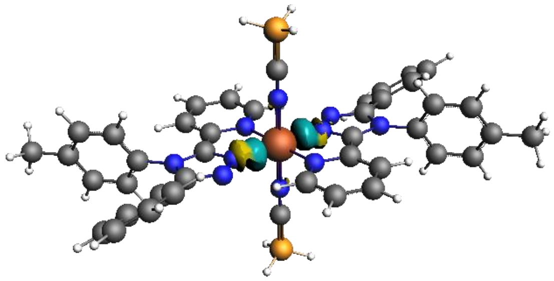

$\boldsymbol{\Delta\rho_{(6)}}$ ΔE_2 = –11.9, $|v_2|$ = 0.22 ($d_{[TM]}$←ligand σ)

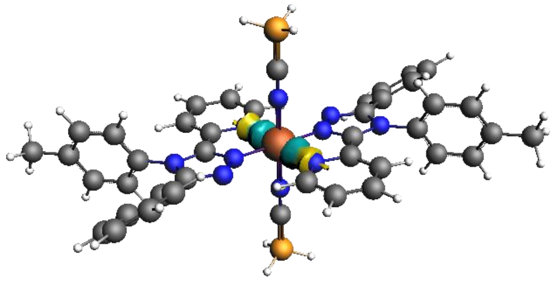

Δρ(7) $\Delta E_3 = -10.9$, $|v_3| = 0.21$ ($d_{[TM]}$←ligand σ)

S3.5 - Fragmentation 5b/5e - *LS* Fe^{II}(L^{azine})$_2$(NCBH$_3$)$_2$

Table S21. EDA-NOCV results (kcal/mol) reported for fragmentation **5b-5e** for all the five *LS* Fe^{II}(L^{azine})$_2$(NCBH$_3$)$_2$ systems. First section (top) reports ***ΔE$_{int}$*** energy splitting **5e**; second section (middle) reports ***ΔE$_{orb}$*** energy splitting; third section (bottom) reports all the nine orbitalic interaction due to **M** - **L$_6$** interaction **5b**.

	$L^{4pyrimidine}$	$L^{2pyrimidine}$	$L^{pyridine}$	$L^{pyrazine}$	$L^{pyridazine}$
ΔE_{int}	-505.1	-505.5	-516.0	-503.9	-522.4
ΔE_{Pauli}	631.0	632.3	630.0	634.5	638.0
ΔE_{elstat}	-622.4 (54.9%)	-624.7 (55.0%)	-631.1 (55.3%)	-620.9 (54.6%)	-634.9 (54.7%)
ΔE_{orb}	-504.1 (44.5%)	-503.6 (44.4%)	-505.2 (44.1%)	-507.8 (44.8%)	-516.1 (44.4%)
ΔE_{disp}	-9.6 (0.6%)	-9.5 (0.6%)	-9.7 (0.6%)	-9.6 (0.6%)	-9.4 (0.9%)
$\Delta E_{orb,\sigma}$	-304.3 (59.6%)	-305.9 (60.1%)	-308.1 (60.3%)	-307.3 (60.4%)	-289.1 (56.4%)
$\Delta E_{orb,\pi}$	-28.7 (5.7%)	-27.4 (5.3%)	-27.3 (5.3%)	-29.2 (4.7%)	-55.4 (8.8%)
$\Delta E_{orb,\sigma+\pi}$	-333.0 (65.3%)	-333.3 (65.4%)	-335.4 (65.6%)	-336.5 (65.1%)	-344.5 (65.2%)
$\Delta E_{orb,pol}$	-146.5 (28.6%)	-145.8 (28.7%)	-145.2 (28.4%)	-146.9 (24.1%)	-146.6 (23.6%)
$\Delta E_{orb,rest}$	-30.6 (5.9%)	-33.5 (5.9%)	-31.6 (6.0%)	-30.5 (5.8%)	-31.3 (4.9%)
$\Delta E_{orb,dz2}$	-113.8	-113.4	-112.8	-112.5	-102.2
$\Delta E_{orb,dx2-y2}$	-116.3	-117.7	-120.4	-119.3	-110.3
$\Delta E_{orb,dzx}$	-3.4	-2.6	-1.3	-3.2	-29.2
$\Delta E_{orb,dzy}$	-10.8	-10.1	-11.2	-11.5	-10.9
$\Delta E_{orb,dxy}$	-14.6	-14.7	-14.9	-14.6	-15.3
$\Delta E_{orb,s}$	-23.4	-23.2	-23.8	-23.6	-23.3
$\Delta E_{orb,pz}$	-18.8	-19.4	-19.3	-19.8	-21.3
$\Delta E_{orb,px}$	-18.6	-19.0	-19.3	-19.1	-19.0
$\Delta E_{orb,py}$	-13.4	-13.3	-13.5	-12.9	-13.1

Figure S27. Plot of the deformation densities $\Delta\rho_{(i)}$ in fragmentation **5b** with corresponding energy contribution to the total orbital term ***ΔE*** (given in kcal/mol) of the [TM]←ligand σ-donation, the [TM]→ligand π-backdonation and polarization in reference complex *LS* [Fe(***L**^pyridine^*)$_2$(NCBH$_3$)$_2$]. The direction of the charge flow is yellow → turquoise. The eigenvalues |*v*| indicate the relative size of the charge flow. Figures are reported using until $|v_i|$ = 0.1, with cut-off on $\Delta\rho_{(i)}$=0.003 as this produced the clearest image; please note that the EDA-NOCV analysis are performed by applying the default cutoffs on NOCVs energies (0.5 kcal/mol) and individual SFO contribution (0.001).

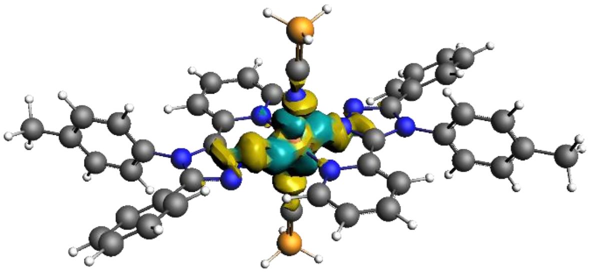

Δρ(1) ΔE_1 = –112.8, $|v_1|$ = 0.98 ($d_{[TM]}$←ligand σ)

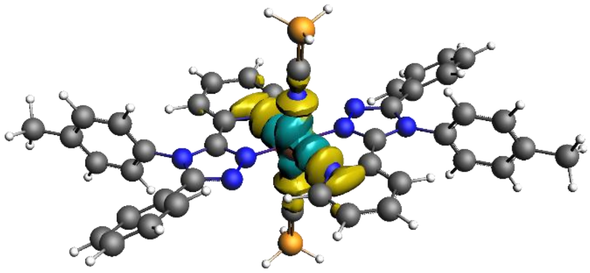

Δρ(2) ΔE_2 = –120.4, $|v_2|$ = 0.93 ($d_{[TM]}$←ligand σ)

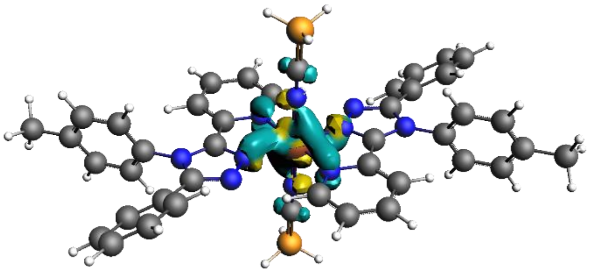

Δρ(3) ΔE_3 = –1.3, $|v_3|$ = 0.82 ($d_{[TM]}$→ligand π)

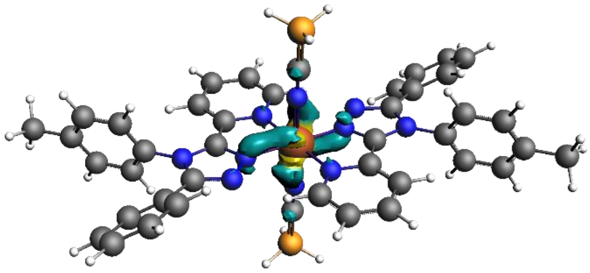

Δρ(4) ΔE_4 = –11.2, $|v_4|$ = 0.56 ($d_{[TM]}$→ligand π)

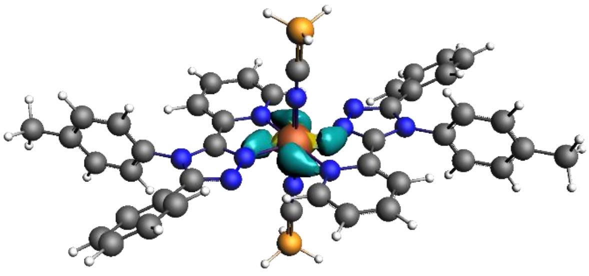

Δρ(5) ΔE_5 = –14.9, $|v_4|$ = 0.33 ($d_{[TM]}$→ligand π)

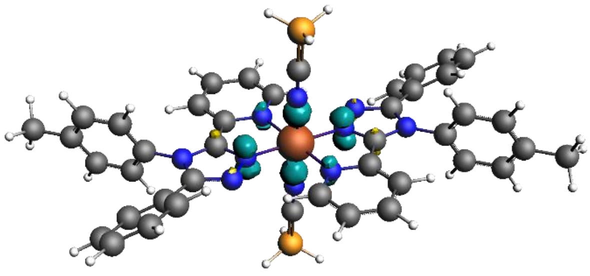

Δρ(6) ΔE_6 = –24.1, $|v_6|$ = 0.32 (pol)

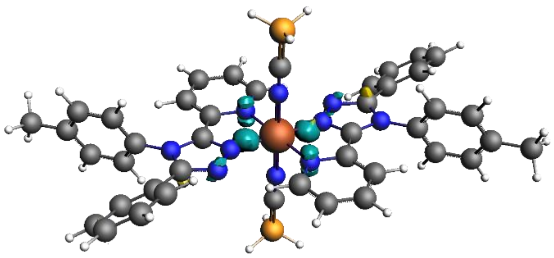

$\Delta\rho_{(7)}$ $\Delta E_7 = -20.2$, $|\nu_7| = 0.30$ (pol)

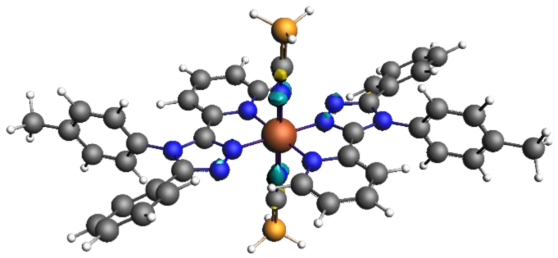

$\Delta\rho_{(8)}$ $\Delta E_8 = -16.1$, $|\nu_8| = 0.27$ (pol)

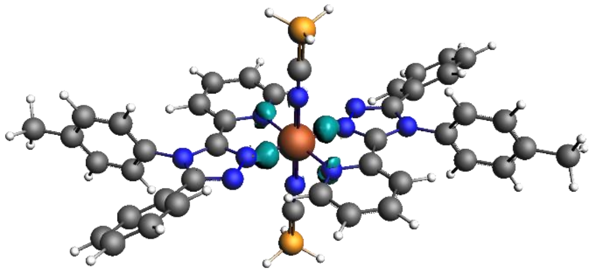

$\Delta\rho_{(9)}$ $\Delta E_9 = -19.3$, $|\nu_9| = 0.26$ ($p_{[TM]} \leftarrow$ ligand σ)

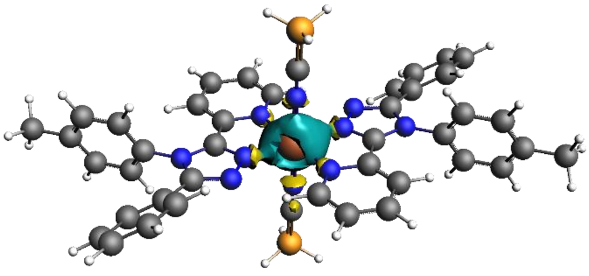

$\Delta\rho_{(10)}$ $\Delta E_{10} = -23.8$, $|\nu_{10}| = 0.24$ ($s_{[TM]} \leftarrow$ ligand σ)

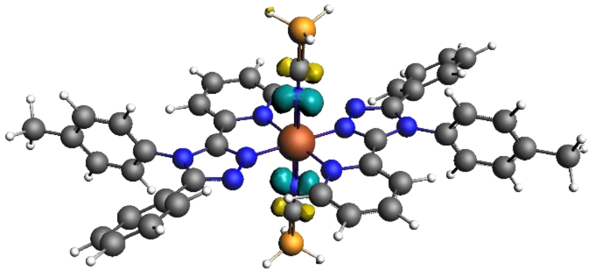

$\Delta\rho_{(11)}$ $\Delta E_{11} = -15.5$, $|\nu_{11}| = 0.23$ (pol)

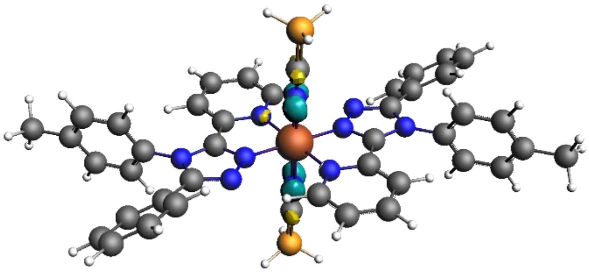

$\Delta\rho_{(12)}$ $\Delta E_{12} = -17.0$, $|\nu_{12}| = 0.22$ (pol)

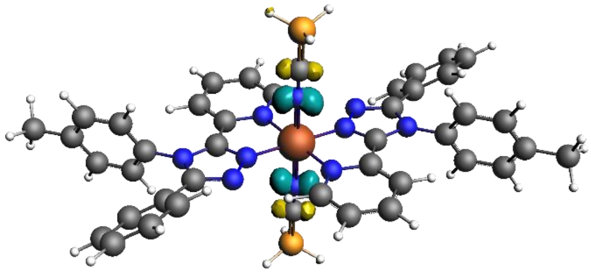

$\Delta\rho_{(13)}$ $\Delta E_{13} = -16.0$, $|\nu_{13}| = 0.22$ (pol)

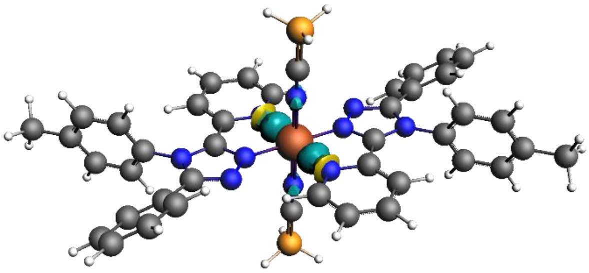

$\Delta\rho_{(14)}$ $\Delta E_{14} = -17.0$, $|\nu_{14}| = 0.21$ ($p_{[TM]} \leftarrow$ ligand σ)

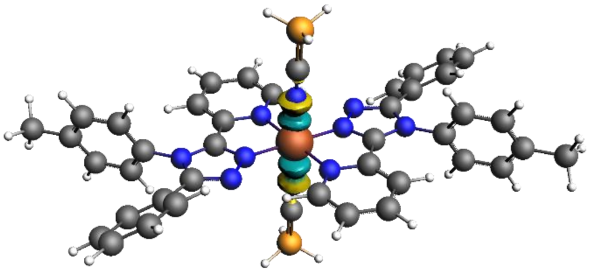

$\Delta\rho_{(15)}$ $\Delta E_{15} = -13.5$, $|\nu_{15}| = 0.17$ ($p_{[TM]} \leftarrow$ ligand σ)

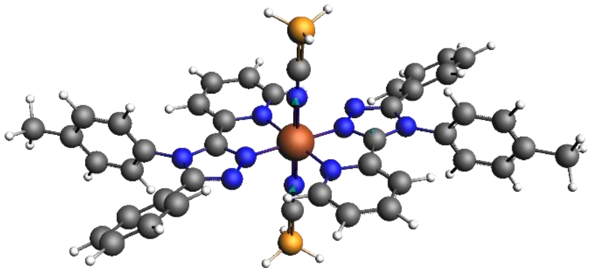

$\Delta\rho_{(16)}$ $\Delta E_{16} = -6.3$, $|\nu_{16}| = 0.15$ (pol)

S3.6 - Fragmentation 5b/5e - *HS* $Fe^{II}(L^{azine})_2(NCBH_3)_2$

Table S22. EDA-NOCV results (kcal/mol) reported for fragmentation **5b-5e** for all the five *HS* $Fe(\boldsymbol{L}^{azine})_2(NCBH_3)_2$. First section (top) reports $\boldsymbol{\Delta E_{int}}$ energy splitting **5e**; second section (middle) reports $\boldsymbol{\Delta E_{orb}}$ energy splitting; third section (bottom) reports all the nine orbitalic interaction due to **M** - $\mathbf{L_6}$ interaction **5b**.

	$L^{4pyrimidine}$	$L^{2pyrimidine}$	$L^{pyridine}$	$L^{pyrazine}$	$L^{pyridazine}$
ΔE_{int}	-370.29	-369.34	-380.45	-367.96	-385.49
ΔE_{Pauli}	544.31	544.33	543.52	547.15	539.3
ΔE_{elstat}	-573.4 (62.7%)	-574.3 (62.9%)	-582.4 (63.0%)	-571.8 (62.5%)	-583.5 (63.10%)
ΔE_{orb}	-327.9 (35.9%)	-326.2 (35.7%)	-328.1 (35.5%)	-329.9 (36.1%)	-328.4 (35.5%)
ΔE_{disp}	-13.3 (1.48%)	-13.2 (1.48%)	-13.4 (1.48%)	-13.4 (1.49%)	-12.9 (1.42%)
$\Delta E_{orb,\sigma}$ [α+β]	-145.5 (44.61%)	-145.4 (44.80%)	-145.6 (44.60%)	-149.4 (45.49%)	-155.6 (47.68%)
$\Delta E_{orb,\pi}$ [α+β]	-3.4 (1.04%)	-3.4 (1.06%)	-3.5 (1.06%)	-3.34 (1.02%)	-3.2 (0.98%)
$\Delta E_{orb,\sigma+\pi}$ [α+β]	-148.9 (45.65%)	-148.8 (45.86%)	-149.1 (45.66%)	-152.7 (46.51%)	-158.8 (48.66%)
$\Delta E_{orb,pol}$	-178.3 (54.66%)	-176.7 (54.45%)	-178.4 (54.64%)	-176.6 (53.79%)	-168.6 (51.66%)
$\Delta E_{orb,rest}$	-42.8 (13.12%)	-46.7 (14.39%)	-43.3 (13.27%)	-43.5 (13.26%)	-42.8 (13.11%)
$\Delta E_{orb,dz2}$ [α+β]	-34.1	-33.8	-33.4	-34.8	-34.6
$\Delta E_{orb,dx2-y2}$ [α+β]	-30.7	-30.5	-31.0	-31.4	-31.7
$\Delta E_{orb,dzx}$ [α+β]	-	-	-	-	-
$\Delta E_{orb,dzy}$ [α+β]	-	-	-	-	-

**Δ*E*$_{orb,dxy}$** [α+β]	-3.4	-3.4	-3.5	-3.3	-3.2
**Δ*E*$_{orb,s}$** [α+β]	-27.1	-27.1	-25.9	-27.0	-29.6
**Δ*E*$_{orb,pz}$** [α+β]	-21.2	-20.1	-21.4	-21.7	-23.1
**Δ*E*$_{orb,px}$** [α+β]	-18.2	-17.6	-18.1	-18.9	-19.5
**Δ*E*$_{orb,py}$** [α+β]	-15.8	-15.8	-15.8	-15.8	-15.8
**Δ*E*$_{orb,dz2}$** [α]	-	-	-	-	-
**Δ*E*$_{orb,dx2-y2}$** [α]	-	-	-	-	-
**Δ*E*$_{orb,dzx}$** [α]	-	-	-	-	-
**Δ*E*$_{orb,dzy}$** [α]	-	-	-	-	-
**Δ*E*$_{orb,dxy}$** [α]	-3.4	-3.4	-3.5	-3.3	-3.2
**Δ*E*$_{orb,s}$** [α]	-13.5	-13.5	-12.4	-13.2	-15.4
**Δ*E*$_{orb,pz}$** [α]	-11.3	-11	-11.5	-11.7	-12.0
**Δ*E*$_{orb,px}$** [α]	-9.9	-9.2	-9.0	-9.8	-9.4
**Δ*E*$_{orb,py}$** [α]	-6.8	-8.9	-8.3	-8.5	-9.3
**Δ*E*$_{orb,pol}$** [α]	-93.1	-91.4	-93.3	-93.2	-90.8
**Δ*E*$_{orb,rest}$** [α]	-21.5	-23.5	-21.8	-22	-21.8
**Δ*E*$_{orb,dz2}$** [β]	-34.1	-33.8	-33.4	-34.8	-34.6
**Δ*E*$_{orb,dx2-y2}$** [β]	-30.7	-30.5	-31.0	-31.4	-31.7
**Δ*E*$_{orb,dzx}$** [β]	-	-	-	-	-
**Δ*E*$_{orb,dzy}$** [β]	-	-	-	-	-
**Δ*E*$_{orb,dxy}$** [β]	-	-	-	-	-
**Δ*E*$_{orb,s}$** [β]	-13.6	-13.6	-13.5	-13.8	-14.2
**Δ*E*$_{orb,pz}$** [β]	-9.8	-9.1	-9.9	-10.0	-11.2
**Δ*E*$_{orb,px}$** [β]	-8.3	-8.4	-9.1	-9.1	-10.1
**Δ*E*$_{orb,py}$** [β]	-7.6	-7.4	-7.6	-7.0	-7.7
**Δ*E*$_{orb,pol}$** [β]	-85.2	-85.3	-85.1	-83.4	-77.8
**Δ*E*$_{orb,rest}$** [β]	-21.3	-23.2	-21.5	-21.5	-21.0

Figure S28. Plot of the deformation densities $\Delta\rho_{(i)}$ in fragmentation **5b** with corresponding energy contribution to the total orbital term **Δ*E*** (given in kcal/mol) of the [TM]←ligand σ-donation, the [TM]→ligand π-backdonation and polarization in reference complex *HS* [Fe(***L****^{pyridine}*)$_2$(NCBH$_3$)$_2$]. The direction of the charge flow is yellow → turquoise. The eigenvalues |*v*| indicate the relative size of the charge flow. On the left column are reported $\Delta\rho_{(i)}$ for alpha electrons; on the right are reported $\Delta\rho_{(i)}$ for beta electrons. Figures are reported using until $|v_i|$ = 0.05, with cut-off on $\Delta\rho_{(i)}$=0.003 as this produced the clearest image; please note that the EDA-NOCV analysis are performed by applying the default cutoffs on NOCVs energies (0.5 kcal/mol) and individual SFO contribution (0.001).

ALPHA

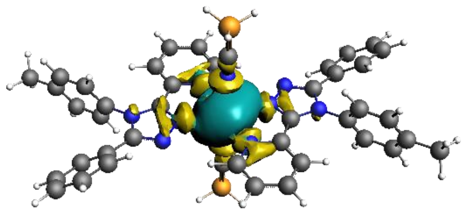

$\Delta\rho_{(5)}$ $\Delta E_5 = -12.4$, $|v_5| = 0.19$ ($s_{[TM]} \leftarrow$ ligand σ)

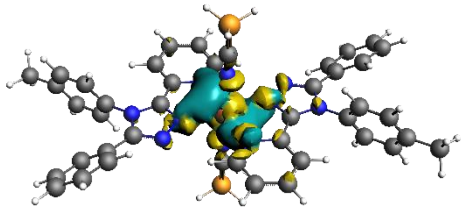

$\Delta\rho_{(6)}$ $\Delta E_6 = -10.5$, $|v_6| = 0.17$ (pol)

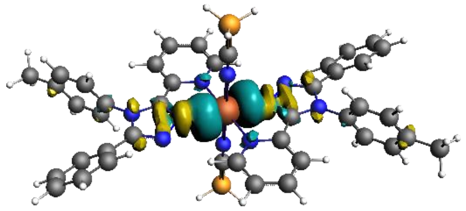

$\Delta\rho_{(8)}$ $\Delta E_8 = -11.5$, $|v_8| = 0.15$ ($p_{[TM]} \leftarrow$ ligand σ)

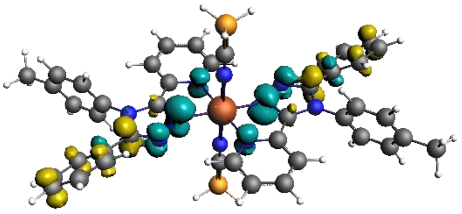

$\Delta\rho_{(10)}$ $\Delta E_{10} = -6.7$, $|v_{10}| = 0.13$ (pol)

BETA

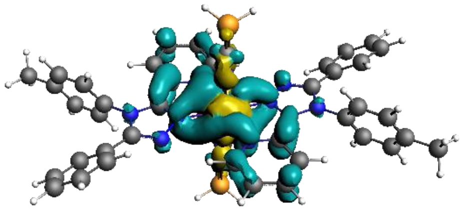

$\Delta\rho_{(1)}$ $\Delta E_1 = -5.0$, $|v_1| = 0.89$ (pol)

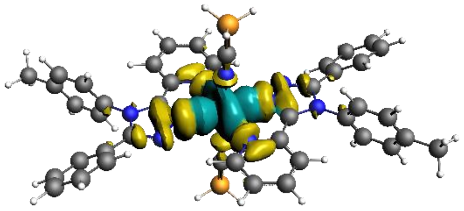

$\Delta\rho_{(2)}$ $\Delta E_2 = -33.4$, $|v_2| = 0.36$ ($d_{[TM]} \leftarrow$ ligand σ)

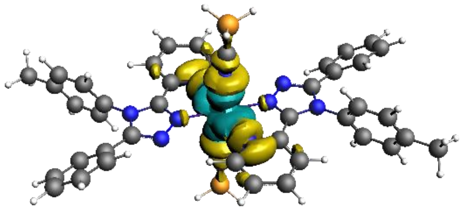

$\Delta\rho_{(3)}$ $\Delta E_3 = -31.0$, $|v_3| = 0.36$ ($d_{[TM]} \leftarrow$ ligand σ)

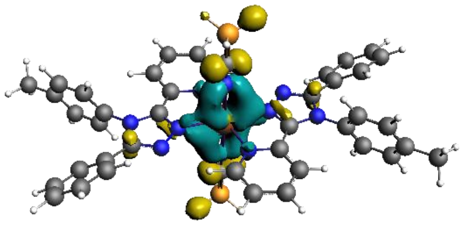

$\Delta\rho_{(4)}$ $\Delta E_4 = -11.9$, $|v_4| = 0.22$ (pol)

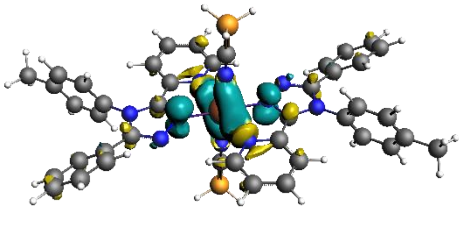

$\Delta\rho_{(11)}$ $\Delta E_{11} = -8.7$, $|v_{11}| = 0.13$ (pol)

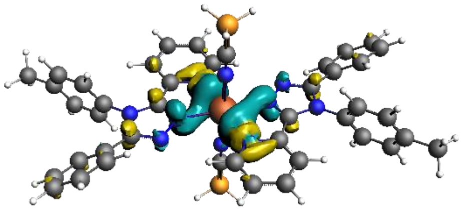

$\Delta\rho_{(12)}$ $\Delta E_{12} = -9.0$, $|v_{12}| = 0.13$ ($p_{[TM]} \leftarrow$ ligand σ)

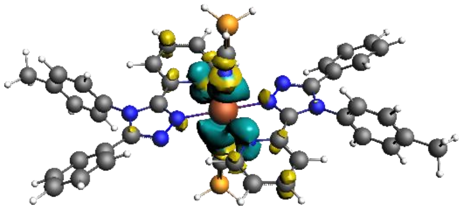

$\Delta\rho_{(15)}$ $\Delta E_{15} = -8.3$, $|v_{15}| = 0.12$ ($p_{[TM]} \leftarrow$ ligand σ)

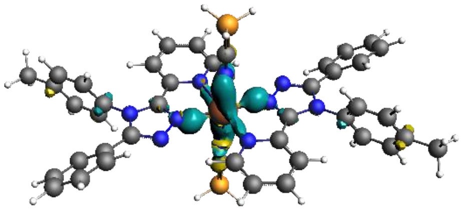

$\Delta\rho_{(19)}$ $\Delta E_{19} = -5.8$, $|v_{19}| = 0.11$ (pol)

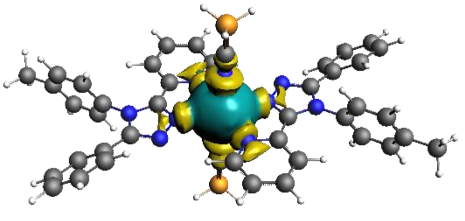

$\Delta\rho_{(7)}$ $\Delta E_{7} = -13.5$, $|v_{7}| = 0.15$ ($s_{[TM]} \leftarrow$ ligand σ)

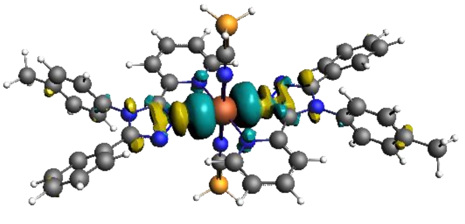

$\Delta\rho_{(9)}$ $\Delta E_{9} = -9.9$, $|v_{9}| = 0.14$ ($s_{[TM]} \leftarrow$ ligand σ)

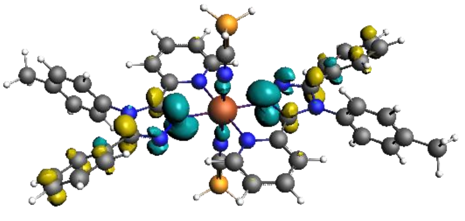

$\Delta\rho_{(13)}$ $\Delta E_{13} = -7.0$, $|v_{13}| = 0.13$ (pol)

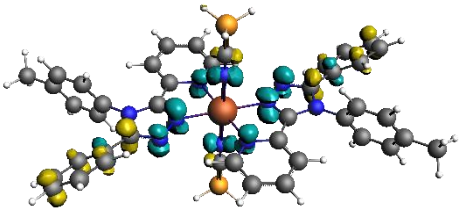

$\Delta\rho_{(14)}$ $\Delta E_{14} = -6.2$, $|v_{14}| = 0.12$ (pol)

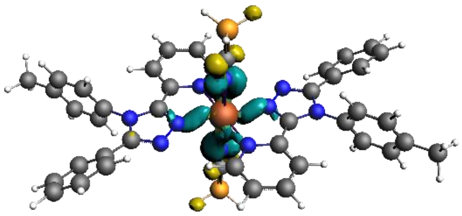

$\Delta\rho_{(20)}$ $\Delta E_{20} = -5.4$, $|v_{20}| = 0.10$ (pol)

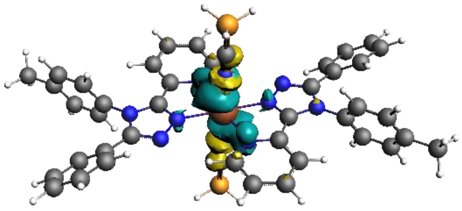

$\Delta\rho_{(21)}$ $\Delta E_{21} = -6.8$, $|v_{21}| = 0.10$ (pol)

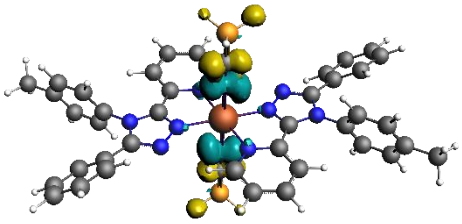

$\Delta\rho_{(22)}$ $\Delta E_{22} = -5.4$, $|v_{22}| = 0.09$ (pol)

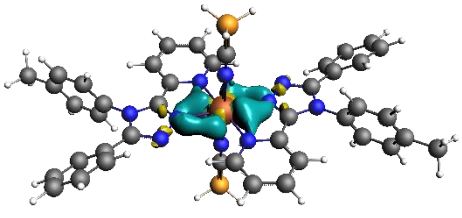

$\Delta\rho_{(24)}$ $\Delta E_{24} = -3.5$, $|v_{24}| = 0.09$ ($d_{[TM]} \rightarrow$ ligand π)

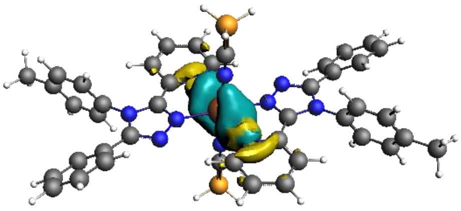

$\Delta\rho_{(16)}$ $\Delta E_{16} = -9.1$, $|v_{16}| = 0.12$ ($p_{[TM]} \leftarrow$ ligand σ)

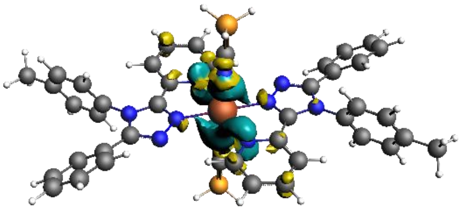

$\Delta\rho_{(17)}$ $\Delta E_{17} = -7.6$, $|v_{17}| = 0.11$ ($p_{[TM]} \leftarrow$ ligand σ)

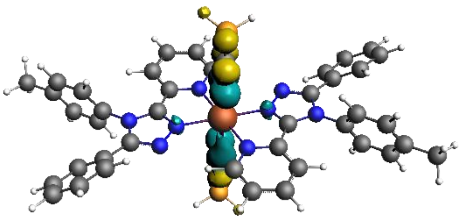

$\Delta\rho_{(18)}$ $\Delta E_{18} = -6.1$, $|v_{18}| = 0.11$ (pol)

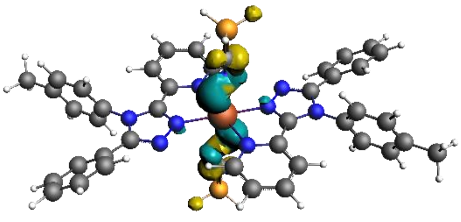

$\Delta\rho_{(23)}$ $\Delta E_{23} = -5.9$, $|v_{23}| = 0.09$ (pol)

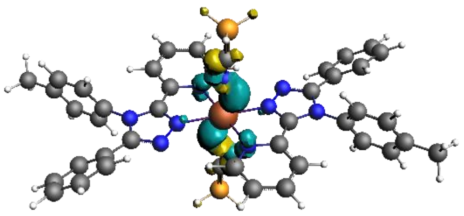

$\Delta\rho_{(25)}$ $\Delta E_{25} = -5.2$, $|v_{25}| = 0.09$ (pol)

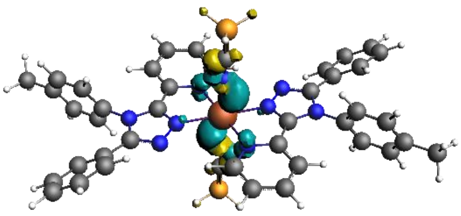

$\Delta\rho_{(26)}$ $\Delta E_{26} = -5.5$, $|v_{26}| = 0.09$ (pol)

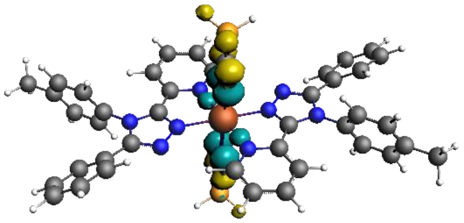

$\Delta\rho_{(27)}$ $\Delta E_{27} = -5.4$, $|v_{27}| = 0.09$ (pol)

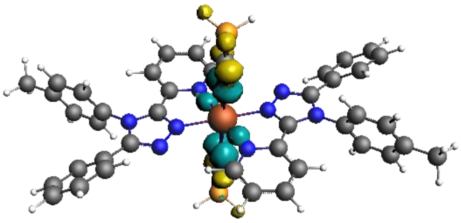

$\Delta\rho_{(28)}$ $\Delta E_{28} = -5.2$, $|v_{28}| = 0.09$ (pol)

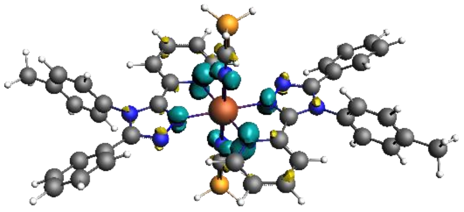

$\Delta\rho_{(30)}$ $\Delta E_{30} = -4.8$, $|v_{30}| = 0.08$ (pol)

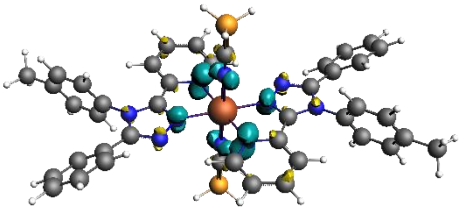

$\Delta\rho_{(29)}$ $\Delta E_{29} = -4.1$, $|v_{29}| = 0.08$ (pol)

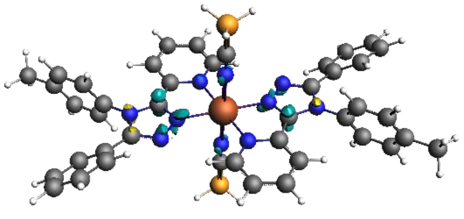

$\Delta\rho_{(31)}$ $\Delta E_{31} = -2.4$, $|v_{31}| = 0.6$ (pol)

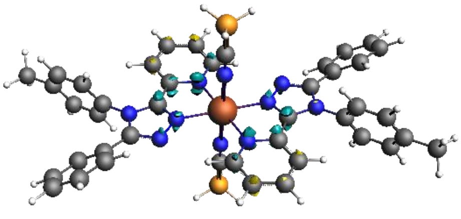

$\Delta\rho_{(32)}$ $\Delta E_{32} = -2.4$, $|v_{32}| = 0.06$ (pol)

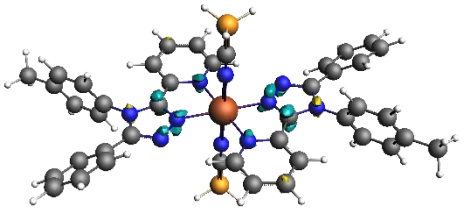

$\Delta\rho_{(35)}$ $\Delta E_{35} = -2.4$, $|v_{35}| = 0.06$ (pol)

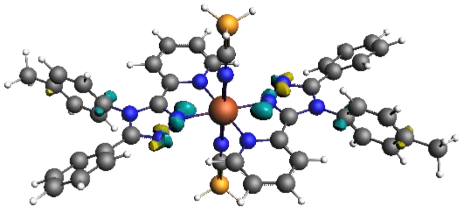

$\Delta\rho_{(37)}$ $\Delta E_{37} = -2.1$, $|v_{37}| = 0.05$ (pol)

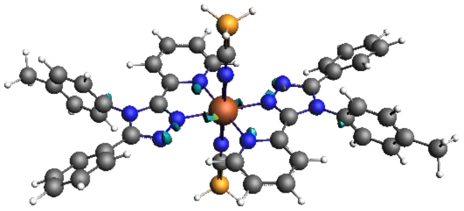

$\Delta\rho_{(38)}$ $\Delta E_{38} = -1.5$, $|v_{38}| = 0.05$ (pol)

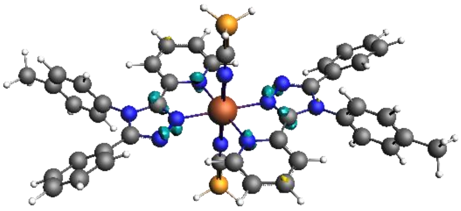

$\Delta\rho_{(33)}$ $\Delta E_{33} = -2.3$, $|v_{33}| = 0.06$ (pol)

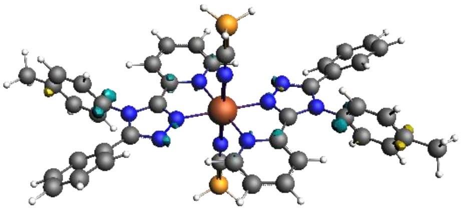

$\Delta\rho_{(34)}$ $\Delta E_{34} = -1.6$, $|v_{34}| = 0.05$ (pol)

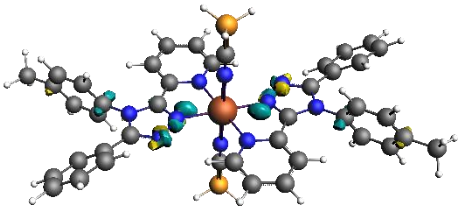

$\Delta\rho_{(36)}$ $\Delta E_{36} = -2.0$, $|v_{36}| = 0.05$ (pol)

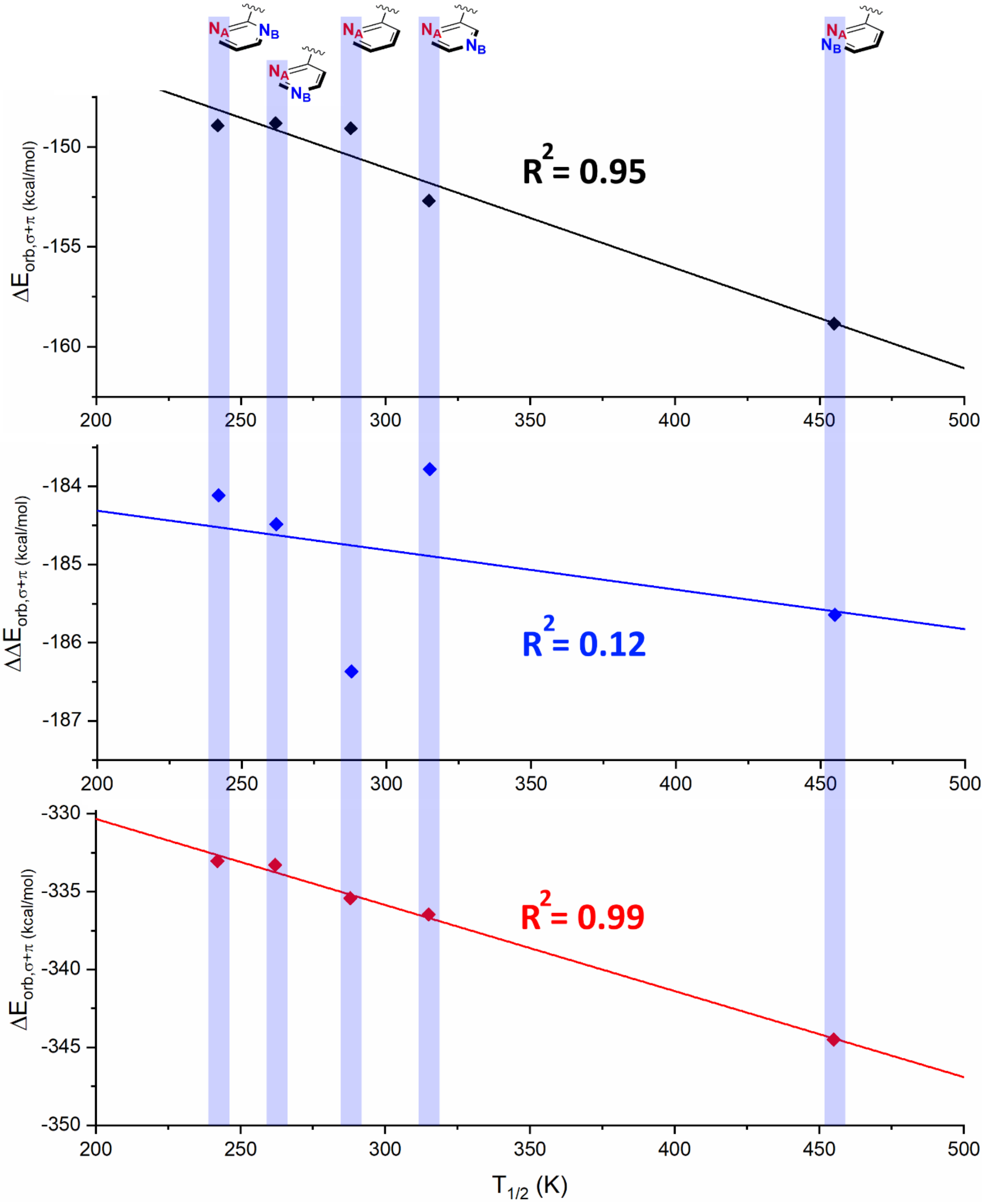

Figure S29. Comparison between correlation between $\Delta E_{orb,\sigma+\pi}$ terms and $T_{1/2}$ for the five [Fe^{II}(L^{azine})$_2$($NCBH_3$)$_2$] complexes using fragmentation **5b**. *LS* state is reported in red (R^2=0.99), *HS* state is reported in black (R^2=0.95) and $\Delta\Delta E_{orb,\sigma+\pi}$ (*HS*-*LS*) term is reported in blue (R^2=0.12).

S3.7 - Fragmentation 5b/5e - *LS* Fe(L^{azine})$_3$$^{2+}$

Table S23. EDA-NOCV results (kcal/mol) reported for fragmentation **5b-5e** for all the five *LS* [Fe(L^{azine})$_3$$^{2+}$] systems. First section (top) reports ΔE_{int} energy splitting **5e**; second section (middle) reports ΔE_{orb} energy splitting; third section (bottom) reports all the nine orbitalic interaction due to **M** - **L$_6$** interaction **5b**.

	$L^{4pyrimidine}$	$L^{2pyrimidine}$	$L^{pyridine}$	$L^{pyrazine}$	$L^{pyridazine}$
ΔE_{int}	-279.67	-279.23	-296.52	-274.75	-306.31
ΔE_{Pauli}	652.76	653.73	659.72	657.48	667.52
ΔE_{elstat}	-402.13 (-43.1%)	-403.34 (-43.2%)	-421.13 (-44.0%)	-397.39 (-42.6%)	-425.32 (-43.7%)
ΔE_{orb}	-519.6 (-55.7%)	-518.99 (-55.6%)	-524.28 (-54.8%)	-524.06 (-56.2%)	-537.97 (-55.2%)
ΔE_{disp}	-10.7 (-1.2%)	-10.63 (-1.2%)	-10.82 (-1.2%)	-10.78 (-1.2%)	-10.54 (-1.1%)
$\Delta E_{orb,\sigma}$	-319.8	-317.5	-315.0	-316.9	-326.8
$\Delta E_{orb,\pi}$	-40.5	-43.5	-52.8	-51.6	-48.5
$\Delta E_{orb,\sigma+\pi}$	-360.3	-361.1	-367.8	-368.5	-375.4
$\Delta E_{orb,pol}$	-128.2	-128.4	-126.9	-125.9	-132.1
$\Delta E_{orb,rest}$	-37.0	-36.4	-36.6	-36.4	-37.7
$\Delta E_{orb,dz2}$	-118.8	-115.6	-112.1	-112.1	-121.6
$\Delta E_{orb,dx2-y2}$	-125.5	-124.9	-123.1	-124.0	-128.3
$\Delta E_{orb,dzx}$	-11.0	-14.9	-13.0	-19.1	-12.1
$\Delta E_{orb,dzy}$	-13.3	-12.1	-21.0	-15.3	-18.4
$\Delta E_{orb,dxy}$	-16.0	-16.5	-18.7	-17.1	-17.9
$\Delta E_{orb,s}$	-25.1	-24.1	-24.2	-24.7	-23.7
$\Delta E_{orb,pz}$	-14.7	-16.02	-18.1	-19.5	-18.0
$\Delta E_{orb,px}$	-18.9	-18.2	-19.3	-19.1	-18.6
$\Delta E_{orb,py}$	-16.6	-18.4	-17.9	-17.1	-16.4

Figure S30. Plot of the deformation densities $\Delta\rho_{(i)}$ in fragmentation **5b** with corresponding energy contribution to the total orbital term $\boldsymbol{\Delta E}$ (given in kcal/mol) of the [TM]←ligand σ-donation, the [TM]→ligand π-backdonation and polarization in reference complex *LS* [Fe($\boldsymbol{L}^{\boldsymbol{pyridine}}$)$_3$$^{2+}$]. The direction of the charge flow is yellow → turquoise. The eigenvalues |*v*| indicate the relative size of the charge flow. Figures are reported using until $|v_i|$ = 0.1, with cut-off on $\Delta\rho_{(i)}$=0.003 as this produced the clearest image; please note that the EDA-NOCV analysis are performed by applying the default cutoffs on NOCVs energies (0.5 kcal/mol) and individual SFO contribution (0.001).

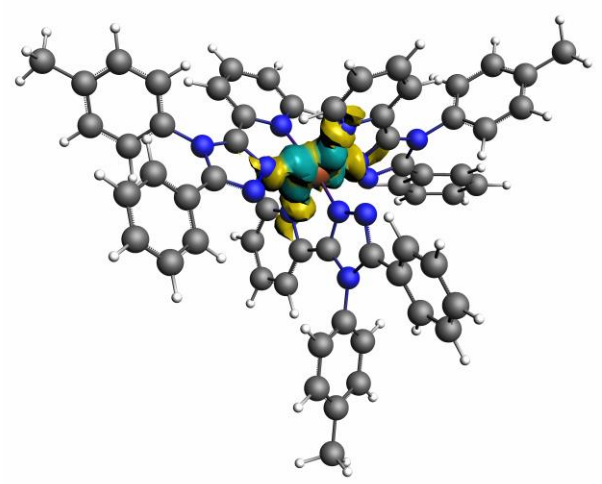

$\boldsymbol{\Delta\rho_{(1)}}$ $\Delta E_1 = -112.2$, $|v_1| = 1.07$ ($d_{[TM]}$←ligand σ)

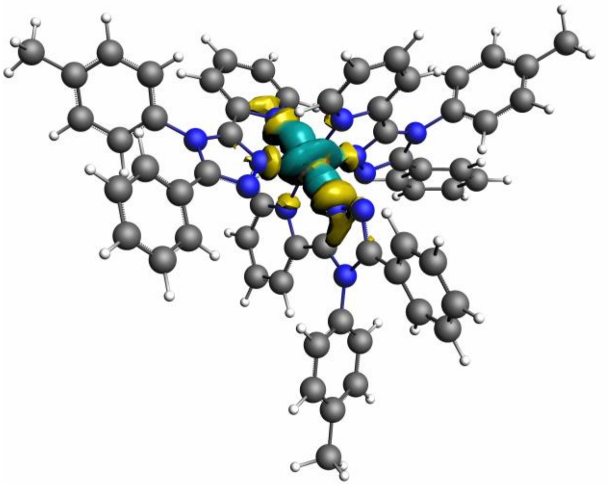

$\boldsymbol{\Delta\rho_{(2)}}$ $\Delta E_2 = -123.2$, $|v_2| = 0.97$ ($d_{[TM]}$←ligand σ)

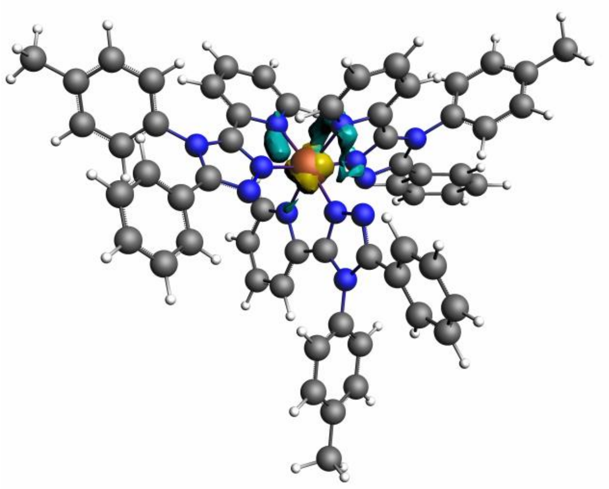

$\boldsymbol{\Delta\rho_{(3)}}$ $\Delta E_3 = -13.0$, $|v_3| = 0.70$ ($d_{[TM]}$→ligand π)

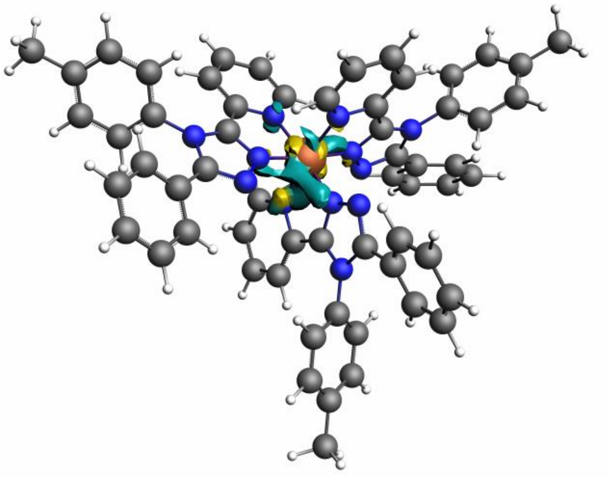

$\boldsymbol{\Delta\rho_{(4)}}$ $\Delta E_4 = -21.1$, $|v_4| = 0.65$ ($d_{[TM]}$→ligand π)

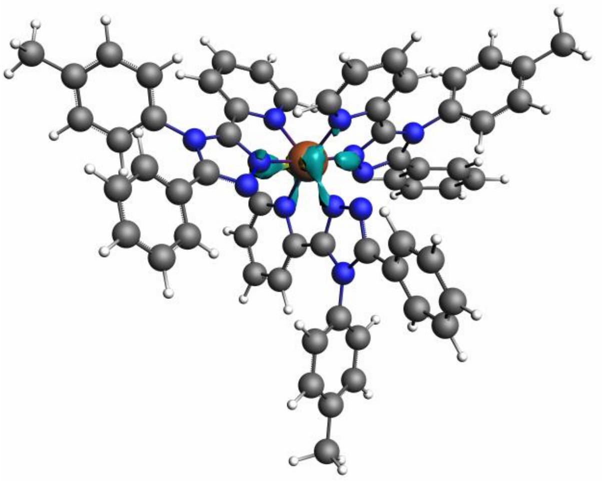

Δρ(5) $\Delta E_5 = -18.7$, $|v_4| = 0.45$ ($d_{[TM]} \rightarrow$ ligand π)

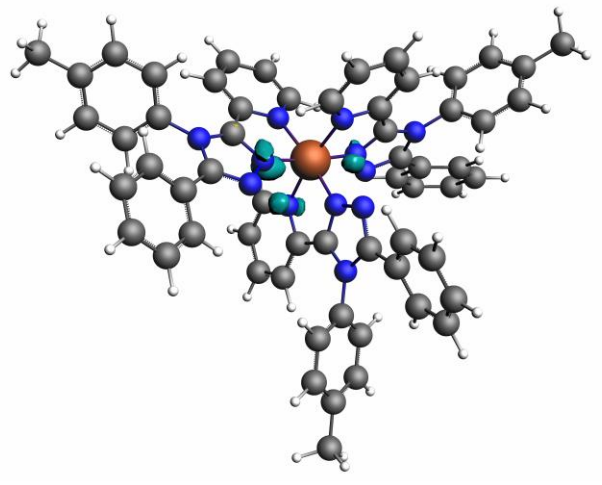

Δρ(6) $\Delta E_6 = -19.5$, $|v_6| = 0.27$ (pol)

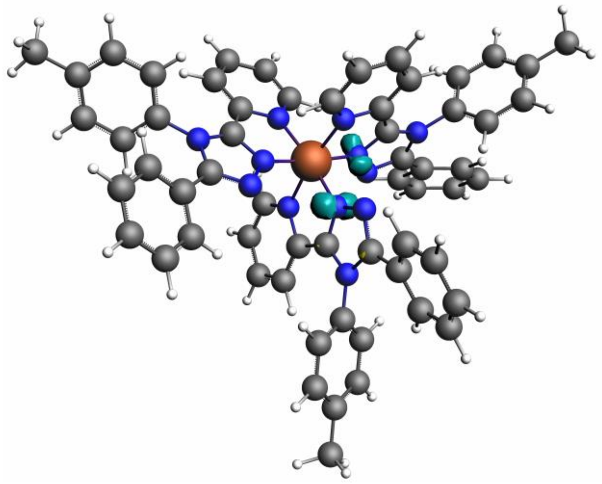

Δρ(7) $\Delta E_7 = -17.5$, $|v_7| = 0.27$ (pol)

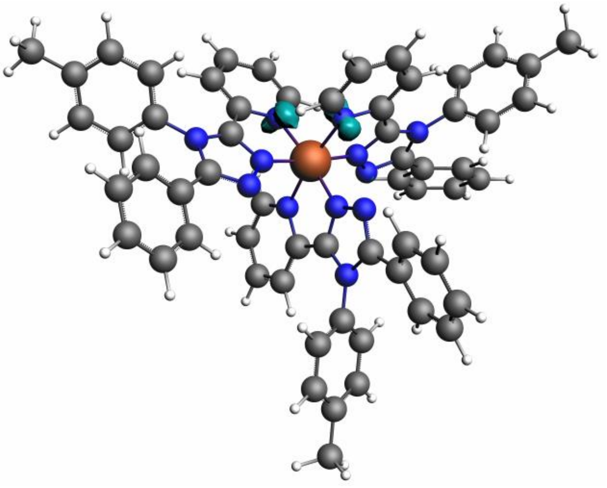

Δρ(8) $\Delta E_8 = -17.8$, $|v_8| = 0.27$ (pol)

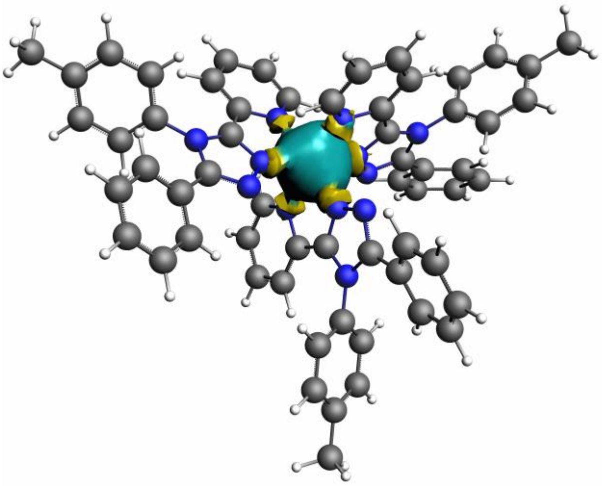

Δρ(9) $\Delta E_9 = -24.3$, $|v_9| = 0.25$ ($s_{[TM]} \leftarrow$ ligand σ)

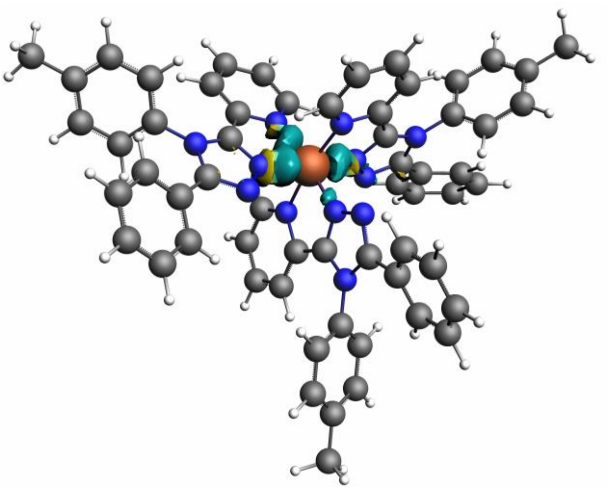

Δρ(10) $\Delta E_{10} = -18.2$, $|v_{10}| = 0.23$ ([$p_{[TM]} \leftarrow$ ligand σ)

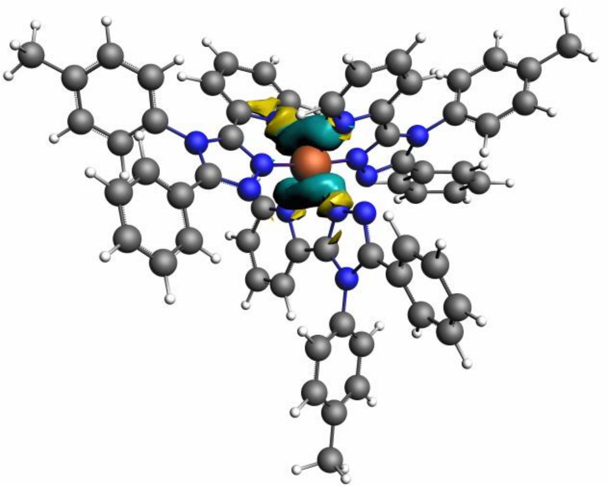

Δρ(11) $\Delta E_{11} = -19.3$, $|v_{11}| = 0.21$ ($p_{[TM]}$←ligand σ)

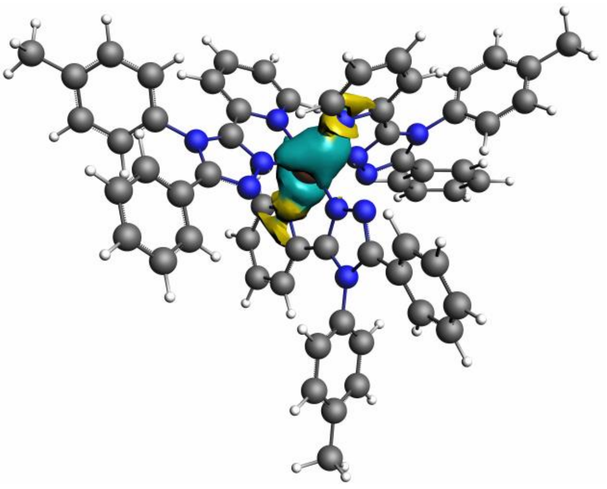

Δρ(12) $\Delta E_{12} = -18.0$, $|v_{12}| = 0.21$ ($p_{[TM]}$←ligand σ)

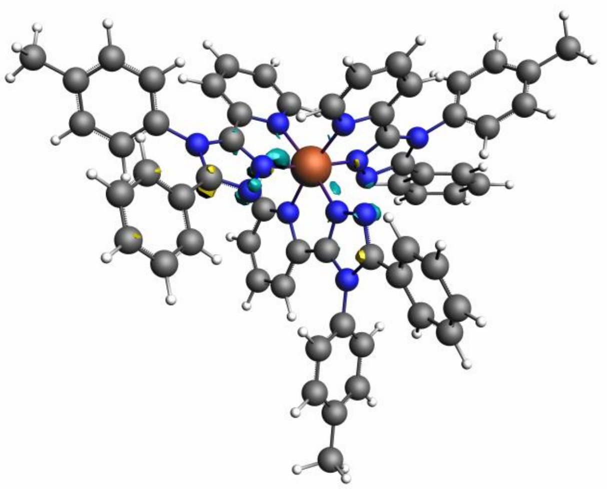

Δρ(13) $\Delta E_{13} = -10.9$, $|v_{13}| = 0.20$ (pol)

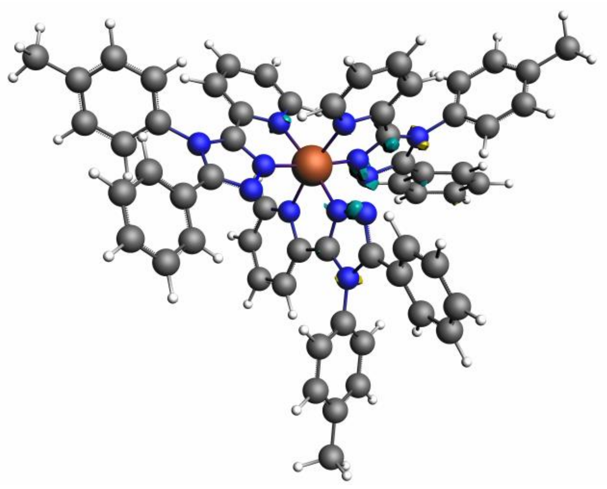

Δρ(14) $\Delta E_{14} = -6.85$, $|v_{14}| = 0.15$ (pol)

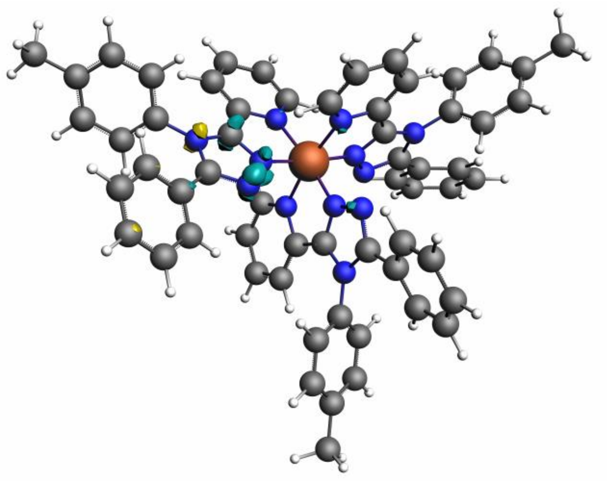

Δρ(15) $\Delta E_{15} = -6.4$, $|v_{15}| = 0.15$ (pol)

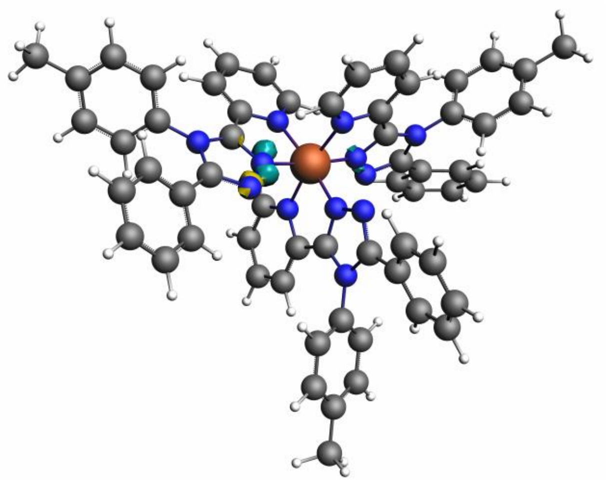

Δρ(16) $\Delta E_{16} = -6.3$, $|v_{16}| = 0.13$ (pol)

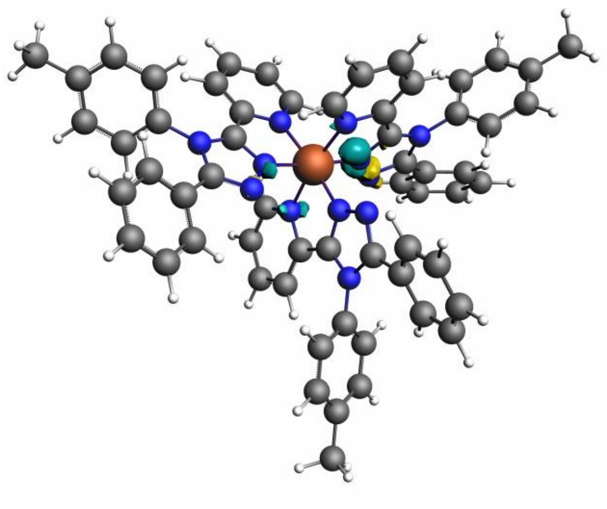

Δρ(17) ΔE_{17} = –6.3, $|v_{17}|$ = 0.12 (pol)

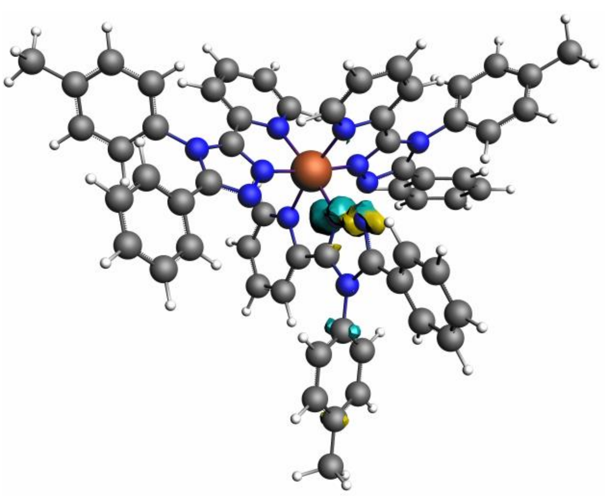

Δρ(18) ΔE_{18}= –6.0, $|v_{18}|$ = 0.12 (pol)

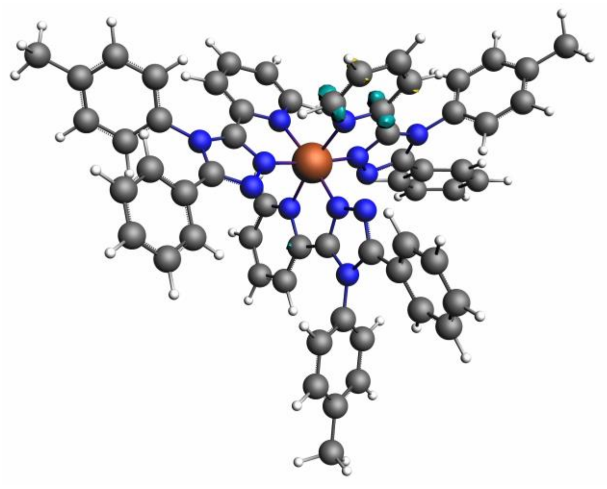

Δρ(19) ΔE_{19} = –3.6, $|v_{19}|$ = 0.10 (pol)

S4 – $Fe(L^{azine})_2(NCBH_3)_2$ COORDINATES

HS $[Fe(L^{pyridine})_2(NCBH_3)_2]$

Fe	4.88195	2.39988	8.03737
N	5.53088	4.13672	6.94254
N	6.10868	3.42167	9.44841
N	6.46417	3.26106	10.75093
N	7.21206	5.22148	10.03272
C	5.25842	4.36899	5.64960
H	4.60645	3.64191	5.16142
C	5.77494	5.46761	4.96506
H	5.52576	5.61860	3.91643
C	6.61341	6.35007	5.64900
H	7.04416	7.21294	5.14299
C	6.90377	6.11656	6.99208
H	7.56339	6.78413	7.53864
C	6.33930	4.99993	7.61884
C	6.55657	4.59536	9.00095
C	7.13945	4.35146	11.10990
C	7.72527	4.55804	12.43343
C	7.81792	5.82693	13.03256
H	7.44408	6.70835	12.51666
C	8.37012	5.95837	14.30669
H	8.43286	6.94541	14.76436
C	8.83164	4.83331	14.99644
H	9.26483	4.94199	15.99072
C	8.73308	3.56765	14.40851
H	9.09112	2.68673	14.94096
C	8.18421	3.42757	13.13539
H	8.11362	2.44699	12.66687
C	7.85747	6.50347	9.99664
C	7.07983	7.66093	10.01977
H	5.99382	7.58508	10.06508
C	7.71414	8.90301	9.98395
H	7.11047	9.81095	9.99880
C	9.11329	9.00310	9.93375
C	9.86759	7.81749	9.91356
H	10.95596	7.87286	9.87003
C	9.25013	6.56850	9.94269
H	9.83576	5.65034	9.92081
C	9.79529	10.34451	9.91415
H	9.07238	11.15879	9.78509
H	10.33733	10.51771	10.85581
H	10.53359	10.40179	9.10246
N	3.22077	3.46507	8.75854
C	2.28613	4.05103	9.14570
B	1.04042	4.83643	9.66598
H	0.14798	4.03708	9.91168
H	0.68991	5.61862	8.79277
H	1.36623	5.44512	10.67618

N	4.23218	0.66331	9.13209
N	3.65641	1.37788	6.62549
N	3.30282	1.53759	5.32230
N	2.55308	-0.42192	6.04100
C	4.50316	0.43170	10.42545
H	5.15568	1.15825	10.91364
C	3.98445	-0.66565	11.11038
H	4.23249	-0.81620	12.15935
C	3.14528	-1.54742	10.42642
H	2.71279	-2.40923	10.93273
C	2.85641	-1.31452	9.08290
H	2.19619	-1.98149	8.53632
C	3.42309	-0.19923	8.45577
C	3.20748	0.20467	7.07319
C	2.62735	0.44731	4.96333
C	2.04323	0.23988	3.63918
C	1.95117	-1.02949	3.04097
H	2.32416	-1.91055	3.55811
C	1.40067	-1.16187	1.76620
H	1.33838	-2.14927	1.30925
C	0.94029	-0.03728	1.07491
H	0.50844	-0.14668	0.08014
C	1.03830	1.22884	1.66194
H	0.68113	2.10940	1.12829
C	1.58548	1.36987	2.93569
H	1.65564	2.35082	3.40350
C	1.90714	-1.70364	6.07706
C	2.68433	-2.86144	6.05620
H	3.77047	-2.78611	6.01305
C	2.04941	-4.10324	6.09120
H	2.65269	-5.01146	6.07828
C	0.65014	-4.20265	6.13849
C	-0.10371	-3.01674	6.15687
H	-1.19217	-3.07162	6.19867
C	0.51434	-1.76805	6.12845
H	-0.07096	-0.84964	6.14877
C	-0.03263	-5.54375	6.15781
H	0.69020	-6.35839	6.28500
H	-0.57711	-5.71641	5.21755
H	-0.76904	-5.60073	6.97132
N	6.54422	1.33423	7.31712
C	7.47955	0.74845	6.93122
B	8.72609	-0.03676	6.41268
H	9.61849	0.76281	6.16767
H	9.07579	-0.81846	7.28664
H	8.40154	-0.64592	5.40238

LS [Fe(L^{pyridine})$_2$(NCBH$_3$)$_2$]

Fe	4.71494	2.35054	7.92512
N	4.15467	3.90609	9.03339
N	3.69177	3.32892	6.58926
N	3.33336	3.13327	5.29396
N	2.55760	5.09969	5.96321
C	4.41048	4.08331	10.34115
H	4.99139	3.29664	10.82114
C	3.97079	5.20414	11.04522
H	4.20886	5.29106	12.10374
C	3.23516	6.18579	10.38250
H	2.87636	7.06938	10.90809
C	2.96124	6.01720	9.02668
H	2.38779	6.75669	8.47465
C	3.43378	4.87300	8.38140
C	3.22356	4.50683	7.00320
C	2.64165	4.20502	4.90925
C	2.05519	4.36747	3.58133
C	1.91406	5.62359	2.96444
H	2.24826	6.52666	3.47006
C	1.36355	5.71498	1.68619
H	1.26291	6.69238	1.21480
C	0.95217	4.56283	1.00948
H	0.52085	4.64032	0.01150
C	1.09841	3.31027	1.61549
H	0.77881	2.40841	1.09374
C	1.64542	3.20980	2.89296
H	1.75168	2.23942	3.37576
C	1.87135	6.36042	5.99439
C	0.47777	6.37886	6.06241
H	-0.07624	5.44177	6.09958
C	-0.18129	7.60637	6.08276
H	-1.27052	7.62524	6.13513
C	0.53218	8.81663	6.04199
C	1.93317	8.76350	5.97997
H	2.50564	9.69097	5.94803
C	2.60914	7.54298	5.95198
H	3.69654	7.50382	5.89429
C	-0.19578	10.13375	6.06248
H	0.50413	10.97766	6.05668
H	-0.85649	10.22906	5.18866
H	-0.83089	10.21747	6.95591
N	3.15770	1.39892	8.44576
C	2.21497	0.78273	8.75522
B	0.95398	-0.04496	9.17022
H	0.01120	0.72335	9.30756
H	1.19633	-0.61740	10.22486
H	0.71605	-0.85892	8.28733
N	5.27519	0.79498	6.81686
N	5.73821	1.37223	9.26095
N	6.09671	1.56796	10.55622

N	6.87294	-0.39824	9.88686
C	5.01923	0.61766	5.50915
H	4.43818	1.40424	5.02919
C	5.45895	-0.50316	4.80507
H	5.22076	-0.59015	3.74659
C	6.19479	-1.48469	5.46774
H	6.55363	-2.36826	4.94213
C	6.46887	-1.31600	6.82351
H	7.04248	-2.05540	7.37550
C	5.99628	-0.17183	7.46880
C	6.20665	0.19444	8.84695
C	6.78876	0.49640	10.94084
C	7.37548	0.33417	12.26867
C	7.51740	-0.92190	12.88547
H	7.18362	-1.82513	12.37987
C	8.06816	-1.01306	14.16363
H	8.16940	-1.99042	14.63496
C	8.47902	0.13928	14.84034
H	8.91054	0.06198	15.83825
C	8.33199	1.39179	14.23442
H	8.65117	2.29380	14.75616
C	7.78473	1.49203	12.95705
H	7.67781	2.46238	12.47433
C	7.55976	-1.65866	9.85552
C	8.95332	-1.67644	9.78688
H	9.50686	-0.73909	9.74936
C	9.61295	-2.90364	9.76633
H	10.70216	-2.92199	9.71347
C	8.90008	-4.11423	9.80752
C	7.49909	-4.06177	9.87018
H	6.92708	-4.98951	9.90247
C	6.82256	-2.84157	9.89837
H	5.73517	-2.80292	9.95660
C	9.62866	-5.43101	9.78683
H	8.92915	-6.27525	9.79279
H	10.28961	-5.52602	10.66050
H	10.26360	-5.51441	8.89326
N	6.27218	3.30216	7.40445
C	7.21491	3.91831	7.09489
B	8.47588	4.74595	6.67973
H	9.41831	3.97746	6.54105
H	8.23297	5.31936	5.62574
H	8.71470	5.55907	7.56316

HS Fe(L^4pyrimidine)₂(NCBH₃)₂

Fe	4.88153	2.40032	8.03543
N	5.52702	4.14809	6.94568
N	6.08468	3.43518	9.45491
N	6.44304	3.26523	10.75044
N	7.19803	5.22855	10.04520
C	5.27575	4.41577	5.65578
N	5.73469	5.46767	4.97349
C	6.52557	6.31811	5.65573
C	6.85266	6.13758	6.99716
H	7.50098	6.84009	7.51206
C	6.31869	5.01631	7.63662
C	6.53544	4.61122	9.01370
C	7.12547	4.35402	11.11560
C	7.71206	4.54768	12.43930
C	7.83787	5.81552	13.03506
H	7.48862	6.70547	12.51679
C	8.39058	5.93399	14.30993
H	8.47903	6.91955	14.76636
C	8.82015	4.79782	15.00226
H	9.25406	4.89667	15.99721
C	8.68834	3.53382	14.41728
H	9.02068	2.64470	14.95255
C	8.13750	3.40569	13.14406
H	8.03960	2.42620	12.67836
C	7.85252	6.50670	10.00099
C	7.08232	7.66870	10.02868
H	5.99653	7.60087	10.08928
C	7.72536	8.90611	9.98141
H	7.12854	9.81836	10.00175
C	9.12435	8.99569	9.91420
C	9.87037	7.80459	9.89148
H	10.95859	7.85212	9.83760
C	9.24504	6.56012	9.93188
H	9.82322	5.63737	9.90918
C	9.81604	10.33135	9.86743
H	9.09454	11.15637	9.89556
H	10.50441	10.44584	10.71704
H	10.41681	10.42945	8.95182
N	3.21124	3.44808	8.73429
C	2.26910	4.02961	9.11054
B	1.01417	4.80810	9.61440
H	0.12569	4.00287	9.85291
H	0.66986	5.58391	8.73351
H	1.32694	5.42226	10.62493
N	4.23666	0.65235	9.12519
N	3.67865	1.36507	6.61590
N	3.32021	1.53478	5.32034
N	2.56608	-0.42883	6.02571
C	4.48809	0.38475	10.41509
N	4.02983	-0.66748	11.09730

C	3.23946	-1.51835	10.41501
C	2.91225	-1.33793	9.07360
H	2.26433	-2.04076	8.55868
C	3.44553	-0.21631	8.43417
C	3.22837	0.18884	7.05713
C	2.63827	0.44563	4.95526
C	2.05193	0.25145	3.63155
C	1.92667	-1.01661	3.03619
H	2.27629	-1.90620	3.55477
C	1.37414	-1.13563	1.76127
H	1.28611	-2.12137	1.30513
C	0.94421	0.00016	1.06853
H	0.51046	-0.09914	0.07355
C	1.07550	1.26439	1.65314
H	0.74291	2.15324	1.11756
C	1.62615	1.39308	2.92639
H	1.72366	2.37275	3.39181
C	1.91254	-1.70742	6.06993
C	2.68360	-2.86885	6.04316
H	3.76935	-2.80025	5.98286
C	2.04147	-4.10672	6.09091
H	2.63899	-5.01853	6.07133
C	0.64253	-4.19731	6.15767
C	-0.10436	-3.00674	6.17934
H	-1.19258	-3.05504	6.23274
C	0.52006	-1.76181	6.13846
H	-0.05874	-0.83946	6.16000
C	-0.04818	-5.53348	6.20503
H	0.67394	-6.35798	6.17758
H	-0.73624	-5.64899	5.35531
H	-0.64912	-5.63152	7.12054
N	6.55208	1.35235	7.33683
C	7.49448	0.77070	6.96141
B	8.74973	-0.00799	6.45869
H	9.63839	0.79713	6.22047
H	9.09342	-0.78347	7.34009
H	8.43764	-0.62252	5.44817
H	2.85302	-2.37642	10.96652
H	6.91255	7.17591	5.10416
H	5.12499	1.10177	10.93816
H	4.63837	3.69912	5.13276

LS Fe($L^{4pyrimidine}$)$_2$($NCBH_3$)$_2$

Fe	4.71608	2.35183	7.92446
N	4.16992	3.91724	9.02145
N	3.70979	3.33122	6.58446
N	3.35845	3.12983	5.29254
N	2.56530	5.09446	5.95025
C	4.42302	4.12754	10.32587
H	5.00493	3.35526	10.82960
N	4.02637	5.19004	11.03288
C	3.31099	6.12426	10.38222
C	2.98967	6.01390	9.03256
H	2.40894	6.77794	8.52357
C	3.44482	4.87801	8.36540
C	3.23273	4.50960	6.99266
C	2.65927	4.19914	4.90104
C	2.08178	4.35310	3.56954
C	1.91545	5.60919	2.95872
H	2.22561	6.51700	3.47098
C	1.37330	5.69327	1.67673
H	1.25316	6.66992	1.20855
C	0.99491	4.53443	0.99199
H	0.56949	4.60641	-0.00890
C	1.16719	3.28240	1.59225
H	0.87468	2.37585	1.06306
C	1.70737	3.18835	2.87283
H	1.83555	2.21810	3.35035
C	1.86604	6.34890	5.98835
C	0.47191	6.35133	6.04632
H	-0.07235	5.40808	6.06502
C	-0.19879	7.57204	6.07931
H	-1.28842	7.57974	6.12418
C	0.50338	8.78960	6.06143
C	1.90523	8.75123	6.00832
H	2.46850	9.68467	5.99331
C	2.59367	7.53802	5.96778
H	3.68164	7.51002	5.91567
C	-0.23727	10.09906	6.09755
H	0.45425	10.94985	6.09669
H	-0.90334	10.19581	5.22805
H	-0.86869	10.16785	6.99489
N	3.14035	1.42856	8.44612
C	2.17906	0.85205	8.77396
B	0.89329	0.07922	9.21356
H	0.16340	0.85544	9.81487
H	1.23007	-0.84368	9.94307
H	0.33461	-0.34729	8.21223
N	5.26207	0.78640	6.82745
N	5.72194	1.37217	9.26459
N	6.07301	1.57328	10.55662
N	6.86454	-0.39213	9.89924
C	5.00948	0.57640	5.52291

H	4.42832	1.34908	5.01893
N	5.40564	-0.48637	4.81602
C	6.11990	-1.42123	5.46699
C	6.44073	-1.31117	6.81678
C	5.98619	-0.17492	7.48377
C	6.19819	0.19342	8.85653
C	6.77114	0.50335	10.94839
C	7.34835	0.34927	12.27999
C	7.51498	-0.90683	12.89071
H	7.20518	-1.81470	12.37833
C	8.05706	-0.99089	14.17274
H	8.17748	-1.96757	14.64078
C	8.43508	0.16795	14.85764
H	8.86043	0.09598	15.85856
C	8.26250	1.42000	14.25750
H	8.55469	2.32660	14.78680
C	7.72244	1.51404	12.97688
H	7.59404	2.48431	12.49946
C	7.56314	-1.64696	9.86101
C	8.95720	-1.65015	9.80156
H	9.50184	-0.70713	9.78172
C	9.62718	-2.87124	9.76842
H	10.71676	-2.87956	9.72239
C	8.92436	-4.08841	9.78754
C	7.52260	-4.04927	9.84216
H	6.95883	-4.98238	9.85814
C	6.83486	-2.83565	9.88288
H	5.74694	-2.80715	9.93617
C	9.66424	-5.39830	9.75117
H	8.97224	-6.24869	9.75281
H	10.33094	-5.49519	10.62017
H	10.29492	-5.46768	8.85335
N	6.29189	3.27494	7.40286
C	7.25349	3.85094	7.07501
B	8.53966	4.62308	6.63536
H	9.27004	3.84587	6.03593
H	8.20356	5.54483	5.90410
H	9.09746	5.05114	7.63653
H	2.98501	6.98827	10.96316
H	6.44541	-2.28550	4.88617
H	7.02087	-2.07541	7.32617

HS Fe(L^2pyrimidine)_2(NCBH_3)_2

Fe	4.88333	2.39934	8.03496
N	5.46959	4.16247	6.94463
N	6.01436	3.49034	9.47592
N	6.40548	3.32354	10.76427
N	7.15358	5.28122	10.02682
C	5.20177	4.44807	5.66029
C	5.67323	5.61796	5.07255
C	6.43606	6.47315	5.87149
N	6.71089	6.20281	7.15676
C	6.21441	5.06218	7.64204
C	6.45968	4.66100	9.02017
C	7.10250	4.41094	11.10321
C	7.72004	4.61342	12.41227
C	7.82819	5.88278	13.00780
H	7.44724	6.76413	12.49672
C	8.40808	6.01281	14.26935
H	8.48334	6.99953	14.72571
C	8.88336	4.88652	14.94815
H	9.33926	4.99455	15.93230
C	8.77026	3.62089	14.36299
H	9.13947	2.73971	14.88724
C	8.19178	3.48158	13.10286
H	8.10904	2.50112	12.63606
C	7.85224	6.53679	9.96838
C	7.13001	7.72717	9.96754
H	6.04129	7.70342	9.98490
C	7.82299	8.93716	9.94802
H	7.26308	9.87304	9.94785
C	9.22604	8.97160	9.93320
C	9.92361	7.75215	9.93359
H	11.01426	7.75523	9.91944
C	9.24618	6.53440	9.95019
H	9.78792	5.58938	9.95053
C	9.97153	10.27962	9.91759
H	9.28208	11.13233	9.91636
H	10.62731	10.36902	10.79570
H	10.61370	10.35525	9.02822
N	3.14301	3.37800	8.66666
C	2.17322	3.93723	9.00427
B	0.87995	4.68662	9.45368
H	0.00309	3.86108	9.66471
H	0.55029	5.44782	8.55446
H	1.13761	5.31553	10.47077
N	4.29650	0.63654	9.12574
N	3.75197	1.30828	6.59427
N	3.36101	1.47479	5.30582
N	2.61142	-0.48199	6.04414
C	4.56398	0.35127	10.41022
C	4.09158	-0.81801	10.99841
C	3.32811	-1.67292	10.19980

N	3.05348	-1.40283	8.91443
C	3.55092	-0.26281	8.42872
C	3.30575	0.13813	7.05052
C	2.66357	0.38756	4.96719
C	2.04613	0.18492	3.65808
C	1.93764	-1.08454	3.06283
H	2.31830	-1.96586	3.57413
C	1.35785	-1.21469	1.80124
H	1.28234	-2.20149	1.34511
C	0.88301	-0.08840	1.12212
H	0.42719	-0.19651	0.13795
C	0.99650	1.17733	1.70698
H	0.62765	2.05851	1.18246
C	1.57490	1.31675	2.96713
H	1.65793	2.29730	3.43369
C	1.91172	-1.73696	6.10323
C	2.63302	-2.92790	6.10498
H	3.72175	-2.90501	6.08783
C	1.93909	-4.13734	6.12513
H	2.49825	-5.07366	6.12601
C	0.53600	-4.17066	6.13971
C	-0.16060	-2.95066	6.13843
H	-1.25126	-2.95287	6.15240
C	0.51779	-1.73345	6.12120
H	-0.02316	-0.78797	6.12015
C	-0.21052	-5.47808	6.15601
H	0.47826	-6.33133	6.15808
H	-0.86607	-5.56759	5.27773
H	-0.85306	-5.55257	7.04520
N	6.62385	1.42073	7.40330
C	7.59350	0.86064	7.06662
B	8.88651	0.11007	6.61843
H	9.76412	0.93484	6.40749
H	9.21503	-0.65089	7.51827
H	8.62901	-0.51920	5.60152
H	2.92011	-2.60395	10.59680
H	6.84329	7.40467	5.47484
H	5.16401	1.08323	10.95461
H	4.60223	3.71590	5.11563
H	4.30765	-1.05063	12.03864
H	5.45693	5.85084	4.03244

LS Fe($L^{2pyrimidine}$)$_2$($NCBH_3$)$_2$

Fe	4.72754	2.35841	7.92262
N	4.20910	3.91741	9.02966
N	3.71838	3.34932	6.59101
N	3.34957	3.15195	5.30166
N	2.56369	5.11488	5.98337
C	4.47962	4.13629	10.32875
H	5.05922	3.36384	10.83537
C	4.03847	5.29194	10.96991
C	3.30659	6.21349	10.22330
N	3.02715	6.01428	8.92441
C	3.48558	4.88711	8.38529
C	3.24873	4.52432	7.00929
C	2.64562	4.22389	4.92789
C	2.05706	4.39328	3.60226
C	1.94118	5.65299	2.98762
H	2.29405	6.54757	3.49617
C	1.39070	5.75595	1.71067
H	1.30908	6.73503	1.23924
C	0.95330	4.61216	1.03542
H	0.52049	4.69898	0.03886
C	1.07483	3.35636	1.63985
H	0.73508	2.46191	1.11818
C	1.62378	3.24378	2.91569
H	1.71164	2.27108	3.39759
C	1.84516	6.35880	6.02294
C	0.45269	6.33806	5.97559
H	-0.07629	5.38665	5.93680
C	-0.24120	7.54776	5.98004
H	-1.33122	7.53733	5.94646
C	0.43863	8.77478	6.03329
C	1.84208	8.75866	6.09185
H	2.38882	9.70095	6.14284
C	2.55097	7.55899	6.08436
H	3.63929	7.54869	6.12412
C	-0.31687	10.07648	6.00415
H	0.23122	10.86863	6.52972
H	-0.46676	10.41283	4.96652
H	-1.30924	9.97219	6.46087
N	3.14580	1.44633	8.44677
C	2.18295	0.86774	8.76641
B	0.89566	0.09074	9.19446
H	0.47464	0.57994	10.23384
H	1.19487	-1.08377	9.36428
H	0.06484	0.19622	8.30231
N	5.24629	0.80121	6.81598
N	5.73265	1.36561	9.25594
N	6.09645	1.55957	10.54722
N	6.87151	-0.40858	9.86830
C	4.98304	0.58668	5.51455
H	4.40762	1.36149	5.00683

C	5.42445	-0.56847	4.87282
C	6.14905	-1.49440	5.62109
N	6.42185	-1.29932	6.92204
C	5.96382	-0.17228	7.46168
C	6.19617	0.18766	8.83925
C	6.79073	0.48230	10.92383
C	7.37207	0.30822	12.25203
C	7.47709	-0.95254	12.86642
H	7.11917	-1.84421	12.35633
C	8.02214	-1.06000	14.14535
H	8.09530	-2.03988	14.61649
C	8.46481	0.08040	14.82290
H	8.89336	-0.00990	15.82100
C	8.35386	1.33735	14.21882
H	8.69764	2.22919	14.74230
C	7.81047	1.45434	12.94097
H	7.73107	2.42789	12.45933
C	7.57767	-1.65948	9.83113
C	8.96973	-1.65403	9.89645
H	9.50798	-0.70863	9.95038
C	9.65019	-2.87114	9.90010
H	10.73936	-2.87287	9.95643
C	8.95783	-4.09074	9.83318
C	7.55527	-4.05942	9.76591
H	6.99831	-4.99596	9.71985
C	6.85940	-2.85187	9.76535
H	5.77134	-2.83021	9.72302
C	9.70277	-5.39859	9.80908
H	9.08232	-6.21885	10.19129
H	10.62227	-5.34507	10.40607
H	9.99553	-5.65758	8.77984
N	6.30954	3.27037	7.40003
C	7.27703	3.84507	7.08739
B	8.57002	4.61692	6.66717
H	9.01172	4.10688	5.64656
H	8.27092	5.78665	6.46658
H	9.38401	4.53309	7.57692
H	2.92938	7.13657	10.66588
H	6.52590	-2.41745	5.17818
H	4.26294	5.45852	12.02107
H	5.20547	-0.73144	3.81993

HS Fe(L^{pyrazine})$_2$(NCBH$_3$)$_2$

Fe	4.87931	2.40098	8.03537
N	5.52752	4.13263	6.94581
N	6.10590	3.41593	9.45133
N	6.45884	3.25195	10.75196
N	7.20690	5.21547	10.03920
C	5.27386	4.39599	5.65788
H	4.62931	3.69331	5.12672
C	5.81875	5.52202	5.03940
H	5.60255	5.73637	3.99232
N	6.62604	6.37484	5.69207
C	6.88122	6.10721	6.97870
H	7.54720	6.79055	7.50313
C	6.33090	4.99326	7.62915
C	6.55298	4.59178	9.00830
C	7.13384	4.34324	11.11460
C	7.71633	4.54869	12.43875
C	7.81761	5.81963	13.03219
H	7.45257	6.70175	12.51130
C	8.36659	5.95116	14.30752
H	8.43621	6.93924	14.76178
C	8.81640	4.82473	15.00282
H	9.24729	4.93371	15.99804
C	8.70932	3.55739	14.41999
H	9.05841	2.67585	14.95721
C	8.16296	3.41658	13.14605
H	8.08565	2.43470	12.68139
C	7.85272	6.49792	9.99473
C	7.07322	7.65442	10.00794
H	5.98740	7.57845	10.05779
C	7.70665	8.89606	9.95577
H	7.10237	9.80362	9.96026
C	9.10581	8.99592	9.89953
C	9.86083	7.81087	9.88906
H	10.94876	7.86672	9.83798
C	9.24476	6.56151	9.93438
H	9.83024	5.64325	9.91895
C	9.78569	10.33796	9.86731
H	9.07950	11.13767	9.61363
H	10.22182	10.57385	10.84986
H	10.60657	10.34955	9.13797
N	3.23043	3.46412	8.75879
C	2.30241	4.05854	9.14951
B	1.06752	4.85496	9.67350
H	0.16822	4.06378	9.91769
H	0.72685	5.64485	8.80401
H	1.40452	5.45429	10.68528
N	4.23201	0.66880	9.12446
N	3.65378	1.38554	6.61883
N	3.30083	1.54950	5.31816
N	2.55491	-0.41509	6.03020

C	4.48539	0.40533	10.41243
H	5.12829	1.10900	10.94429
C	3.94194	-0.72183	11.03016
H	4.15801	-0.93632	12.07724
N	3.13637	-1.57569	10.37674
C	2.88139	-1.30787	9.09011
H	2.21696	-1.99209	8.56486
C	3.43025	-0.19276	8.44041
C	3.20799	0.20901	7.06137
C	2.62727	0.45749	4.95500
C	2.04551	0.25177	3.63058
C	1.94580	-1.01905	3.03661
H	2.31158	-1.90105	3.55719
C	1.39745	-1.15066	1.76102
H	1.32904	-2.13866	1.30640
C	0.94670	-0.02445	1.06597
H	0.51632	-0.13352	0.07054
C	1.05220	1.24278	1.64933
H	0.70237	2.12416	1.11232
C	1.59792	1.38368	2.92354
H	1.67397	2.36546	3.38860
C	1.91172	-1.69887	6.07442
C	2.69396	-2.85374	6.05705
H	3.77938	-2.77506	6.00280
C	2.06349	-4.09684	6.10325
H	2.66965	-5.00305	6.08834
C	0.66441	-4.20008	6.16105
C	-0.09343	-3.01705	6.16960
H	-1.18162	-3.07591	6.21040
C	0.51984	-1.76584	6.12997
H	-0.06816	-0.84913	6.14050
C	-0.00870	-5.54397	6.23254
H	0.65881	-6.34317	5.88787
H	-0.92430	-5.56360	5.62708
H	-0.29878	-5.77540	7.26873
N	6.52937	1.33761	7.31302
C	7.45850	0.74391	6.92370
B	8.69481	-0.05153	6.40166
H	9.59363	0.74045	6.15840
H	9.03496	-0.84076	7.27194
H	8.35965	-0.65148	5.38968

LS Fe(L^{pyrazine})$_2$(NCBH$_3$)$_2$

Fe	4.71630	2.35124	7.92469
N	4.17181	3.89758	9.01910
N	3.70049	3.32428	6.58123
N	3.34501	3.12480	5.28838
N	2.56459	5.09233	5.95253
C	4.42329	4.10341	10.32054
H	5.00238	3.33734	10.83602
C	3.96299	5.25279	10.96669
H	4.17900	5.40093	12.02516
N	3.25097	6.20404	10.34276
C	2.99883	6.00127	9.04324
H	2.41932	6.76148	8.52110
C	3.45050	4.86348	8.36721
C	3.23036	4.50293	6.99206
C	2.65093	4.19629	4.90052
C	2.06640	4.35710	3.57258
C	1.91916	5.61480	2.96034
H	2.24993	6.51742	3.46900
C	1.36834	5.70683	1.68247
H	1.26261	6.68481	1.21356
C	0.96270	4.55422	1.00294
H	0.53046	4.63239	0.00544
C	1.11563	3.30037	1.60464
H	0.80070	2.39863	1.08000
C	1.66361	3.19869	2.88146
H	1.77472	2.22728	3.36105
C	1.87379	6.35140	5.99272
C	0.47979	6.36380	6.05246
H	-0.07131	5.42452	6.07090
C	-0.18199	7.58932	6.08809
H	-1.27145	7.60492	6.13503
C	0.52896	8.80175	6.07111
C	1.93036	8.75340	6.01599
H	2.50042	9.68271	6.00260
C	2.60990	7.53532	5.97275
H	3.69764	7.49974	5.91969
C	-0.20215	10.11648	6.11038
H	0.49579	10.96192	6.12094
H	-0.86018	10.22418	5.23598
H	-0.84030	10.18367	7.00299
N	3.15129	1.41044	8.44945
C	2.20403	0.80321	8.76185
B	0.93775	-0.01175	9.18034
H	0.00394	0.76707	9.31347
H	1.17781	-0.58030	10.23675
H	0.69614	-0.82556	8.29934
N	5.26103	0.80503	6.83019
N	5.73194	1.37808	9.26821
N	6.08690	1.57722	10.56125
N	6.86698	-0.39046	9.89715

C	5.01025	0.59959	5.52854
H	4.43126	1.36573	5.01303
C	5.47108	-0.54950	4.88226
H	5.25559	-0.69737	3.82364
N	6.18297	-1.50082	5.50623
C	6.43441	-1.29845	6.80594
H	7.01402	-2.05860	7.32807
C	5.98221	-0.16095	7.48213
C	6.20191	0.19936	8.85740
C	6.78026	0.50533	10.94933
C	7.36391	0.34408	12.27762
C	7.51026	-0.91374	12.88982
H	7.17939	-1.81617	12.38090
C	8.06028	-1.00613	14.16802
H	8.16533	-1.98421	14.63688
C	8.46597	0.14624	14.84792
H	8.89758	0.06778	15.84567
C	8.31388	1.40022	14.24628
H	8.62883	2.30178	14.77122
C	7.76670	1.50226	12.96915
H	7.65622	2.47377	12.48962
C	7.55694	-1.64999	9.85722
C	8.95098	-1.66340	9.79847
H	9.50277	-0.72452	9.78050
C	9.61189	-2.88939	9.76320
H	10.70137	-2.90579	9.71704
C	8.90004	-4.10131	9.77957
C	7.49864	-4.05194	9.83368
H	6.92789	-4.98083	9.84658
C	6.81996	-2.83337	9.87655
H	5.73221	-2.79700	9.92889
C	9.63022	-5.41656	9.74075
H	8.93167	-6.26149	9.72940
H	10.28735	-5.52490	10.61574
H	10.26915	-5.48408	8.84873
N	6.28123	3.29211	7.39983
C	7.22816	3.89970	7.08715
B	8.49402	4.71512	6.66831
H	9.42770	3.93647	6.53323
H	8.25285	5.28496	5.61284
H	8.73659	5.52786	7.55004

HS Fe(L^pyridazine^)$_2$(NCBH$_3$)$_2$

Fe	4.88352	2.39902	8.03517
N	5.54164	4.15302	6.97955
N	6.11879	3.42547	9.47096
N	6.47465	3.26581	10.77099
N	7.22426	5.22841	10.05598
N	5.24213	4.33611	5.70107
C	5.76173	5.39102	5.06346
H	5.47240	5.48496	4.01704
C	6.62111	6.31086	5.67536
C	6.93484	6.11595	7.00975
H	7.60616	6.78655	7.53860
C	6.36034	5.01017	7.65059
C	6.56890	4.60052	9.02751
C	7.14988	4.35715	11.13110
C	7.73222	4.56278	12.45594
C	7.84098	5.83368	13.04813
H	7.48113	6.71746	12.52638
C	8.39033	5.96418	14.32359
H	8.46544	6.95266	14.77626
C	8.83303	4.83635	15.02111
H	9.26402	4.94424	16.01645
C	8.71777	3.56887	14.44005
H	9.06026	2.68576	14.97906
C	8.17117	3.42936	13.16600
H	8.08514	2.44729	12.70332
C	7.86671	6.51158	10.01184
C	7.08586	7.66698	10.02213
H	6.00008	7.58895	10.06949
C	7.71746	8.91036	9.97506
H	7.11170	9.81696	9.98179
C	9.11624	9.01320	9.92616
C	9.87374	7.82914	9.92131
H	10.96215	7.88682	9.88230
C	9.25963	6.57902	9.96122
H	9.84690	5.66179	9.95289
C	9.79576	10.35528	9.88102
H	9.06589	11.17337	9.89121
H	10.46853	10.48276	10.74124
H	10.41092	10.45303	8.97497
N	3.22307	3.44228	8.76257
C	2.28752	4.02288	9.15545
B	1.04088	4.80082	9.68226
H	0.15057	3.99698	9.92007
H	0.69039	5.59118	8.81678
H	1.36658	5.39912	10.69850
N	4.22507	0.64518	9.09089
N	3.64809	1.37274	6.59936
N	3.29213	1.53239	5.29937
N	2.54127	-0.42955	6.01486
N	4.52459	0.46207	10.36935

C	4.00429	-0.59236	11.00719
H	4.29375	-0.68640	12.05358
C	3.14404	-1.51158	10.39558
C	2.83037	-1.31669	9.06119
H	2.15832	-1.98676	8.53257
C	3.40571	-0.21149	8.42007
C	3.19727	0.19807	7.04312
C	2.61612	0.44143	4.93954
C	2.03323	0.23614	3.61487
C	1.92331	-1.03475	3.02282
H	2.28261	-1.91874	3.54457
C	1.37349	-1.16492	1.74754
H	1.29747	-2.15338	1.29497
C	0.93145	-0.03681	1.05007
H	0.50008	-0.14445	0.05488
C	1.04786	1.23063	1.63100
H	0.70588	2.11396	1.09203
C	1.59495	1.36984	2.90486
H	1.68190	2.35187	3.36744
C	1.89761	-1.71213	6.05949
C	2.67736	-2.86828	6.04920
H	3.76321	-2.79130	6.00154
C	2.04457	-4.11104	6.09668
H	2.64944	-5.01822	6.08993
C	0.64570	-4.21251	6.14608
C	-0.11067	-3.02774	6.15091
H	-1.19912	-3.08438	6.19030
C	0.50461	-1.77822	6.11055
H	-0.08172	-0.86036	6.11902
C	-0.03509	-5.55391	6.19182
H	0.69399	-6.37270	6.18155
H	-0.70832	-5.68096	5.33190
H	-0.64998	-5.65083	7.09814
N	6.54402	1.35566	7.30790
C	7.47975	0.77493	6.91565
B	8.72663	-0.00322	6.38972
H	9.61708	0.80050	6.15208
H	9.07666	-0.79330	7.25565
H	8.40142	-0.60187	5.37352
H	2.73220	-2.34735	10.95808
H	7.03230	7.14709	5.11306

LS Fe(L^{pyridazine})$_2$(NCBH$_3$)$_2$

Fe	4.71447	2.35114	7.92541
N	4.07228	3.83378	9.02280
N	5.35949	0.87044	6.82656
N	3.67019	3.28676	6.57187
N	3.27163	3.06519	5.29723
N	2.57217	5.07799	5.93516
N	4.25002	3.89096	10.32915
C	3.82799	4.97298	10.99525
H	4.00856	4.94447	12.06956
C	3.20281	6.06257	10.38241
H	2.88914	6.92712	10.96384
C	2.98621	5.99165	9.01519
H	2.48767	6.78940	8.47084
C	3.42821	4.84422	8.35232
C	3.24366	4.48790	6.97081
C	2.60420	4.15225	4.90453
C	1.99531	4.30494	3.58570
C	1.86471	5.55426	2.95289
H	2.22752	6.45802	3.43729
C	1.28887	5.63730	1.68523
H	1.19663	6.60928	1.20102
C	0.84140	4.48319	1.03519
H	0.38978	4.55399	0.04570
C	0.97871	3.23703	1.65653
H	0.63287	2.33358	1.15457
C	1.55164	3.14476	2.92319
H	1.65602	2.17980	3.41701
C	1.92457	6.35837	5.96454
C	0.53180	6.41976	6.03024
H	-0.05052	5.49983	6.06058
C	-0.08842	7.66705	6.05576
H	-1.17662	7.71966	6.10667
C	0.66251	8.85485	6.02316
C	2.06121	8.75848	5.96331
H	2.66226	9.66789	5.93746
C	2.69904	7.51755	5.92931
H	3.78472	7.44473	5.87126
C	-0.02387	10.19400	6.04821
H	0.70243	11.01505	6.07005
H	-0.66108	10.32306	5.16124
H	-0.67623	10.28511	6.92827
N	3.20785	1.29793	8.40762
C	2.31067	0.61418	8.70903
B	1.11387	-0.30842	9.11061
H	0.12368	0.38984	9.28064
H	1.40821	-0.89375	10.14433
H	0.92326	-1.10853	8.20428
N	5.75797	1.41403	9.27887
N	6.15294	1.63301	10.55515
N	6.85854	-0.37645	9.91337

N	5.18569	0.81686	5.51958
C	5.61284	-0.26171	4.85127
H	5.43555	-0.23016	3.77656
C	6.23980	-1.35126	5.46249
H	6.55789	-2.21298	4.87927
C	6.45304	-1.28356	6.83043
H	6.95318	-2.08109	7.37361
C	6.00598	-0.13933	7.49548
C	6.18758	0.21464	8.87796
C	6.82191	0.54647	10.94646
C	7.42800	0.39143	12.26634
C	7.55767	-0.85920	12.89677
H	7.19638	-1.76204	12.40952
C	8.13052	-0.94462	14.16563
H	8.22204	-1.91753	14.64809
C	8.57590	0.20839	14.81910
H	9.02522	0.13573	15.80951
C	8.43942	1.45579	14.20012
H	8.78360	2.35834	14.70482
C	7.86938	1.55045	12.93229
H	7.76547	2.51642	12.44036
C	7.50768	-1.65597	9.88314
C	8.90077	-1.71568	9.82296
H	9.48220	-0.79504	9.79738
C	9.52248	-2.96221	9.79680
H	10.61094	-3.01349	9.75024
C	8.77274	-4.15090	9.82322
C	7.37369	-4.05621	9.87755
H	6.77355	-4.96634	9.89864
C	6.73436	-2.81608	9.91217
H	5.64840	-2.74463	9.96635
C	9.46068	-5.48925	9.79757
H	8.73539	-6.31104	9.77049
H	10.09423	-5.61996	10.68693
H	10.11688	-5.57737	8.92007
N	6.22115	3.40437	7.44301
C	7.11814	4.08928	7.14367
B	8.31831	5.00958	6.74688
H	9.30743	4.30874	6.58135
H	8.03013	5.59565	5.71185
H	8.50682	5.80926	7.65401

S5 – Fe(L^{azine})$_2$(NCBH$_3$)$_2$ COORDINATES

LS Fe($L^{4pyrimindine}$)$_3$(BF_4)$_2$

Fe	0.00000	0.00000	0.00000
C	2.88174	-0.85002	0.03158
H	2.47942	-1.86176	0.06206
C	4.68656	0.54344	-0.02163
H	5.77191	0.64720	-0.06203
C	3.85885	1.66329	0.01553
H	4.25977	2.67413	0.01006
C	2.48228	1.43706	0.05565
C	1.41397	2.39579	0.07932
C	-0.02090	4.06086	0.10367
C	-0.62420	5.38658	0.11932
C	-0.00827	6.49534	0.72945
H	0.95617	6.39174	1.22098
C	-0.64718	7.73327	0.72486
H	-0.16917	8.58661	1.20416
C	-1.89568	7.88139	0.11429
H	-2.38619	8.85421	0.10776
C	-2.51512	6.78101	-0.48739
H	-3.48635	6.89549	-0.96731
C	-1.88873	5.53856	-0.48414
H	-2.35898	4.67732	-0.95630
C	2.45557	4.66647	0.15916
C	2.78486	5.38061	-0.99263
H	2.19314	5.25963	-1.89935
C	3.87217	6.25207	-0.95858
H	4.13290	6.81265	-1.85656
C	4.63884	6.41934	0.20701
C	4.28479	5.67846	1.34737
H	4.86681	5.79082	2.26241
C	3.19653	4.80699	1.33382
H	2.91861	4.24666	2.22660
C	5.78984	7.38521	0.24023
H	5.43650	8.39336	0.50522
H	6.28043	7.46037	-0.73784
H	6.53904	7.09522	0.98655
C	-0.01177	-1.45870	-2.45256
C	-0.16869	0.82293	-2.85450
H	-0.22740	1.83115	-2.44739
C	-0.20488	-0.57828	-4.65261
H	-0.26787	-0.68792	-5.73648
C	-0.09532	-1.69390	-3.82532
H	-0.07984	-2.70589	-4.22295
C	0.05821	-2.40574	-1.37514
C	0.13481	-4.05436	0.07891
C	0.18579	-5.37162	0.69779
C	0.74199	-6.49498	0.05679
H	1.16118	-6.41285	-0.94288

C	0.78173	-7.72147	0.71590
H	1.22140	-8.58468	0.21796
C	0.27050	-7.84479	2.01068
H	0.30222	-8.80781	2.51912
C	-0.27583	-6.73029	2.65544
H	-0.67162	-6.82353	3.66597
C	-0.31719	-5.49972	2.00834
H	-0.73934	-4.62607	2.50195
C	0.11153	-4.68240	-2.39556
C	-1.05569	-5.38792	-2.68686
H	-1.94290	-5.25383	-2.06910
C	-1.06103	-6.26769	-3.76774
H	-1.97075	-6.82271	-3.99800
C	0.08078	-6.45172	-4.56574
C	1.23715	-5.71846	-4.25049
H	2.13433	-5.84440	-4.85700
C	1.26274	-4.83852	-3.16908
H	2.16850	-4.28522	-2.92065
C	0.07275	-7.42786	-5.70862
H	0.32659	-8.43764	-5.35185
H	0.80833	-7.15794	-6.47566
H	-0.91660	-7.48939	-6.17824
C	-1.54212	0.19565	2.38610
C	0.67947	-0.24273	2.89962
H	1.69109	-0.43725	2.54641
C	-0.78505	-0.00986	4.63093
H	-0.94591	-0.00798	5.71028
C	-1.84041	0.21220	3.74894
H	-2.85516	0.38692	4.09836
C	-2.42783	0.34006	1.26599
C	-3.98797	0.57458	-0.26241
C	-5.25714	0.79379	-0.94333
C	-6.29036	1.57762	-0.39580
H	-6.17030	2.05445	0.57385
C	-7.47167	1.76714	-1.10965
H	-8.26474	2.37960	-0.68248
C	-7.63872	1.18261	-2.36794
H	-8.56678	1.33123	-2.91892
C	-6.61173	0.41089	-2.92101
H	-6.73836	-0.04344	-3.90294
C	-5.42677	0.21775	-2.21813
H	-4.62143	-0.38114	-2.64004
C	-4.69123	0.85149	2.17594
C	-5.61983	-0.13295	2.51290
H	-5.64290	-1.07289	1.96271
C	-6.52230	0.11539	3.54561
H	-7.25676	-0.64716	3.80585
C	-6.50684	1.32911	4.25329
C	-5.55586	2.29797	3.89074
H	-5.53343	3.25123	4.41947
C	-4.64968	2.06989	2.85579
H	-3.92650	2.83134	2.56368

C	-7.47100	1.57593	5.37994
H	-8.43087	1.07562	5.20287
H	-7.65685	2.64687	5.52435
H	-7.06855	1.18202	6.32566
N	1.97785	0.15314	0.05446
N	4.20226	-0.71209	-0.00715
N	0.16924	1.90876	0.01469
N	1.33506	3.76266	0.13504
N	-0.72774	2.92199	0.03479
N	-0.03341	-0.17453	-1.95921
N	-0.24785	0.67754	-4.17262
N	0.03175	-1.90465	-0.13452
N	0.12568	-3.77063	-1.28162
N	0.07899	-2.90584	0.77331
N	-0.25398	-0.02490	1.95409
N	0.47186	-0.24159	4.21220
N	-1.89649	0.15314	0.05446
N	-2.84046	0.28897	-0.89785
N	-3.75897	0.61272	1.10692

LS Fe(L^{2pyrimindine})$_3$(BF$_4$)$_2$

Fe	0.00000	0.00000	0.00000
N	-0.24241	-0.11645	-1.95096
N	-1.90357	0.11131	-0.06597
N	-2.85263	0.25940	0.87997
N	-3.80398	0.26466	-1.13959
N	-0.06883	-0.07791	1.96289
N	-0.02418	-1.90323	0.22044
N	0.01273	-2.94812	-0.63965
N	-0.19527	-3.71088	1.44904
N	1.96707	0.11131	-0.06597
N	0.19064	1.90541	-0.08708
N	-0.68992	2.92951	-0.16223
N	1.39173	3.73476	-0.25591
C	0.69719	-0.27623	-2.89922
H	1.72676	-0.35879	-2.55783
C	0.36642	-0.33268	-4.24942
H	1.13793	-0.46360	-5.00505
C	-0.98270	-0.21125	-4.58982
H	-1.31441	-0.23414	-5.62906
N	-1.93877	-0.06332	-3.66183
C	-1.54621	-0.02796	-2.39225
C	-2.45272	0.10498	-1.28146
C	-4.02227	0.35658	0.22842
C	-5.30802	0.53415	0.89134
C	-5.32805	1.21758	2.12219
H	-4.39604	1.61085	2.52504
C	-6.52946	1.39600	2.80207
H	-6.54108	1.93464	3.74890
C	-7.71922	0.89090	2.26829
H	-8.65914	1.03313	2.80063
C	-7.70264	0.20077	1.05278
H	-8.62663	-0.20421	0.64201
C	-6.50664	0.02140	0.36158
H	-6.50528	-0.52739	-0.57771
C	-4.77148	0.41503	-2.19516
C	-5.07293	-0.66849	-3.01795
H	-4.56336	-1.62090	-2.87813
C	-6.03301	-0.51184	-4.01518
H	-6.27165	-1.35588	-4.66279
C	-6.70072	0.71002	-4.19944
C	-6.36765	1.78274	-3.35665
H	-6.86800	2.74261	-3.48720
C	-5.40757	1.64495	-2.35650
H	-5.15908	2.47782	-1.69988
C	-7.76165	0.85837	-5.25459
H	-7.58207	0.18745	-6.10342
H	-7.81554	1.88789	-5.62912
H	-8.75172	0.60823	-4.84387
C	-0.13776	0.95392	2.82288
H	-0.10117	1.95120	2.38990

C	-0.26695	0.74624	4.19222
H	-0.32165	1.58872	4.87823
C	-0.32736	-0.57382	4.64532
H	-0.41689	-0.81044	5.70668
N	-0.28857	-1.61647	3.80330
C	-0.17159	-1.33812	2.50903
C	-0.14765	-2.34370	1.47556
C	-0.09558	-4.05898	0.10758
C	-0.10370	-5.40871	-0.44296
C	-0.79563	-6.46973	0.17059
H	-1.34836	-6.30859	1.09327
C	-0.79079	-7.73256	-0.41720
H	-1.33395	-8.54820	0.05831
C	-0.09917	-7.95356	-1.61156
H	-0.09388	-8.94544	-2.06225
C	0.58223	-6.90048	-2.22995
H	1.12004	-7.07037	-3.16193
C	0.57860	-5.63340	-1.65439
H	1.10460	-4.80659	-2.12925
C	-0.25702	-4.59115	2.58775
C	-1.41606	-4.63673	3.36014
H	-2.26216	-3.99544	3.11656
C	-1.47165	-5.51331	4.44142
H	-2.37539	-5.55203	5.05008
C	-0.38825	-6.34864	4.75989
C	0.76660	-6.27131	3.96447
H	1.62302	-6.90397	4.19869
C	0.84082	-5.39832	2.88125
H	1.73704	-5.35041	2.26377
C	-0.47073	-7.31719	5.90684
H	-1.15413	-6.96068	6.68701
H	0.51378	-7.49084	6.35811
H	-0.84779	-8.29203	5.56188
C	2.86058	-0.89926	-0.04752
H	2.44983	-1.90684	-0.01884
C	4.23019	-0.66032	-0.08252
C	4.65665	0.67042	-0.13669
H	5.71575	0.93074	-0.16761
N	3.78990	1.69275	-0.15377
C	2.49548	1.38751	-0.11863
C	1.44372	2.37095	-0.14179
C	0.03959	4.05248	-0.26506
C	-0.53862	5.38581	-0.37103
C	0.11497	6.53375	0.11482
H	1.09225	6.45590	0.58593
C	-0.50077	7.77866	0.00951
H	0.00594	8.66294	0.39378
C	-1.76259	7.89492	-0.58056
H	-2.23551	8.87271	-0.66573
C	-2.41892	6.75623	-1.05847
H	-3.40225	6.84583	-1.51857
C	-1.81577	5.50662	-0.95234

H	-2.31603	4.61325	-1.32277
C	2.50665	4.62659	-0.44939
C	3.40294	4.85161	0.59437
H	3.27497	4.33894	1.54686
C	4.45739	5.73988	0.39891
H	5.16172	5.91864	1.21179
C	4.62827	6.41130	-0.82380
C	3.71135	6.15572	-1.85550
H	3.82967	6.65927	-2.81511
C	2.65164	5.26790	-1.67808
H	1.93893	5.08137	-2.48063
C	5.75132	7.39313	-1.01156
H	5.98155	7.54400	-2.07273
H	6.66396	7.06156	-0.50044
H	5.48178	8.37318	-0.58957
H	4.93788	-1.48652	-0.06938

LS Fe(L^{pyridine})$_3$(BF$_4$)$_2$

Fe	0.00000	0.00000	0.00000
N	0.24772	-0.24190	-1.94558
N	-0.10316	-1.89859	-0.07380
N	-0.29503	-2.85498	0.86273
N	-0.16649	-3.79810	-1.15235
N	-0.07637	-0.03643	1.96725
N	1.87615	-0.04769	0.36682
N	2.98919	-0.06168	-0.41007
N	3.57517	-0.25643	1.73198
N	-0.10316	1.97161	-0.07380
N	-1.88575	0.19327	-0.25192
N	-2.90347	-0.68706	-0.42703
N	-3.68982	1.39380	-0.57841
C	0.45420	0.73074	-2.85071
H	0.52558	1.74265	-2.45977
C	0.57432	0.47645	-4.21415
H	0.74396	1.30436	-4.89973
C	0.46945	-0.83778	-4.67277
H	0.54895	-1.06494	-5.73471
C	0.26518	-1.86088	-3.75002
H	0.18316	-2.89820	-4.06545
C	0.16905	-1.54005	-2.39424
C	-0.02039	-2.44293	-1.28801
C	-0.34182	-4.02126	0.20931
C	-0.56699	-5.30936	0.85532
C	-1.30669	-5.32269	2.05369
H	-1.69819	-4.38444	2.44343
C	-1.53984	-6.52320	2.71821
H	-2.12051	-6.52717	3.64001
C	-1.03765	-7.72166	2.20107
H	-1.22389	-8.66088	2.72091
C	-0.29415	-7.71316	1.01793
H	0.10907	-8.64336	0.61945
C	-0.05685	-6.51675	0.34387
H	0.53747	-6.52478	-0.56693
C	-0.21323	-4.75771	-2.22178
C	0.96420	-5.11989	-2.87599
H	1.91428	-4.67946	-2.57346
C	0.90600	-6.05654	-3.90883
H	1.82419	-6.34367	-4.42169
C	-0.31012	-6.64445	-4.29073
C	-1.47773	-6.25875	-3.60930
H	-2.43309	-6.70400	-3.88891
C	-1.43888	-5.31954	-2.58166
H	-2.34448	-5.02996	-2.05021
C	-0.36881	-7.66895	-5.38982
H	0.60419	-7.79458	-5.87804
H	-1.10420	-7.38642	-6.15567
H	-0.67812	-8.64733	-4.99472
C	-1.19539	-0.03949	2.71304

H	-2.13395	0.00061	2.16624
C	-1.17041	-0.10960	4.10299
H	-2.10825	-0.11024	4.65501
C	0.05904	-0.18012	4.76033
H	0.10770	-0.22852	5.84698
C	1.22875	-0.19715	4.00438
H	2.20578	-0.26383	4.47664
C	1.13402	-0.13514	2.61266
C	2.21075	-0.16098	1.65465
C	4.03219	-0.18757	0.41914
C	5.42264	-0.22146	-0.02071
C	6.43286	-0.88808	0.69709
H	6.20149	-1.41322	1.62064
C	7.73949	-0.89816	0.21265
H	8.51485	-1.42156	0.77073
C	8.05467	-0.24864	-0.98365
H	9.07913	-0.25573	-1.35432
C	7.05273	0.40698	-1.70633
H	7.29506	0.91292	-2.64020
C	5.74430	0.42049	-1.23269
H	4.95749	0.92860	-1.78798
C	4.34590	-0.36127	2.94203
C	4.37033	-1.57146	3.63528
H	3.81251	-2.42812	3.25710
C	5.12350	-1.66698	4.80602
H	5.14790	-2.61171	5.34950
C	5.85896	-0.57353	5.28992
C	5.81295	0.63114	4.56647
H	6.37750	1.49300	4.92351
C	5.06111	0.74613	3.39969
H	5.03694	1.68014	2.83958
C	6.68104	-0.68107	6.54423
H	6.59977	-1.67561	6.99703
H	6.36245	0.06168	7.28903
H	7.74254	-0.48781	6.33430
C	0.93314	2.82889	0.02766
H	1.91432	2.37532	0.14707
C	0.77787	4.21011	-0.03870
H	1.65337	4.85111	0.04520
C	-0.50001	4.74576	-0.21923
H	-0.64918	5.82260	-0.27939
C	-1.58567	3.87982	-0.32389
H	-2.59564	4.25673	-0.46710
C	-1.36448	2.50228	-0.24422
C	-2.33961	1.44704	-0.34623
C	-4.00838	0.04047	-0.62926
C	-5.32495	-0.53288	-0.88405
C	-6.52176	0.12673	-0.54894
H	-6.50093	1.10595	-0.07669
C	-7.74794	-0.48505	-0.80211
H	-8.67012	0.02879	-0.53368
C	-7.79742	-1.75196	-1.38923

H	-8.75931	-2.22308	-1.58851
C	-6.61077	-2.41558	-1.71714
H	-6.64719	-3.40369	-2.17446
C	-5.38129	-1.81448	-1.46572
H	-4.45097	-2.31997	-1.71944
C	-4.55232	2.52186	-0.80842
C	-4.95873	3.30975	0.27155
H	-4.62771	3.06426	1.28070
C	-5.79739	4.39757	0.04004
H	-6.11883	5.01230	0.88144
C	-6.24557	4.70899	-1.25644
C	-5.81850	3.89757	-2.31857
H	-6.15431	4.12147	-3.33122
C	-4.97360	2.80778	-2.10529
H	-4.65284	2.17781	-2.93412
C	-7.16649	5.87464	-1.48782
H	-7.35748	6.03246	-2.55513
H	-6.74561	6.80073	-1.07260
H	-8.13372	5.71200	-0.99084

LS Fe(L^pyrazine)_3(BF_4)_2

Fe	0.00000	0.00000	0.00000
C	-0.81156	-2.87412	0.02579
H	-1.82979	-2.49902	0.10114
C	-0.53226	-4.24087	0.00600
H	-1.34393	-4.96666	0.07425
C	1.70034	-3.82607	-0.18872
H	2.72019	-4.20387	-0.26890
C	1.45650	-2.44594	-0.18760
C	2.40297	-1.36817	-0.28830
C	4.03003	0.09254	-0.52864
C	5.32785	0.72697	-0.71866
C	5.48849	2.03961	-0.22828
H	4.65990	2.51651	0.29310
C	6.69791	2.70564	-0.39735
H	6.81865	3.71476	-0.00509
C	7.75952	2.07796	-1.05797
H	8.70771	2.59921	-1.18523
C	7.60338	0.78097	-1.55370
H	8.42520	0.29374	-2.07680
C	6.39721	0.10294	-1.38807
H	6.28864	-0.89975	-1.79362
C	4.64990	-2.38603	-0.65961
C	4.68017	-3.08820	-1.86446
H	4.04331	-2.78044	-2.69379
C	5.54267	-4.17754	-1.99136
H	5.57058	-4.72963	-2.93084
C	6.37859	-4.57134	-0.93424
C	6.32298	-3.84019	0.26577
H	6.96280	-4.12938	1.09994
C	5.46426	-2.75331	0.41245
H	5.43028	-2.18868	1.34355
C	7.31550	-5.73797	-1.07557
H	7.18725	-6.24606	-2.03778
H	7.15423	-6.47227	-0.27435
H	8.36192	-5.40872	-1.00159
C	-0.47745	-0.67461	-2.86056
H	-0.72366	-1.67202	-2.50411
C	-0.50287	-0.35219	-4.21818
H	-0.77246	-1.10877	-4.95633
C	0.10433	1.79875	-3.76225
H	0.33801	2.80222	-4.11947
C	0.13022	1.51244	-2.39076
C	0.37291	2.40153	-1.28848
C	0.83799	3.93773	0.21027
C	1.21786	5.18314	0.86500
C	0.69999	5.43355	2.15131
H	0.02242	4.70919	2.60027
C	1.04638	6.59726	2.83081
H	0.63514	6.78757	3.82144
C	1.91410	7.52276	2.24204

H	2.18120	8.43525	2.77400
C	2.43990	7.27483	0.97135
H	3.12439	7.98878	0.51488
C	2.09800	6.11364	0.28107
H	2.52911	5.92876	-0.69969
C	1.17304	4.54839	-2.25181
C	0.32912	5.58535	-2.64312
H	-0.60723	5.75815	-2.11397
C	0.71308	6.40089	-3.70801
H	0.06067	7.21840	-4.01510
C	1.92179	6.19043	-4.39027
C	2.74849	5.13281	-3.97038
H	3.69522	4.95652	-4.48191
C	2.38492	4.31159	-2.90566
H	3.03307	3.50186	-2.56934
C	2.32037	7.06024	-5.54980
H	1.71876	7.97518	-5.59343
H	2.18325	6.52437	-6.50088
H	3.37936	7.34344	-5.48987
C	1.03347	0.20437	2.77070
H	2.01664	0.21276	2.30680
C	0.86560	0.35244	4.14807
H	1.73523	0.45986	4.79772
C	-1.40171	0.25744	3.92559
H	-2.39007	0.28972	4.38500
C	-1.27021	0.10038	2.53934
C	-2.29985	0.00493	1.54021
C	-4.05281	-0.11740	0.21690
C	-5.41640	-0.16797	-0.29371
C	-6.48750	-0.69852	0.44984
H	-6.32584	-1.09656	1.44869
C	-7.76531	-0.73728	-0.10379
H	-8.58842	-1.15577	0.47394
C	-7.99115	-0.25024	-1.39406
H	-8.99370	-0.27939	-1.81944
C	-6.92909	0.27098	-2.14012
H	-7.10282	0.65061	-3.14625
C	-5.64823	0.31135	-1.59844
H	-4.81605	0.71870	-2.17024
C	-4.49463	0.04519	2.72766
C	-4.60636	-1.05908	3.57230
H	-4.08471	-1.98612	3.33426
C	-5.40142	-0.96078	4.71460
H	-5.49192	-1.82121	5.37768
C	-6.09216	0.22247	5.02057
C	-5.95796	1.31582	4.14656
H	-6.48470	2.24501	4.36577
C	-5.16347	1.23799	3.00530
H	-5.06739	2.08764	2.33031
C	-6.96121	0.32264	6.24286
H	-6.88493	-0.57386	6.86817
H	-8.01623	0.45078	5.96075

H	-6.68523	1.19331	6.85331
N	0.17276	-1.95904	-0.06648
N	0.71608	-4.72372	-0.09334
N	1.91021	-0.13316	-0.16888
N	3.74985	-1.27043	-0.51575
N	2.89830	0.78048	-0.32093
N	-0.02586	0.06886	1.95746
N	-0.34244	0.37903	4.72973
N	-1.89328	-0.03895	0.26810
N	-3.66742	-0.04358	1.55233
N	-2.96104	-0.11917	-0.56201
N	-0.14943	0.24521	-1.93943
N	-0.20379	0.87332	-4.67462
N	0.17276	1.90539	-0.06648
N	0.79919	3.69189	-1.15834
N	0.45154	2.83683	0.86983

LS Fe($L^{pyridazine}$)$_3$(BF_4)$_2$

Fe	0.00000	0.00000	0.00000
N	0.80031	0.01030	-2.76020
N	-0.22815	0.04585	-1.92921
N	-1.90834	0.07712	-0.06097
N	-2.87979	0.08049	0.87434
N	-3.80011	0.21061	-1.15164
N	-0.81753	2.75423	0.06695
N	0.21609	1.92907	0.06420
N	1.91216	0.07712	-0.06097
N	2.89026	-0.84990	-0.12268
N	3.79927	1.18246	-0.00798
N	0.05721	0.81169	2.74828
N	0.04189	-0.22292	1.92366
N	-0.01576	-1.91364	0.06689
N	-0.04340	-2.89053	-0.86289
N	0.08904	-3.80093	1.16732
C	0.56141	-0.01019	-4.07592
H	1.45445	-0.04634	-4.69914
C	-0.72437	0.01308	-4.62928
H	-0.86545	-0.01324	-5.70819
C	-1.80189	0.07888	-3.76022
H	-2.83057	0.11299	-4.11238
C	-1.52422	0.09908	-2.39016
C	-2.43892	0.14432	-1.28295
C	-4.04462	0.16600	0.21658
C	-5.34829	0.18773	0.86845
C	-6.48065	0.78887	0.28798
H	-6.41470	1.27059	-0.68447
C	-7.69451	0.79249	0.97215
H	-8.56466	1.26645	0.51961
C	-7.79600	0.20145	2.23412
H	-8.74873	0.20486	2.76256
C	-6.67161	-0.38864	2.82028
H	-6.74708	-0.84672	3.80583
C	-5.45420	-0.39521	2.14663
H	-4.57242	-0.85035	2.59458
C	-4.74179	0.24722	-2.23728
C	-5.47433	-0.89865	-2.55101
H	-5.33696	-1.80870	-1.96819
C	-6.38574	-0.85211	-3.60313
H	-6.96379	-1.74402	-3.84688
C	-6.57581	0.32118	-4.35446
C	-5.81813	1.45419	-4.01856
H	-5.95222	2.37569	-4.58549
C	-4.90557	1.42729	-2.96310
H	-4.33346	2.31516	-2.69406
C	-7.57425	0.35651	-5.47792
H	-7.38727	-0.45064	-6.19956
H	-7.54560	1.31110	-6.01523
H	-8.59548	0.21343	-5.09639

C	-0.58895	4.07150	0.10938
H	-1.48734	4.68803	0.12154
C	0.69229	4.63434	0.12950
H	0.82500	5.71420	0.15972
C	1.77709	3.77201	0.09635
H	2.80420	4.13061	0.09206
C	1.50914	2.40069	0.06863
C	2.43524	1.30166	0.01709
C	4.05127	-0.18219	-0.10067
C	5.35848	-0.82136	-0.18557
C	6.51895	-0.26133	0.37993
H	6.47334	0.68509	0.91376
C	7.73584	-0.93225	0.27644
H	8.62943	-0.49637	0.72155
C	7.81122	-2.15950	-0.38770
H	8.76687	-2.67626	-0.47001
C	6.65924	-2.72408	-0.94474
H	6.71600	-3.67971	-1.46480
C	5.43820	-2.06378	-0.84498
H	4.53585	-2.48986	-1.28092
C	5.39416	2.60044	-1.20405
H	5.19589	2.02967	-2.11043
C	4.73835	2.27124	-0.01673
C	4.98095	2.98013	1.15993
H	4.47001	2.69974	2.08114
C	5.89318	4.03612	1.14047
H	6.08822	4.59058	2.05866
C	6.57400	4.38813	-0.03564
C	6.30710	3.65219	-1.20361
H	6.82492	3.90802	-2.12852
C	7.57051	5.51374	-0.05193
H	7.31416	6.25768	-0.81894
H	8.57647	5.14106	-0.29250
H	7.62134	6.02359	0.91669
C	0.03612	0.58178	4.06604
H	0.04173	1.47963	4.68338
C	0.01047	-0.69997	4.62759
H	-0.01424	-0.83298	5.70752
C	0.02812	-1.78515	3.76464
H	0.02589	-2.81241	4.12273
C	0.04904	-1.51674	2.39361
C	0.05454	-2.43866	1.29121
C	0.02444	-4.05291	-0.19882
C	0.01265	-5.36030	-0.84357
C	-0.58445	-5.46037	-2.11565
H	-1.02572	-4.57158	-2.56365
C	-0.60971	-6.68100	-2.78304
H	-1.07901	-6.75188	-3.76363
C	-0.03793	-7.81461	-2.19649
H	-0.06010	-8.77002	-2.71965
C	0.56686	-7.71916	-0.94056
H	1.02580	-8.59702	-0.48742

C	0.59536	-6.50201	-0.26277
H	1.08695	-6.44143	0.70507
C	0.13745	-4.73396	2.26022
C	1.33689	-4.91032	2.95517
H	2.22561	-4.35543	2.65422
C	1.37813	-5.81125	4.01681
H	2.31357	-5.95501	4.55862
C	0.23991	-6.54710	4.39316
C	-0.94849	-6.34451	3.67485
H	-1.84226	-6.90448	3.95048
C	-1.00984	-5.44160	2.61280
H	-1.93425	-5.29469	2.05548
C	0.30611	-7.53151	5.52766
H	0.98472	-8.36173	5.28422
H	-0.67884	-7.95587	5.75219
H	0.69394	-7.05929	6.44077

References

1. F. Neese, *Wiley Interdiscip. Rev. Comput. Mol. Sci.*, 2012, **2**, 73-78.
2. S. Rodríguez-Jiménez and S. Brooker, *Inorg. Chem.*, 2017, **56**, 13697-13708.
3. S. Ye and F. Neese, *Inorg. Chem.*, 2010, **49**, 772-774.
4. E. Caldeweyher, C. Bannwarth and S. Grimme, *J. Chem. Phys.*, 2017, **147**, 034112.
5. Y. Takano and K. N. Houk, *J. Chem. Theory Comput.*, 2005, **1**, 70-77.
6. K. Burke, J. P. Perdew and Y. Wang, in *Electronic Density Functional Theory*, Springer, 1998, pp. 81-111.
7. A. D. Becke, *Phys. Rev. A*, 1988, **38**, 3098-3100.
8. F. Weigend and R. Ahlrichs, *Phys. Chem. Chem. Phys.*, 2005, **7**, 3297-3305.
9. F. Weigend, *Phys. Chem. Chem. Phys.*, 2006, **8**, 1057-1065.
10. G. t. Te Velde, F. M. Bickelhaupt, E. J. Baerends, C. Fonseca Guerra, S. J. van Gisbergen, J. G. Snijders and T. Ziegler, *J. Comput. Chem.*, 2001, **22**, 931-967.
11. T. A. Albright, J. K. Burdett and M.-H. Whangbo, *Orbital interactions in chemistry*, John Wiley & Sons, 2013.
12. E. Baerends, V. Branchadell and M. Sodupe, *Chem. Phys. Lett.*, 1997, **265**, 481-489.
13. S. Rodríguez-Jiménez, M. Yang, I. Stewart, A. L. Garden and S. Brooker, *J. Am. Chem. Soc.*, 2017, **139**, 18392–18396.
